# The worldwide impact of COVID-19 on cancer care: A meta-analysis of surveys published after the first wave of the pandemic

**DOI:** 10.3389/fonc.2022.961380

**Published:** 2022-09-29

**Authors:** Serena Di Cosimo, Nicola Susca, Giovanni Apolone, Nicola Silvestris, Vito Racanelli

**Affiliations:** ^1^ Platform of Integrated Biology Unit, Department of Applied Research and Technological Development, Fondazione IRCCS Istituto Nazionale dei Tumori, Milan, Italy; ^2^ School of Medicine: Interdisciplinary of Medicine, Aldo Moro University of Bari, Bari, Italy; ^3^ Scientific Directorate, Fondazione IRCCS Istituto Nazionale Tumori, Milan, Italy; ^4^ Medical Oncology Unit, Department of Human Pathology “G. Barresi”, University of Messina, Messina, Italy

**Keywords:** oncology, COVID-19, survey, healthcare, meta-analysis

## Abstract

**Background:**

The rapid and global spread of COVID-19 posed a massive challenge to healthcare systems, which came across the need to provide high-intensity assistance to thousands of patients suffering from SARS-CoV-2 infection while assuring continuous care for all other diseases. This has been of particular importance in the oncology field. This study explores how oncology centers responded to the pandemic at a single center level by assessing surveys addressing different aspects of cancer care after the pandemic outbreak.

**Methods:**

We performed a systematic review and meta-analysis of the cancer care surveys published until December 11th, 2020. Data were analyzed according to three main areas of interest, namely health care organization, including cancellation/delay and/or modification of scheduled treatments, cancellation/delay of outpatient visits, and reduction of overall cancer care activities; routine use of preventive measures, such as personal protective equipment (PPE) by both patients and health care workers, and systematic SARS-CoV-2 screening by nasopharyngeal swabs; and implementation of telemedicine through remote consultations.

**Findings:**

Fifty surveys reporting data on 9150 providers from 121 countries on 5 continents were included. Cancellation/delay of treatment occurred in 58% of centers; delay of outpatient visits in 75%; changes in treatment plans in 65%; and a general reduction in clinical activity in 58%. Routine use of PPE by patients and healthcare personnel was reported by 81% and 80% of centers, respectively; systematic SARS-CoV-2 screening by nasopharyngeal swabs was reported by only 41% of centers. Virtual visits were implemented by the majority (72%) of centers.

**Interpretation:**

These results describe the negative impact of COVID-19 on cancer care, the rapid response of cancer centers in terms of preventive measures and alternative treatment approaches such as telemedicine, and confirm that surveys can provide the valuable, low-cost and immediate information that critical situations require.

## Introduction

COVID-19 has posed unprecedented challenges to both individuals and society (1, 2). Responding to the pandemic requires decisions based on accurate real-time information. In order to understand and anticipate the demand on health care services, there is the urgent need to know not only the spread of the virus but especially the response to its spread (3, 4). Once in a lifetime, the need to rapidly collect and evaluate data has never been more apparent, allowing decision-makers the ability to move quickly and effectively in a crisis. Real-time data can be managed with the survey tools cheaply (5). In health epidemiology, the most prevalent sort of survey is represented by online survey. Even sophisticated surveys can be cost-effective because of the availability of specialized and easy-to-use software for survey development and distribution (6). The absence of involved interviewer(s) increases the availability of respondent sharing. There is no universally agreement upon minimum response for surveys, and response rates for online surveys range from 20% to 30% (7).

Surveys have recently become quite popular, especially among oncologists, to gather data on behavioral, policy, and healthcare system responses to the pandemic. Individual or aggregate information, mostly focused on patient management and protection of health workers, was collected with the ultimate goal of descriptive analyses (8, 9). Given the possible volume and timeliness of the data generated, it is worthwhile to explore how the survey design and conduct may have influenced their generalizability. For example, in terms of study design, sampling challenges may develop due to accessibility or the low involvement of less motivated groups (i.e., selection bias) [reviewed in Andrade (10)]. In addition, external factors such as geographic area may have influenced survey results even with the same study design and implementation. Herein, we report a systemic review and meta-analysis of primary data from surveys on COVID-19 and its impact on oncological practice, specifically health care organization, promotion of patients and health workers preventive measures, and implementation of telemedicine.

## Methods

### Search strategy, selection criteria, and data extraction

The current systematic review and meta-analysis was conducted according to the PRISMA guidelines. A literature search on PubMed, Scopus and Web Of Science was performed from databases inception to December 11, 2020. An example search string is provided in the Supplementary material. Titles and abstracts for potentially relevant articles were screened by two authors (V.R. and N.Su.). The conflict was resolved with a consensus between the two authors. All English-language articles reporting results of surveys on the SARS-CoV-2 pandemic impact on oncological clinical practice or on the use of countermeasures to limit this impact were considered relevant for inclusion. The articles were screened by Rayyan software. The following data were extracted for each study: first author name, month and year of publication, week of survey beginning and end, country of survey dissemination, the specialty of respondents, type of cancer, a center/operator categorization variable, number of recipients, and respondents, survey dissemination modality, number of questions, country income according to the World Bank Group and the mean Government Response Stringency Index (GRSI) developed by the Blavatnik School of Government at the University of Oxford during the study period. The outcomes of interest were expressed as proportions of respondents over recipients and included: cancellation/delay of treatments, modification of treatments, cancellation/delay of clinic visits, reduction of activity, routine personal protective equipment (PPE) use by patients and workers, use of remote consultations, and systematic execution of screening SARS-CoV-2 nasopharyngeal swab. These domains were chosen *a priori* to analyze the response of health care system to two different and complementary needs posed by the pandemic, specifically the continuity of cancer care and the containment of infection of both patients and health care workers. Only studies reporting the specialty of interest (oncology, or surgery, or radiotherapy) were included. The results were analyzed according to the volume and typology of the surveys reported and geographical area.

### Data analysis

A random-effects meta-analysis was performed using the DerSimonian-Laird estimator for variance. The I^2^ (the proportion of the variance in observed effects reflecting variance in true effects rather than sampling error) and p-value (the probability value describing how likely it is that data would have occurred under the null hypothesis of statistical test) for the Q-test (based on a chi-square distribution to generate a probability, that, when large, indicates larger variation across studies rather than within subjects within a study) were chosen as measures of heterogeneity. Proportions were transformed with double-arcsine transformation to approximate a normal distribution and then back-transformed to facilitate data interpretation. Since a high level of heterogeneity was found for all outcomes of interest, a thorough moderator analysis was performed to account for this heterogeneity with subgroup analysis and meta-regression. The following covariates were used as moderator variables: specialty, geographical area of survey dissemination, week of study beginning, week of study end, study sample size, and the center/operator categorization variable. Regarding geographical area subgroup analysis and meta-regression, studies conducted worldwide were excluded because it was impossible to assign them to a specific geographical area. The survey by Balakrishnan et. al (11). was excluded from this analysis for the same reason.

Publication bias was assessed by visual inspection of funnel plots and by the test of Egger. A p-value ≤0.05 was considered statistically significant for all analyses.

A qualitative evaluation of the risk of bias of the studies was performed using the “Risk of bias instrument for cross-sectional surveys of attitudes and practices” from the CLARITY Group at McMaster University (12). All the analyses were performed using R software (R version 3.6.2, release date: 2019–12-12; R Foundation for Statistical Computing, Vienna, Austria).

### Role of the funding source

There was no funding source.

## Results

### Literature search

**Figure 1:**
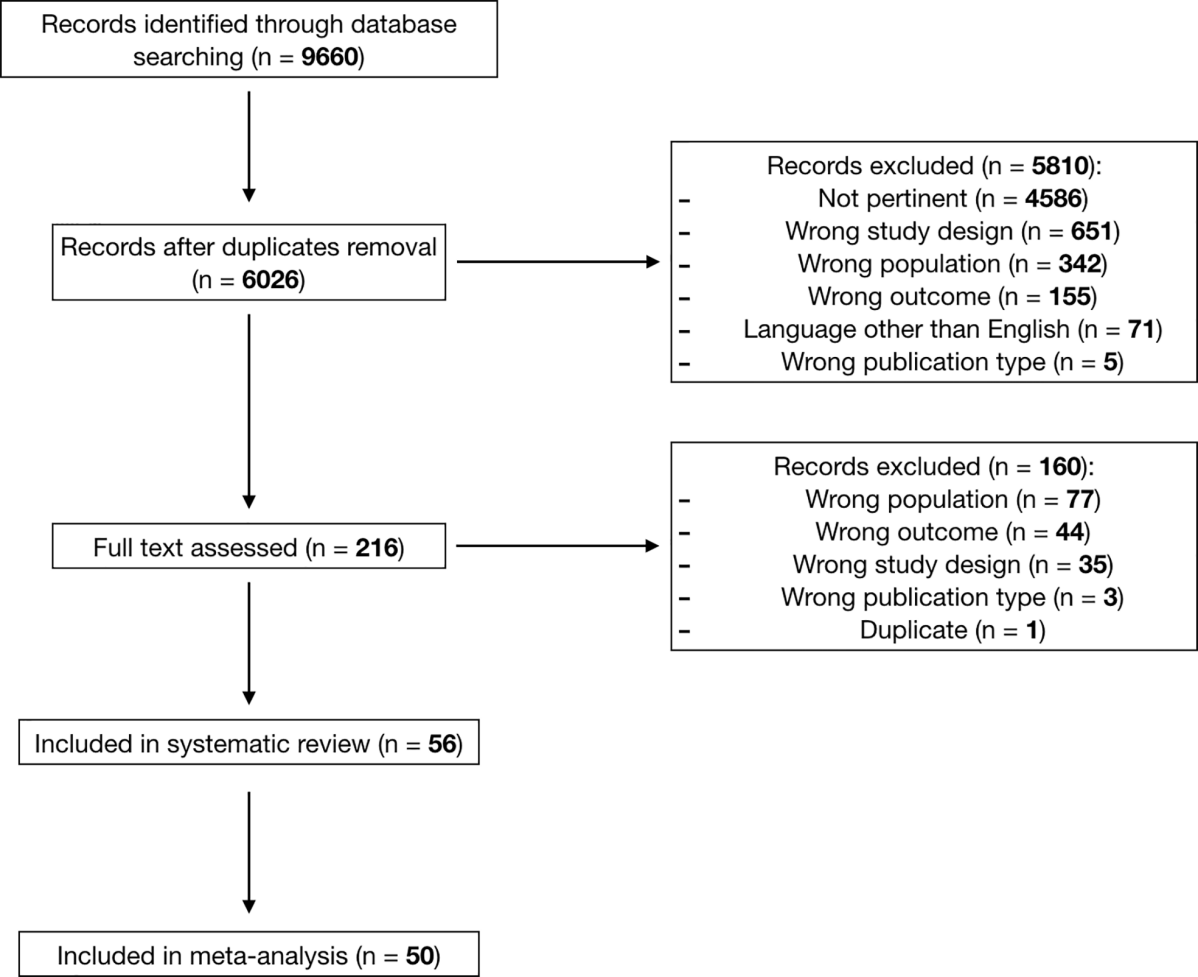
PRISMA flow-chart.

The literature search initially provided 9660 results, which, after duplicates removal, yielded 6026 publications. These were screened through title and abstract reading, providing 216 papers for full-text analysis. 56 articles (11, 13–67) were deemed eligible for the systematic review, and among these 50 reported quantitative data of at least one outcome of interest and were included in the meta-analysis (11, 18–66) (**Figure 1**).

### Studies characteristics

**Figure 2:**
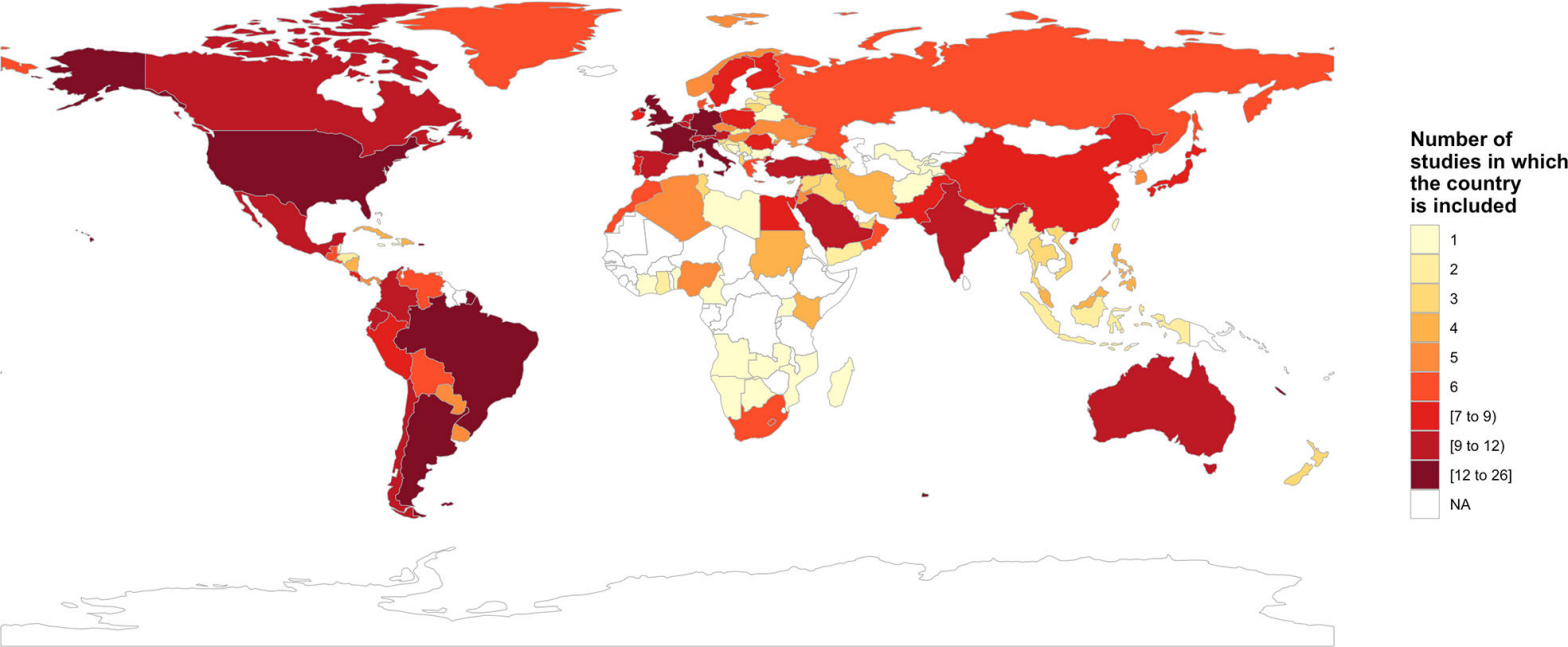
Worldwide distribution of the included studies.

**Table 1** shows the characteristics of the studies included in the systematic review. Answers from 1391 centers (1378 included in the quantitative synthesis) and 8386 operators (7772 included in the quantitative synthesis) from 121 countries were considered (**Figure 2**). Notably, the geographical distribution of the analyzed surveys roughly reflects that of COVID-19 spreading over the same study period. Italy was included in most surveys, twice as much as the second most commonly included country represented by Brazil. This parallels the spreading of COVID-19, as Italy was the most affected country during the first outbreak, followed by Brazil a few weeks later. China, where the pandemic originated, was not included among the top countries producing COVID-19 surveys. The lack of surveys from Africa may partly reflect a relative lack of dedicated oncology facilities and COVID-19 data collection compared to higher-income countries.

**Table 1:** Characteristics of the studies included in the systematic review.

Study	Weeks	Origin of survey	Region	Extent	Specialty	Neoplasm	Respondents/recipients	Survey modality	Questions	Clinical impact	Outcome impact	Personal impact	Countermeasures	Research impact
Achard, May 20 (18)	15-17	Switzerland	–	National	rt	–	22/30 (C)	email	53	Y	N	N	Y	N
Torzilli, Aug 20 (19)	13	Italy	–	National	sur	–	54/57 (C)	email	56	Y	N	N	Y	N
Mathiesen, Jul 20 (20)	14-15	Europe	–	Transnational	sur	Neurosurgery	25/34 (C)	Email-tel	–	Y	N	N	N	N
De Felice, Oct 20 (21)	21	Italy	–	National	rt	Head and neck	89/- (O)	email	30	Y	N	N	N	N
Nakayama, May 20 (22)	14-16	World	–	Transnational	sur	Gynecology	331/2305 (O)	email	–	Y	N	N	N	N
Rodriguez, Jun 20 (23)	15-16	South America	–	Transnational	sur	Gynecology	610/1052 (O)	email	22	Y	N	N	N	N
Aghemo, Jul 20 (24)	15-18	Italy	–	National	onc-sur	Liver	194/668 (O)	email	30	Y	N	N	N	N
Poggio, Apr 20 (13)	14-15	Italy		National	onc	Breast	165/2201 (O)	email	29	Y	N	N	N	Y
Jereczek-Fossa, Nov 20 (25)	24-25	Italy	Lombardy	Regional	rt	–	33/33 (C)	Google forms	–	Y	N	N	N	N
Gautam, Sep 20 (26)	12-27	India	–	Hospital	sur	–	13/15 (O)	email	28	Y	N	N	Y	N
Hui, Oct 20 (27)	13-15	USA	–	National	sur-rt-onc	–	411/- (O)	social media	–	Y	N	N	N	N
Martinelli, May 20 (28)	15-18	World	–	Transnational	sur-rt-onc	Gynecology	217/- (O)	social media-email	33	Y	N	N	Y	N
Gasparri, May 20 (29)	16-18	World	–	Transnational	sur-rt-onc	Breast	377/- (O)	–	35	Y	N	N	Y	N
Breccia, Jun 20 (30)	15	Italy	–	National	onc	CML	47/51 (C)	–	–	Y	N	N	Y	Y
Caricato, May 20 (31)	13	Italy	–	National	sur	Colo-rectal	39/43 (C)	–	25	Y	N	N	Y	N
Subbian, Jul 20 (15)	18-19	India	–	National	sur-rt-onc	Gynecology	148/- (O)	–	–	Y	N	N	Y	N
Nardin, Jul 20 (32)	15	France	–	National	onc-sur	Dermatology	21/52 (C)	email	11	Y	N	N	Y	N
Nunoo-Mensah, Jun 20 (33)	16-18	World	–	Transnational	sur	Colo-rectal	287/- (O)	social media-email	22	Y	N	N	Y	N
Jereczek-Fossa, May 20 (34)	15-16	Italy	–	National	rt	–	125/176 (C)	–	32	Y	N	N	Y	N
Martinez, Oct 20 (35)	19-22	South America	–	Transnational	rt	–	115/229 (C)	REDCap	26	Y	N	N	Y	N
Balakrishnan, Jun 20 (11)	15-18	Europe-Africa	–	Transnational	sur	Hepato-bilio-pancreatic	145/569 (O)	social media-email	14	Y	N	N	Y	N
Vasquez, May 20 (36)	15-16	South America	–	Transnational	sur-rt-onc	Pediatric	453/- (O)	email	–	Y	N	N	Y	N
Champagne, Dec 20 (37)	–	World	–	Transnational	sur	Neurosurgery-ENT	135/- (O)	website	–	Y	N	N	Y	N
Folkard, Apr 20 (14)	–	UK	Kent-Surrey-Sussex	Regional	sur	Urology	13/- (C)	email		Y	N	N	N	N
Gill, Sep 20 (38)	19-20	Canada	–	National	onc	–	159/618 (O)	email	–	Y	N	Y	Y	Y
Autràn-Gòmez, Jul 20 (39)	14-18	World	–	Transnational	sur	Urology	846/- (O)	social media-email	35	Y	N	N	Y	N
Brandes, May 20 (40)	–	Italy	Emilia-Romagna	Regional	onc	–	12/12 (C)	email	18	Y	N	N	Y	Y
Dotzauer, Jul 20 (41)	12-13	World	–	Transnational	sur-rt-onc	Urology	235/- (O)	social media	12	Y	N	N	N	N
Kumari, Sep 20 (42)	18-22	India	–	National	sur-rt-onc	Gynecology	61/- (O)	social media-email	20	Y	N	N	N	N
Bogani, Sep 20 (sur) (43)	15-17	Italy	–	National	sur-rt-onc	Gynecology	604/860 (O)	email	45	Y	N	N	Y	Y
Gill, Apr 20 (44)	14	Canada	–	National	onc	–	159/- (O)	email	23	Y	N	Y	Y	Y
Rouger-Gaudichon, Sep 20 (45)	17-19	France	–	National	onc	Pediatric	28/31 (C)	–	37	Y	N	N	N	N
Chazan, Dec 20 (46)	19-25	World	–	Transnational	sur-rt-onc	–	501/- (O)	email	23	Y	N	Y	N	N
Ottaviano, Jul 20 (47)	15-19	Italy	–	National	onc	–	75/75 (O)	email	25	Y	N	N	N	N
Rebecchi, Nov 20 (48)	–	Italy	–	National	sur	Esophagus	12/12 (C)	email	26	Y	N	N	Y	N
Bhandoria, Jun 20 (16)	14	India	–	National	sur-rt-onc	Gynecology	90/520 (O)	Email-whatsapp	–	Y	N	N	N	N
Saab, Jul 20 (49)	15-17	Middle East-India-North Africa	–	Transnational	sur-rt-onc	Pediatric	34/82 (C)	email	34	Y	N	Y	Y	N
Jazieh, Sep 20 (50)	17-19	World	–	Transnational	sur-rt-onc	–	356/- (C)	–	51	Y	N	N	N	N
Panzuto, Aug 20 (51)	–	Italy	–	National	onc	NET	24/- (C)	email	57	Y	N	N	N	N
Koffman, Aug 20 (17)	–	World	–	Transnational	onc	CLL	59/62 (O)	–	–	Y	N	N	Y	N
Tamari, May 20 (52)	15-17	Japan	–	National	rt	–	184/- (O)	–	29	Y	N	N	Y	N
Onesti, Aug 20 (53)	15-19	World	–	Transnational	onc	–	21/30 (C)	email	46	N	N	N	Y	N
Brunner, Jul 20 (54)	15-16	Germany	–	National	sur	Colo-rectal	101/- (C)	email		Y	N	N	Y	N
Sadler, Nov 20 (55)	13-16	World	–	Transnational	onc	Cardio-oncology	306/- (O)	email	20	Y	N	N	Y	N
Singh, Aug 20 (56)	21-22	India	–	National	sur	–	256/480 (O)	Email-social network-messages	–	Y	N	Y	Y	N
Rimmer, May 20 (57)	13-15	UK	–	National	sur	Gynecology	148/155 (C)	–	24	Y	N	Y	Y	N
Indini, Apr 20 (58)	11	Italy	–	National	onc	–	122/145 (C)	–	27	Y	N	N	Y	N
Hasford, Aug 20 (59)	–	Africa	–	Transnational		–	12/- (C)	–	16	N	N	N	Y	N
Depypere, Aug 20 (60)	16-17	World	–	Transnational	sur	Chest	409/1780 (O)	email	26	Y	N	Y	Y	N
Kamarajah, Jul 20 (61)	17-18	World	–	Transnational	sur	Esophago-gastric	184/- (O)	email	40	Y	N	N	Y	N
Marandino, Jul 20 (62)	–	Italy	–	National	onc	Genito-urinary	72/- (O)	–	–	Y	N	N	N	Y
Harke, Sep 20 (63)	19	Germany	–	National	sur	Urology	27/66 (C)	email	–	Y	N	N	Y	N
Tashkandi, Jun 20 (64)	14-17	Middle East-North Africa	–	Transnational	onc	–	222/- (O)	Email-social network-messages	20	Y	N	N	Y	N
Boufkhed, Nov 20 (65)	22-26	Middle East-North Africa	–	Transnational	onc	Palliative care	43/- (O)	email	–	Y	N	Y	Y	N
Gulia, Jul 20 (66)	17-18	India	–	National	sur-rt-onc	Sarcoma	194/- (O)	Email-social network-online classes	25	Y	N	N	Y	N
Thaler, May 20 (67)	15-17	World	–	Transnational	sur-rt-onc	Sarcoma	152/- (O)	email	20	Y	N	Y	Y	N

Rt, radiotherapy; sur, surgery; onc, oncology; CLL, chronic lymphatic leukemia; CML, chronic myelogenous leukemia; ENT, ear-nose-throat; NET, neuro-endocrine tumor; C, centers; O, operators; Y, yes; N, no.

The period of the included surveys ranged from the 11^th^ to the 27^th^ week of 2020, as there was no other eligible study according to the pre-specified selection criteria afterwards.

**Figure 3:**
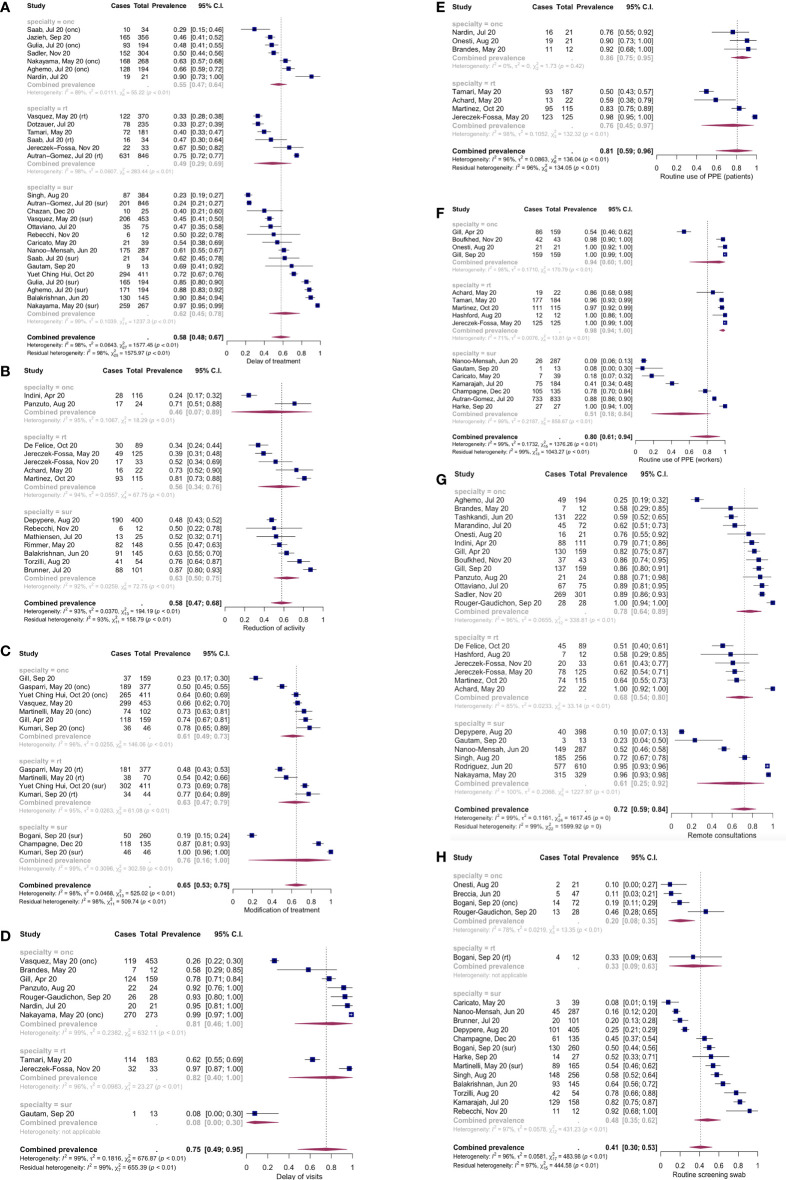
Forest plots of the meta-analysis results for the 8 outcomes. **(A)** Treatment delay/cancellation; **(B)** Reduction of activity; **(C)** Modification of treatment; **(D)** Delay of visits; **(E)** Routine use of PPE (patients); **(F)** Routine use of PPE (workers); **(G)** Use of remote consultations; **(H)** Routine screening SARS-CoV-2 swab.

Twenty-four surveys (42.9%) were conducted in more than one country, 28 (50%) were national, 3 (5.4%) were regional, and 1 (1.8%) was conducted at the single-hospital level. A wide range of cancer types was represented: brain, head and neck, gynecological, breast, hepato-bilio-pancreatic, hematological, colorectal, skin, pediatric, urinary tract, esophagogastric, neuroendocrine, lung and soft tissues cancers. 54 surveys (96.4%) dealt with the clinical impact of COVID-19 on oncology practice, 38 surveys (67.9%) with the countermeasures taken to limit SARS-CoV-2 spreading, 9 surveys (16.1%) with the impact it had on the workers personal well-being, and 7 surveys (12.5%) with the impact on the oncological research. None of the surveys considered the role of the pandemic on the outcome of oncological treatments. **Table 2** shows the summary of findings of the single studies included in the quantitative synthesis and **Figure 3** shows the meta-analysis results stratified according to the specialty (either oncology, surgery or radiotherapy) of each center/operator.

**Table 2:** Summary of the findings of the studies included in the meta-analysis. Rt, radiotherapy; sur, surgery; onc, oncology.

Study	Delay/cancellation of treatment	Delay of visits	Modification of treatment	Reduction of activity	Routine PPE use (patients)	Routine PPE use (workers)	Remote consultations	Routine screening SARS-CoV-2 swab
Achard, May 20 (18)	–	–	–	73%	59%	86%	100%	–
Torzilli, Aug 20 (19)	–	–	–	76%	–	–	–	78%
Mathiesen, Jul 20 (20)	–	–	–	52%	–	–	–	–
De Felice, Oct 20 (21)	–	–	–	34%	–	–	50%	–
Nakayama, May 20 (onc) (22)	63%	99%	–	–	–	–	–	–
Nakayama, May 20 (sur) (22)	97%	–	–	–	–	–	96%	–
Rodriguez, Jun 20 (23)	–	–	–	–	–	–	95%	–
Aghemo, Jul 20 (onc) (24)	66%	–	–	–	–	–	25%	–
Aghemo, Jul 20 (sur) (24)	88%	–	–	–	–	–	–	–
Jereczek-Fossa, Nov 20 (25)	67%	97%	–	52%	–	–	61%	–
Gautam, Sep 20 (26)	69%	8%	–	–	–	8%	23%	–
Hui, Oct 20 (sur) (27)	72%	–	73%	–	–	–	–	–
Hui, Oct 20 (onc) (27)	–	–	64%	–	–	–	–	–
Martinelli, May 20 (sur) (28)	–	–	–	–	–	–	–	54%
Martinelli, May 20 (onc) (28)	–	–	73%	–	–	–	–	–
Martinelli, May 20 (rt) (28)	–	–	54%	–	–	–	–	–
Gasparri, May 20 (onc) (29)	–	–	50%	–	–	–	–	–
Gasparri, May 20 (rt) (29)	–	–	48%	–	–	–	–	–
Breccia, Jun 20 (30)	–	–	–	–	–	–	–	11%
Caricato, May 20 (31)	54%	–	–	–	–	18%	–	8%
Nardin, Jul 20 (32)	90%	95%	–	–	76%	–	–	–
Nunoo-Mensah, Jun 20 (33)	61%	–	–	–	–	9%	52%	16%
Jereczek-Fossa, May 20 (34)	–	–	–	39%	98%	100%	62%	–
Martinez, Oct 20 (35)	–	–	–	81%	83%	97%	64%	–
Balakrishnan, Jun 20 (11)	90%	–	–	63%	–	–	–	64%
Vasquez, May 20 (onc) (36)	–	26%	66%	–	–	–	–	–
Vasquez, May 20 (sur) (36)	45%	–	–	–	–	–	–	–
Vasquez, May 20 (rt) (36)	33%	–	–	–	–	–	–	–
Champagne, Dec 20 (37)	–	–	87%	–	–	78%	–	45%
Gill, Sep 20 (38)	–	–	23%	–	–	100%	86%	–
Autran-Gomez, Jul 20 (sur) (39)	24%	–	–	–	–	88%	–	–
Autran-Gomez, Jul 20 (rt) (39)	75%	–	–	–	–	–	–	–
Brandes, May 20 (40)	–	58%	–	–	92%	–	58%	–
Dotzauer, Jul 20 (41)	33%	–	–	–	–	–	–	–
Kumari, Sep 20 (onc) (42)	–	–	78%	–	–	–	–	–
Kumari, Sep 20 (sur) (42)	–	–	100%	–	–	–	–	–
Kumari, Sep 20 (rt) (42)	–	–	77%	–	–	–	–	–
Bogani, Sep 20 (sur) (43)	–	–	19%	–	–	–	–	50%
Bogani, Sep 20 (onc) (43)	–	–	–	–	–	–	–	19%
Bogani, Sep 20 (rt) (43)	–	–	–	–	–	–	–	33%
Gill, Apr 20 (44)	–	78%	74%	–	–	54%	82%	–
Rouger-Gaudichon, Sep 20 (45)	–	93%	–	–	–	–	100%	46%
Chazan, Dec 20 (46)	40%	–	–	–	–	–	–	–
Ottaviano, Jul 20 (47)	47%	–	–	–	–	–	89%	–
Rebecchi, Nov 20 (48)	50%	–	–	50%	–	–	–	92%
Saab, Jul 20 (onc) (49)	29%	–	–	–	–	–	–	–
Saab, Jul 20 (sur) (49)	62%	–	–	–	–	–	–	–
Saab, Jul 20 (rt) (49)	47%	–	–	–	–	–	–	–
Jazieh, Sep 20 (50)	46%	–	–	–	–	–	–	–
Panzuto, Aug 20 (51)	–	92%	–	71%	–	–	88%	–
Tamari, May 20 (52)	40%	62%	–	–	50%	96%	–	–
Onesti, Aug 20 (53)	–	–	–	–	90%	100%	76%	10%
Brunner, Jul 20 (54)	–	–	–	87%	–	–	–	20%
Sadler, Nov 20 (55)	50%	–	–	–	–	–	89%	–
Singh, Aug 20 (56)	38%	–	–	–	–	–	72%	58%
Rimmer, May 20 (57)	–	–	–	55%	–	–	–	–
Indini, Apr 20 (58)	–	–	–	24%	–	–	79%	–
Hasford, Aug 20 (59)	–	–	–	–	–	100%	58%	–
Depypere, Aug 20 (60)	–	–	–	48%	–	–	10%	25%
Kamarajah, Jul 20 (61)	–	–	–	–	–	41%	–	82%
Marandino, Jul 20 (62)	–	–	–	–	–	–	63%	–
Harke, Sep 20 (63)	–	–	–	–	–	100%	–	52%
Tashkandi, Jun 20 (64)	–	–	–	–	–	–	59%	–
Boufkhed, Nov 20 (65)	–	–	–	–	–	98%	86%	–
Gulia, Jul 20 (onc) (66)	48%	–	–	–	–	–	–	–
Gulia, Jul 20 (sur) (66)	85%	–	–	–	–	–	–	–

### Effect of COVID-19 and cancer treatment schedules

Taken together, 58% (confidence interval, c.i. 48%-67%, I^2^ 98%, number of studies: 28 [11, 22, 24–27, 31–33, 36, 39, 41, 46–50, 52, 55, 56, 66)] reported cancellation or delay of treatments. Apparently, the stratification according to specialty did not modify this result, nor did any other of the moderator variables. Meta-regression considering the geographical area in which the survey was conducted and the week of survey beginning (n=17) seemed to explain a small amount of heterogeneity (R^2^ 15.9%, indicating the relative amount of heterogeneity explained by this variable, I^2^ 95.7%, indicating the residual heterogeneity), with the test of moderators in the meta-regression not being statistically significant (p=0.43).

Overall, 75% [c.i. 49%-95%, I^2^ 99%, number of studies: 10 (22, 25, 26, 32, 36, 40, 44, 45, 51, 52)] of studies reported delay of clinic visits. Stratification according to specialty seemed to show a lower proportion of visits delay in the surgery subgroup, but with only one study belonging to this subgroup. In the meta-regression analysis, the stratification of surgery vs oncology and radiotherapy yielded a tiny value of R^2^ (1.2%), with a p-value nearly reaching statistical significance (p=0.09). In contrast, the geographical area in which the study was conducted explains a higher amount of heterogeneity (n=9, R^2^ 87.3%, I^2^ 58%), with a p-value<0.0001 at the moderators’ test and a p-value for the Q-test for residual heterogeneity (which indicates whether the residual heterogeneity, or I^2^, is significantly different from 0) of 0.05 in the meta-regression. The analysis of geographical area with the study sample size explains all the heterogeneity, with no residual heterogeneity (n=9, R^2^ 100%, I^2^ 0%, p-value for the test of moderators <0.0001, p-value for the Q-test for heterogeneity=0.4).

Overall, 65% [c.i. 53%-75%, I^2^ 98%, number of studies: 14 (27–29, 36–38, 42–44)] of studies reported some modification of the treatment regimens or surgical interventions. The moderator variable which alone explained the most heterogeneity was the geographical area in which the study was conducted (n=9, R^2^ 31%, I^2^ 96.9%, p-value for the test of moderators in meta-regression=0.02). A model including geographical area and week of survey end accounted for a great part of heterogeneity (n=9, R^2^ 85.2%, I^2^ 87%, p-value for the test of moderators <0.0001, the p-value for the Q-test for residual heterogeneity <0.0001). Adding specialty to the model yielded an R^2^ of 100%, with no residual heterogeneity (n=9, the p-value for the test of moderators<0.0001, p-value for the Q statistic of residual heterogeneity=0.59).

Taken together, 58% [c.i. 47%-68%, I^2^ 93%, number of studies: 14 (11, 18–21, 25, 34, 35, 48, 51, 54, 57, 58, 60)] of the studies reported reduction in activity. None of the moderator variables taken alone seemed to explain the between-studies variance. A meta-regression model considering specialty and geographical area accounted for some of this heterogeneity (n=12, R^2^ 40.5%, I^2^ 88.3%, p-value for the test of moderators=0.2, Q-test for residual heterogeneity<0.0001). Adding the week of survey beginning to this model yielded similar results (n=10, R^2^ 43.2%, I^2^ 89.4%, p-value for the test of moderators=0.1, p-value for the Q-test for residual heterogeneity <0.0001).

#### Countermeasures to limit infection spread

Seven studies (18, 32, 34, 35, 40, 52, 53) reported information on the routine use of PPE by patients. No studies belonged to the surgery subgroup. Overall, routine PPE use by the patients was reported in 81% (c.i. 59%-96%, I^2^ 96%, number of studies: 7) of centers/operators. The moderator analysis showed that the geographical area alone was able to explain a minimal part of the true heterogeneity observed, but with significant residual heterogeneity (n=6, R^2^ 15.8%, I^2^ 90.2%, p-value for the test of moderators=0.5, p-value for the Q-test of residual heterogeneity<0.0001). Adding the sample size to this meta-regression model resulted in a significant explanation of true heterogeneity (n=6, R^2^ 77.4%, I^2^ 60.6%, p-value for the test of moderators=0.003, p-value for the Q-test for residual heterogeneity=0.08). GRSI alone explained a great part of the observed heterogeneity (n=5, R^2^ 74.2%, I^2^ 79.6%, p-value for the test of moderators=0.03, p-value for the Q-test of residual heterogeneity=0.002). Adding sample size to GRSI in the model further improved heterogeneity explanation (n=5, R^2^ 92.9%, I^2^ 43.8%, p-value for the test of moderators<0.0001, p-value for the Q-test of residual heterogeneity=0.17).

PPE were routinely used by workers in 80% [c.i. 61%-94%, I^2^ 99%, number of studies: 16 (18, 26, 31, 33–35, 37–39, 44, 52, 53, 59, 61, 63, 65)] of cases. No moderator was alone able to explain the true heterogeneity. A meta-regression model including geographical area, week of survey end and sample size accounted for a substantial part of this heterogeneity (n=10, R^2^ 78.9%, I^2^ 80.7%, p-value for test of moderators <0.0001, p-value for the Q-test for residual heterogeneity=0.006). Adding GRSI resulted in a better explanation of heterogeneity (n=8, R^2^ 98.7%, I^2^ 17.1%, p-value for test of moderators <0.0001, p-value for the Q-test for residual heterogeneity=0.27)

72% [c.i. 59%-84%, I^2^ 99%, number of studies (18, 21–26, 33–35, 38, 40, 44, 45, 47, 51, 53, 55, 56, 58–60, 62, 64, 65)] of the surveyed centers/operators reported use of remote consultations. None of the moderators accounted for a substantial part of this heterogeneity. A meta-regression model including geographical area, specialty, sample size and the center/operator categorization seemed to explain a part of the true heterogeneity, but without statistical significance (n=20, R^2^ 29.7%, I2 92.3%, p-value for the test of moderators=0.39, p-value for the Q-test for residual heterogeneity<0.0001).

18 studies (11, 19, 28, 30, 31, 33, 37, 43, 45, 48, 53, 54, 56, 60, 61, 63) reported information on the systematic screening SARS-CoV-2 PCR on naso-pharingeal swab execution on patients. Systematic swab execution was reported in 41% (c.i. 30%-53%, I^2^ 96%, number of studies: 18) of studies. None of the moderators was able alone to account for the true heterogeneity. A meta-regression model including specialty, sample size and center/operator categorization explained part of the heterogeneity (n=17, R^2^ 24.6%, I^2^ 95.6%, p-value for the test of moderators=0.13, p-value for the Q-test for residual heterogeneity <0.0001).

### Publication bias

Publication bias was detected only for the modification of treatments outcome (Egger test p-value=0.03).

## Discussion

This systematic review and meta-analysis attempt to sketch the worldwide response to the COVID-19 pandemic in the oncology field. We chose to investigate the first months of the pandemic because it appeared more fascinating to us to analyze the first moves of an unprepared globe. At the point when this meta-analysis was conducted, most studies did not specifically report the prevalence of COVID-19 among cancer patients. None of the included studies reported outcomes of COVID-19 patients based on their underlying oncological disease (68). In this context, our work offers several insights for the implementation of surveys in the oncology field. Firstly, it is possible to evaluate the qualitative and quantitative information of surveys in a rigorous manner; secondly, surveys can provide prompt information on what is happening in the real-world; finally, this information is certainly heterogeneous, although it can be generalized according to specific domains, as in our specific case to meet the needs of cancer patients, their protection from infection and those of their care providers.

Our findings are consistent with current literature data that COVID-19 constrained cancer care (69–76). Overall, it seems that the effects of the pandemic on clinical practice were comparable across the three considered subspecialties, with more than half of centers reporting cancellation and delay in the delivery of treatments. More broadly, a decline in activity was seen particularly affecting medical treatment in lower-income geographical area (77–79). These data are consistent with healthcare authorities policy to advise hospitals and healthcare facilities to delay medical care for non-acute or not life-threatening conditions and to postpone cancer screenings while tackling the pandemic. According to a report by the World Health Organization, healthcare services for non-communicable diseases have been severely disrupted since the COVID-19 pandemic began (80).

Indeed, an increasing body of institutional but also nationwide and international evidence points towards major detrimental effects of the COVID-19 pandemic on several areas of healthcare including the provision of cancer care.

On the other hand, we examined the countermeasures taken to limit the intra-hospital infection spread. The routine use of PPE among patients and workers has been consistent, with about 80% of centers/operators reporting implementing this practice. Early precautionary measures were taken heterogeneously depending on country income level, consistently to already reported analyses (81). The proportions of respondents having noted an establishment of a remote consultation plan parallel that of the respondents reporting a delay in visits to the clinic. Thus, we can argue that the delay in visits has created the need for an alternative system to in-person consultation, and telemedicine has been the first response in many centers (82, 83).

Conversely, major efforts have been made not to defer active treatments. Finally, execution of a routine screening nasopharyngeal swab for asymptomatic patients has not been a widespread practice. Many centers reported using procedures like phone or in-person triage for suggestive COVID-19 symptoms instead. We chose not to quantify this response because it was expected to be largely implemented worldwide.

There are several strengths in our meta-analysis. To the best of our knowledge, this is the most comprehensive description of how cancer centers react to a rapidly evolving setting, in which researchers and medical professionals are continuously learning and contributing to dynamic adjustments in government policy. In this meta-analysis, we conducted a systematic search of the literature using a pre-defined inclusion and exclusion criteria; including a large number of studies to allow assessment of publication bias and subgroup analysis, detailed extraction of data on study outcomes; and the use of various statistical methods to evaluate the validity of our findings. This approach is crucial to manage the infodemic and promote the timely dissemination of accurate information, based on science and evidence, to all communities, particularly high-risk groups (84, 85).

Our work suffers the major issue of high level of heterogeneity among studies. We attempted to mitigate this limitation by performing subgroup analysis based on the characteristics of each study. Most of the included studies were limited by their small sample size. In addition, we considered data from countries with very different health systems response capacities and the surveys looked at them at different periods. This includes a variation in the temporary COVID-19 prevalence and incidence, as well as a difference in pandemic preparedness. Despite this, it seems that high heterogeneity is an issue intrinsic to meta-analyses of proportions. A systematic review of meta-analyses of proportion reports that about three-quarters of included studies had an I^2^ value of at least 90% (86).

Notwithstanding this limitation, we think that this systematic review and meta-analysis gives a particular piece of information on how oncology systems responded to the pandemic at a single-center level. The evaluation of local government guidelines, in fact, may be poorly informative of their actual implementation and efficacy at the individual center level. In this point of view, this represents a real-life study on which has oncology centers’ situation was at the beginning of the pandemic. Surveys seem an excellent tool to perform such analyses because of their quickness and ease of delivery. However, they are so highly heterogeneous: the portrait of the real situation they give is not a masterpiece to hang up on the wall of a museum, but they are a well-done sketch that suffices to understand the picture. Recently, some concerns were raised about the qualitative research of survey questionnaires. In particular, possible limitations include small sample sizes, potential response bias, self-selection bias and potentially inappropriate respondent questions (87). However, qualitative research has the potential to capture individual reactions and feelings without constraints, which is important in extraordinary circumstances such as a health emergency. Furthermore, recent studies such as that of Sneiderman et al. show that comprehensive analyses using qualitative/mixed methods are feasible as well as informative during the pandemic (88).

In conclusion, this study revealed that COVID-19 had a negative impact on cancer care, with deleterious effects felt in all medical, surgical and radiotherapy areas, leading to a reduction in care activities in more than half of cancer centres worldwide. The impact has not been uniform, but has affected all countries regardless of income, reflecting the truly global nature of the pandemic consequences. The individual and rapid response of cancer centres that emerged from this meta-analysis would suggest that the oncology community has already pre-existing strengths in collaboration, advocacy, respect for multidisciplinary teams, and a strong sense of its mission. However, it is imperative that health organisations around the world put measures in place to support professionals, both during the evolution of the current pandemic and in planning for future catastrophic events.

## Data availability statement

The original contributions presented in the study are included in the article/ **Supplementary Material**. Further inquiries can be directed to the corresponding author.

## Author contributions

SDC, NSi, GA, and VR designed the study. VR and NSu did the literature search. NSu did the statistical analyses. All authors wrote the manuscript. All authors had full access to all the data in the study and had final responsibility for the decision to submit for publication. VR and NSu have accessed and verified the data. All authors contributed to the article and approved the submitted version.

## Conflict of interest

The authors declare that the research was conducted in the absence of any commercial or financial relationships that could be construed as a potential conflict of interest.

## Publisher’s note

All claims expressed in this article are solely those of the authors and do not necessarily represent those of their affiliated organizations, or those of the publisher, the editors and the reviewers. Any product that may be evaluated in this article, or claim that may be made by its manufacturer, is not guaranteed or endorsed by the publisher.
