# Supplementary material for: The worldwide impact of COVID-19 on cancer care: A meta-analysis of surveys published after the first wave of the pandemic
Source: Front Oncol. 2022 Sep 29;12:961380. doi: 10.3389/fonc.2022.961380 (PMC9556993; doi:10.3389/fonc.2022.961380)
Supplement: Supplementary file 1 [file DataSheet_1.docx]

Supplementary Material

1. **SEARCH STRING FOR PUBMED**

(COVID-19 OR COVID 19 OR COVID OR novel coronavirus OR n-CoV-2 OR SARS-CoV-2) AND (cancer OR tumor OR neoplasm OR neoplasia) AND (survey OR care OR management)

1. **FULL TEXT ARTICLES EXAMINED AND DECISIONS**

| Abdessater M, Rouprêt M, Misrai V, Matillon X, Gondran-Tellier B, Freton L, Vallée M, Dominique I, Felber M, Khene ZE, Fortier E, Lannes F, Michiels C, Grevez T, Szabla N, Boustany J, Bardet F, Kaulanjan K, Seizilles de Mazancourt E, Ploussard G, Pinar U, Pradere B; Association Française des Urologues en Formation (AFUF). COVID19 pandemic impacts on anxiety of French urologist in training: Outcomes from a national survey. Prog Urol. 2020 Jun-Jul;30(8-9):448-455. doi: 10.1016/j.purol.2020.04.015. Epub 2020 Apr 23. PMID: 32376208; PMCID: PMC7177119. | Wrong outcome |
| --- | --- |
| Abuladze M, Saganelidze K. 1768P Caring for cancer patients in the wake of COVID-19 pandemic in Georgia. Ann Oncol. 2020 Sep;31:S1027. doi: 10.1016/j.annonc.2020.08.1832. Epub 2020 Sep 22. PMCID: PMC7506301. | Wrong population |
| Aeppli S, Eboulet EI, Eisen T, Escudier B, Fischer S, Larkin J, Gruenwald V, McDermott D, Oldenburg J, Omlin A, Porta C, Rini B, Schmidinger M, Sternberg C, Rothermundt C. Impact of COVID-19 pandemic on treatment patterns in metastatic clear cell renal cell carcinoma. ESMO Open. 2020 Jul;5(Suppl 3):e000852. doi: 10.1136/esmoopen-2020-000852. PMID: 32669298; PMCID: PMC7368485. | Wrong study design |
| Alboraie M, Piscoya A, Tran QT, Mendelsohn RB, Butt AS, Lenz L, Alavinejad P, Emara MH, Samlani Z, Altonbary A, Monged A, Lemmers A, Sudovykh I, Ho DQD, Ghazanfar S, Kamau E, Iqbal S, Tan DMY, Liao WC, Vignesh S; “The Global Endo-COVID working group”. The global impact of COVID-19 on gastrointestinal endoscopy units: An international survey of endoscopists. Arab J Gastroenterol. 2020 Sep;21(3):156-161. doi: 10.1016/j.ajg.2020.08.008. Epub 2020 Aug 26. PMID: 32912748; PMCID: PMC7448955. | Wrong study design |
| Alexander M, Jupp J, Chazan G, O'Connor S, Chan A. Global oncology pharmacy response to COVID-19 pandemic: Medication access and safety. J Oncol Pharm Pract. 2020 Jul;26(5):1225-1229. doi: 10.1177/1078155220927450. Epub 2020 May 14. PMID: 32408842. | Wrong population |
| Allevi F, Dionisio A, Baciliero U, Balercia P, Beltramini GA, Bertossi D, Bozzetti A, Califano L, Cascone P, Colombo L, Copelli C, De Ponte FS, De Riu G, Della Monaca M, Fusetti S, Galié M, Giannì AB, Longo F, Mannucci N, Nocini PF, Pelo S, Ramieri G, Sesenna E, Solazzo L, Spinelli G, Tarsitano A, Tartaro G, Valentini V, Verrina G, Biglioli F. Impact of COVID-19 epidemic on maxillofacial surgery in Italy. Br J Oral Maxillofac Surg. 2020 Jul;58(6):692-697. doi: 10.1016/j.bjoms.2020.04.035. Epub 2020 May 3. PMID: 32414539; PMCID: PMC7196423. | Wrong population |
| Altın D, Yalçın İ, Khatib G, Dağgez Keleşoğlu M, Akgöl S, Önder AB, Kahramanoğlu İ, Güvenal T, Topuz S, Demirkıran F. Management of gynecological cancers in the COVID-19 era: a survey from Turkey. J Turk Ger Gynecol Assoc. 2020 Dec 4;21(4):265-271. doi: 10.4274/jtgga.galenos.2020.2020.0071. PMID: 33274616; PMCID: PMC7726458. | Wrong study design |
| Annunziata S, Albano D, Laudicella R, Bauckneht M; Young Committee of the Italian Association of Nuclear Medicine (AIMN). Surveys on COVID-19 in nuclear medicine: what happened and what we learned. Clin Transl Imaging. 2020 Sep 24:1-3. doi: 10.1007/s40336-020-00391-z. Epub ahead of print. PMID: 32989416; PMCID: PMC7512213. | Wrong publication type |
| Archer S, Holch P, Armes J, Calman L, Foster C, Gelcich S, MacLennan SJ, Absolom K. "No turning back" Psycho-oncology in the time of COVID-19: Insights from a survey of UK professionals. Psychooncology. 2020 Sep;29(9):1430-1435. doi: 10.1002/pon.5486. Epub 2020 Aug 7. PMID: 32691451; PMCID: PMC7404944. | Wrong outcome |
| Ashcroft S, Meeking K, Price P. Flash Survey on the Effect of COVID-19 on Radiotherapy Services in the UK - the Benefit of Social Media for Rapid Information Gathering for the Radiotherapy Community and Government. Clin Oncol (R Coll Radiol). 2021 Jan;33(1):e88. doi: 10.1016/j.clon.2020.08.005. Epub 2020 Sep 6. PMID: 32900587; PMCID: PMC7474821. | Wrong publication type |
| Atreya S, Kumar G, Samal J, Bhattacharya M, Banerjee S, Mallick P, Chakraborty D, Gupta S, Sarkar S. Patients'/Caregivers' Perspectives on Telemedicine Service for Advanced Cancer Patients during the COVID-19 Pandemic: An Exploratory Survey. Indian J Palliat Care. 2020 Jun;26(Suppl 1):S40-S44. doi: 10.4103/IJPC.IJPC_145_20. Epub 2020 Jun 30. PMID: 33088085; PMCID: PMC7535001. | Wrong population |
| Ballatore Z, Bastianelli L, Merloni F, Ranallo N, Cantini L, Marcantognini G, Berardi R. Scientia Potentia Est: How the Italian World of Oncology Changes in the COVID-19 Pandemic. JCO Glob Oncol. 2020 Jul;6:1017-1023. doi: 10.1200/GO.20.00209. PMID: 32634067; PMCID: PMC7392780. | Wrong outcome |
| Bao KKH, Cheung KM, Chow JCH, Leung CWL, Tsui W, Lau A, Tse T, Wan S, Kwok M, Wong KH. 1709P Cancer patients' perspectives on the real-world impact of COVID-19 pandemic: A multidisciplinary survey. Ann Oncol. 2020 Sep;31:S1006. doi: 10.1016/j.annonc.2020.08.1773. Epub 2020 Sep 22. PMCID: PMC7506366. | Wrong population |
| Belle A, Barret M, Bernardini D, Tarrerias AL, Bories E, Costil V, Denis B, Gincul R, Karsenti D, Koch S, Laquiere A, Lecomte T, Quentin V, Rahmi G, Robaszkiewicz M, Vaillant E, Vanbiervliet G, Vienne A, Dumeiran F, Gronier O, Chaussade S; French Society of Digestive Endoscopy (Société Française d’Endoscopie Digestive). Impact of the COVID-19 pandemic on gastrointestinal endoscopy activity in France. Endoscopy. 2020 Dec;52(12):1111-1115. doi: 10.1055/a-1201-9618. Epub 2020 Jun 17. PMID: 32557489; PMCID: PMC7724577. | Wrong population |
| Biagioli V, Albanesi B, Belloni S, Piredda A, Caruso R. Living with cancer in the COVID-19 pandemic: An Italian survey on self-isolation at home. Eur J Cancer Care (Engl). 2021 Mar;30(2):e13385. doi: 10.1111/ecc.13385. Epub 2020 Dec 7. PMID: 33289205; PMCID: PMC7883078. | Wrong population |
| Biagioli V, Belloni S, Albanesi B, Piredda A, Caruso R. CN29 SARS-CoV-2 and the perspectives of people living with cancer: The AIIAO survey on the Italian lockdown. Ann Oncol. 2020 Sep;31:S1137–8. doi: 10.1016/j.annonc.2020.08.2137. Epub 2020 Sep 22. PMCID: PMC7506489. | Wrong population |
| Boehm K, Thomas A, Bex A, Black PC, Coburn M, Haferkamp A, Hamdy F, Kaufman RP Jr, Klotz L, Lerner SP, Pushkar D, Ramon J, Rosenzweig B, Tsaur I. Outreach and Influence of Surgical Societies' Recommendations on Minimally Invasive Surgery During the COVID-19 Pandemic-An Anonymized International Urologic Expert Inquiry. Urology. 2020 Nov;145:73-78. doi: 10.1016/j.urology.2020.07.043. Epub 2020 Aug 8. PMID: 32781078; PMCID: PMC7414774. | Wrong population |
| BrintzenhofeSzoc K, Krok-Schoen JI, Pisegna JL, MacKenzie AR, Canin B, Plotkin E, Boehmer LM, Shahrokni A. Survey of cancer care providers' attitude toward care for older adults with cancer during the COVID-19 pandemic. J Geriatr Oncol. 2021 Mar;12(2):196-205. doi: 10.1016/j.jgo.2020.09.028. Epub 2020 Oct 5. PMID: 33144071; PMCID: PMC7534786. | Wrong study design |
| Brody RM, Albergotti WG, Shimunov D, Nicolli E, Patel UA, Harris BN, Bur AM. Changes in head and neck oncologic practice during the COVID-19 pandemic. Head Neck. 2020 Jul;42(7):1448-1453. doi: 10.1002/hed.26233. Epub 2020 Jun 11. PMID: 32357380; PMCID: PMC7267666. | Wrong outcome |
| Büntzel J, Klein M, Keinki C, Walter S, Büntzel J, Hübner J. Oncology services in corona times: a flash interview among German cancer patients and their physicians. J Cancer Res Clin Oncol. 2020 Oct;146(10):2713-2715. doi: 10.1007/s00432-020-03249-z. Epub 2020 May 15. PMID: 32415341; PMCID: PMC7226710. | Wrong outcome |
| Büssing A, Hübner J, Walter S, Gießler W, Büntzel J. Tumor Patients´ Perceived Changes of Specific Attitudes, Perceptions, and Behaviors Due to the COVID-19 Pandemic and Its Relation to Reduced Wellbeing. Front Psychiatry. 2020 Oct 9;11:574314. doi: 10.3389/fpsyt.2020.574314. PMID: 33192703; PMCID: PMC7581913. | Wrong population |
| Casanova M, Pagani Bagliacca E, Silva M, Patriarca C, Veneroni L, Clerici CA, Spreafico F, Luksch R, Terenziani M, Meazza C, Podda M, Biassoni V, Schiavello E, Chiaravalli S, Puma N, Bergamaschi L, Gattuso G, Sironi G, Massimino M, Ferrari A. How young patients with cancer perceive the COVID-19 (coronavirus) epidemic in Milan, Italy: Is there room for other fears? Pediatr Blood Cancer. 2020 Jul;67(7):e28318. doi: 10.1002/pbc.28318. Epub 2020 Apr 22. PMID: 32240567. | Wrong population |
| Catania C, Spitaleri G, Del Signore E, Attili I, Radice D, Stati V, Gianoncelli L, Morganti S, de Marinis F. Fears and Perception of the Impact of COVID-19 on Patients With Lung Cancer: A Mono-Institutional Survey. Front Oncol. 2020 Oct 14;10:584612. doi: 10.3389/fonc.2020.584612. PMID: 33163413; PMCID: PMC7591454. | Wrong population |
| Cavalcante FP, Novita GG, Millen EC, Zerwes FP, de Oliveira VM, Sousa ALL, Freitas Junior R. Management of early breast cancer during the COVID-19 pandemic in Brazil. Breast Cancer Res Treat. 2020 Nov;184(2):637-647. doi: 10.1007/s10549-020-05877-y. Epub 2020 Aug 16. PMID: 32803637; PMCID: PMC7429139. | Wrong study design |
| Chan A, Ashbury F, Fitch MI, Koczwara B, Chan RJ; MASCC Survivorship Study Group. Cancer survivorship care during COVID-19-perspectives and recommendations from the MASCC survivorship study group. Support Care Cancer. 2020 Aug;28(8):3485-3488. doi: 10.1007/s00520-020-05544-4. PMID: 32451702; PMCID: PMC7247777. | Wrong publication type |
| Chaurasia AR, Page BR, Walker AJ, Salerno K, Camphausen K, Kwok Y, Bajaj GK, Ambrocio D, Erickson D. Lessons to Learn From a Successful Virtual Mock Oral Examination Pilot Experience. Adv Radiat Oncol. 2021 Jan-Feb;6(1):100534. doi: 10.1016/j.adro.2020.07.011. Epub 2020 Aug 8. PMID: 32838071; PMCID: PMC7414305. | Wrong outcome |
| Chin KE, Kwon D, Gan Q, Ramalingam PX, Wistuba II, Prieto VG, Aung PP. Transition From a Standard to a Hybrid On-Site and Remote Anatomic Pathology Training Model During the Coronavirus Disease 2019 (COVID-19) Pandemic. Arch Pathol Lab Med. 2021 Jan 1;145(1):22-31. doi: 10.5858/arpa.2020-0467-SA. PMID: 32937659. | Wrong outcome |
| Chu KM, Smith M, Steyn E, Goldberg P, Bougard H, Buccimazza I. Changes in surgical practice in 85 South African hospitals during COVID-19 hard lockdown. S Afr Med J. 2020 Jul 28;110(9):916-919. PMID: 32880278. | Wrong population |
| Darcourt JG, Aparicio K, Dorsey PM, Ensor JE, Zsigmond EM, Wong ST, Ezeana CF, Puppala M, Heyne KE, Geyer CE, Phillips RA, Schwartz RL, Chang JC. Analysis of the Implementation of Telehealth Visits for Care of Patients With Cancer in Houston During the COVID-19 Pandemic. JCO Oncol Pract. 2021 Jan;17(1):e36-e43. doi: 10.1200/OP.20.00572. Epub 2020 Oct 7. PMID: 33026951; PMCID: PMC8202056. | Wrong study design |
| Darlington AE, Morgan JE, Wagland R, Sodergren SC, Culliford D, Gamble A, Phillips B. COVID-19 and children with cancer: Parents' experiences, anxieties and support needs. Pediatr Blood Cancer. 2021 Feb;68(2):e28790. doi: 10.1002/pbc.28790. Epub 2020 Nov 21. PMID: 33219739; PMCID: PMC7744834. | Wrong population |
| de Joode K, Dumoulin DW, Engelen V, Bloemendal HJ, Verheij M, van Laarhoven HWM, Dingemans IH, Dingemans AC, van der Veldt AAM. Impact of the coronavirus disease 2019 pandemic on cancer treatment: the patients' perspective. Eur J Cancer. 2020 Sep;136:132-139. doi: 10.1016/j.ejca.2020.06.019. Epub 2020 Jul 4. PMID: 32683273; PMCID: PMC7334940. | Wrong population |
| Desideri I, Francolini G, Ciccone LP, Stocchi G, Salvestrini V, Aquilano M, Greto D, Bonomo P, Meattini I, Scotti V, Scoccianti S, Simontacchi G, Livi L. Impact of COVID-19 on patient-doctor interaction in a complex radiation therapy facility. Support Care Cancer. 2021 Jun;29(6):2931-2937. doi: 10.1007/s00520-020-05793-3. Epub 2020 Oct 2. Erratum in: Support Care Cancer. 2022 Feb;30(2):1891-1892. PMID: 33006676; PMCID: PMC7531068. | Wrong population |
| Dhont J, Di Tella M, Dubois L, Aznar M, Petit S, Spałek M, Boldrini L, Franco P, Bertholet J. Conducting research in Radiation Oncology remotely during the COVID-19 pandemic: Coping with isolation. Clin Transl Radiat Oncol. 2020 Jun 18;24:53-59. doi: 10.1016/j.ctro.2020.06.006. PMID: 32632379; PMCID: PMC7299875. | Wrong outcome |
| Domenig P, Booher J, Goldman B, Greenlee J, Sircus S, Boura JA, Chuba P. Management of Prostate Cancer During COVID-19 Pandemic: Perspective From Urologists and Radiation Oncologists in COVID Dense Metro Detroit. Cureus. 2020 Aug 10;12(8):e9648. doi: 10.7759/cureus.9648. PMID: 32923248; PMCID: PMC7480779. | Wrong study design |
| Dubin JM, Wyant WA, Balaji NC, Ong WL, Kettache RH, Haffaf M, Zouari S, Santillan D, Autrán Gómez AM, Sadeghi-Nejad H, Loeb S, Borin JF, Gomez Rivas J, Grummet J, Ramasamy R, Teoh JYC. Telemedicine Usage Among Urologists During the COVID-19 Pandemic: Cross-Sectional Study. J Med Internet Res. 2020 Nov 5;22(11):e21875. doi: 10.2196/21875. PMID: 33031047; PMCID: PMC7647472. | Wrong study design |
| Erdem D, Karaman I. Awareness and perceptions related to COVID-19 among cancer patients: A survey in oncology department. Eur J Cancer Care (Engl). 2020 Nov;29(6):e13309. doi: 10.1111/ecc.13309. Epub 2020 Sep 18. PMID: 32945043; PMCID: PMC7536946. | Wrong population |
| Erica T. Warner, Emily Restrepo, Christine Benjamin, Ricki Fairley, Laura Roudebush, Leah Eshraghi, Crystal Hertz, Simo Du and Laura Carfang, Abstract S11-02: Patient-reported impact of the COVID-19 pandemic on breast cancer screening, diagnosis, and treatment: A national survey, Clin Cancer Res September 15 2020 (26) (18 Supplement) S11-02; DOI: 10.1158/1557-3265.COVID-19-S11-02 | Wrong population |
| Fabi A, Pugliese P, Falbo PT, Corsi D, Fabbri MA, Vincenzi B, Bria E, Angelini F, Bonucci A, Pellegrino A, Falcicchio C, Caruso A, Giacomelli L, Mirisola V, Papa S, Cognetti F, Ciliberto G, Perrone M. The Experience of Oncology Healthcare Providers in the Central Italy during the COVID-19 Lockdown. Cancers (Basel). 2020 Oct 18;12(10):3031. doi: 10.3390/cancers12103031. PMID: 33081044; PMCID: PMC7603147. | Wrong outcome |
| Falcone R, Grani G, Ramundo V, Melcarne R, Giacomelli L, Filetti S, Durante C. Cancer Care During COVID-19 Era: The Quality of Life of Patients With Thyroid Malignancies. Front Oncol. 2020 Jun 23;10:1128. doi: 10.3389/fonc.2020.01128. PMID: 32714873; PMCID: PMC7344223. | Wrong population |
| Fedele P, Ferro A, Sanna V, La Verde N, Paris I, Chiari R. Exploring metastatic breast cancer treatment changes during COVID-19 pandemic. J Chemother. 2021 Jul;33(4):263-268. doi: 10.1080/1120009X.2020.1829328. Epub 2020 Oct 13. PMID: 33047649. | Wrong outcome |
| Fero KE, Weinberger JM, Lerman S, Bergman J. Perceived Impact of Urologic Surgery Training Program Modifications due to COVID-19 in the United States. Urology. 2020 Sep;143:62-67. doi: 10.1016/j.urology.2020.05.051. Epub 2020 Jun 6. PMID: 32512110; PMCID: PMC7274971. | Wrong outcome |
| Fleury ME, Farner AM, Unger JM. Association of the COVID-19 Outbreak With Patient Willingness to Enroll in Cancer Clinical Trials. JAMA Oncol. 2021 Jan 1;7(1):131-132. doi: 10.1001/jamaoncol.2020.5748. PMID: 33180102; PMCID: PMC7662493. | Wrong outcome |
| Foà R, Bonifacio M, Chiaretti S, Curti A, Candoni A, Fava C, Ciccone M, Pizzolo G, Ferrara F. Philadelphia-positive acute lymphoblastic leukaemia (ALL) in Italy during the COVID-19 pandemic: a Campus ALL study. Br J Haematol. 2020 Jul;190(1):e3-e5. doi: 10.1111/bjh.16758. Epub 2020 Jun 14. PMID: 32368790; PMCID: PMC7267647. | Wrong study design |
| Frey MK, Ellis AE, Zeligs K, Chapman-Davis E, Thomas C, Christos PJ, Kolev V, Prasad-Hayes M, Cohen S, Holcomb K, Blank SV. Impact of the coronavirus disease 2019 pandemic on the quality of life for women with ovarian cancer. Am J Obstet Gynecol. 2020 Nov;223(5):725.e1-725.e9. doi: 10.1016/j.ajog.2020.06.049. Epub 2020 Jun 26. PMID: 32598911; PMCID: PMC7318934. | Wrong population |
| Fu R, Wu L, Zhang C, Chu Q, Hu J, Lin G, Yang L, Li JS, Yang XN, Yang JJ, Zhou Q, Wu YL, Zhong WZ. Real-World Scenario of Patients With Lung Cancer Amid the Coronavirus Disease 2019 Pandemic in the People's Republic of China. JTO Clin Res Rep. 2020 Sep;1(3):100053. doi: 10.1016/j.jtocrr.2020.100053. Epub 2020 May 20. PMID: 32929416; PMCID: PMC7239012. | Wrong population |
| Gebbia V, Piazza D, Valerio MR, Borsellino N, Firenze A. Patients With Cancer and COVID-19: A WhatsApp Messenger-Based Survey of Patients' Queries, Needs, Fears, and Actions Taken. JCO Glob Oncol. 2020 May;6:722-729. doi: 10.1200/GO.20.00118. PMID: 32412811; PMCID: PMC7271316. | Wrong population |
| Gerber DE, Sheffield TY, Beg MS, Williams EL, Clark VL, Xie Y, Holbein MEB, Skinner CS, Lee SJC. Experience, Perceptions, and Recommendations Concerning COVID-19-Related Clinical Research Adjustments. J Natl Compr Canc Netw. 2020 Oct 7;19(5):505-512. doi: 10.6004/jnccn.2020.7643. PMID: 33027755; PMCID: PMC8173586. | Wrong outcome |
| Gharzai LA, Resnicow K, An LC, Jagsi R. Perspectives on Oncology-Specific Language During the Coronavirus Disease 2019 Pandemic: A Qualitative Study. JAMA Oncol. 2020 Sep 1;6(9):1424-1428. doi: 10.1001/jamaoncol.2020.2980. PMID: 32761102; PMCID: PMC7411932. | Wrong study design |
| Ghosh J, Ganguly S, Mondal D, Pandey P, Dabkara D, Biswas B. Perspective of Oncology Patients During COVID-19 Pandemic: A Prospective Observational Study From India. JCO Glob Oncol. 2020 Jun;6:844-851. doi: 10.1200/GO.20.00172. PMID: 32552110; PMCID: PMC7328097. | Wrong population |
| Ghosh N, Tirpack A, Chan KK, Bass AR. Impact of COVID-19 on patients with rheumatic complications of cancer immunotherapy: results of a registry survey. J Immunother Cancer. 2020 Oct;8(2):e001550. doi: 10.1136/jitc-2020-001550. PMID: 33067320; PMCID: PMC7569707. | Wrong population |
| Giles RH, Baugh E, Cardoso F, Filicevas A, Fox J, Oliver K, Reid F, Spiegel A, Warwick L, MacKay C. 1759P Cancer care during COVID-19: Data from 157 patient organisations. Ann Oncol. 2020 Sep;31:S1024. doi: 10.1016/j.annonc.2020.08.1823. Epub 2020 Sep 22. PMCID: PMC7506303. | Wrong outcome |
| Givi B, Moore MG, Bewley AF, Coffey CS, Cohen MA, Hessel AC, Jalisi S, Kang S, Newman JG, Puscas L, Shindo M, Shuman A, Thakkar P, Weed DT, Chalian A. Advanced head and neck surgery training during the COVID-19 pandemic. Head Neck. 2020 Jul;42(7):1411-1417. doi: 10.1002/hed.26252. Epub 2020 May 8. PMID: 32383550. | Wrong outcome |
| Gravas S, Fournier G, Oya M, Summerton D, Scarpa RM, Chlosta P, Gkialas I, Xie LP, Rasyid N, Bolton D, Gomez R, Klotz L, Kulkarni S, Tanguay S, de la Rosette J; SIU Board of Directors. Prioritising Urological Surgery in the COVID-19 Era: A Global Reflection on Guidelines. Eur Urol Focus. 2020 Sep 15;6(5):1104-1110. doi: 10.1016/j.euf.2020.06.006. Epub 2020 Jun 15. PMID: 32571743; PMCID: PMC7294295. | Wrong study design |
| Greco F, Altieri VM, Esperto F, Mirone V, Scarpa RM. Impact of COVID-19 Pandemic on Health-Related Quality of Life in Uro-oncologic Patients: What Should We Wait For? Clin Genitourin Cancer. 2021 Apr;19(2):e63-e68. doi: 10.1016/j.clgc.2020.07.008. Epub 2020 Jul 17. PMID: 32863188; PMCID: PMC7366083. | Wrong population |
| Grova MM, Donohue SJ, Meyers MO, Kim HJ, Ollila DW. Direct Comparison of In-Person Versus Virtual Interviews for Complex General Surgical Oncology Fellowship in the COVID-19 Era. Ann Surg Oncol. 2021 Apr;28(4):1908-1915. doi: 10.1245/s10434-020-09398-2. Epub 2020 Nov 26. PMID: 33244739; PMCID: PMC7690846. | Wrong outcome |
| Gultekin M, Ak S, Ayhan A, Strojna A, Pletnev A, Fagotti A, Perrone AM, Erzeneoglu BE, Temiz BE, Lemley B, Soyak B, Hughes C, Cibula D, Haidopoulos D, Brennan D, Cola E, van der Steen-Banasik E, Urkmez E, Akilli H, Zapardiel I, Tóth I, Sehouli J, Zalewski K, Bahremand K, Chiva L, Mirza MR, Papageorgiou M, Zoltan N, Adámková P, Morice P, Garrido-Mallach S, Akgor U, Theodoulidis V, Arik Z, Steffensen KD, Fotopoulou C. Perspectives, fears and expectations of patients with gynaecological cancers during the COVID-19 pandemic: A Pan-European study of the European Network of Gynaecological Cancer Advocacy Groups (ENGAGe). Cancer Med. 2021 Jan;10(1):208-219. doi: 10.1002/cam4.3605. Epub 2020 Nov 18. PMID: 33205595; PMCID: PMC7753798. | Wrong population |
| Guo T, Kiong KL, Yao CMKL, Windon M, Zebda D, Jozaghi Y, Zhao X, Hessel AC, Hanna EY. Impact of the COVID-19 pandemic on Otolaryngology trainee education. Head Neck. 2020 Oct;42(10):2782-2790. doi: 10.1002/hed.26368. Epub 2020 Jul 15. PMID: 32666664; PMCID: PMC7405272. | Wrong outcome |
| Guven DC, Sahin TK, Aktepe OH, Yildirim HC, Aksoy S, Kilickap S. Perspectives, Knowledge, and Fears of Cancer Patients About COVID-19. Front Oncol. 2020 Aug 28;10:1553. doi: 10.3389/fonc.2020.01553. PMID: 33014800; PMCID: PMC7493662. | Wrong population |
| Haase KR, Kain D, Merchant S, Booth C, Koven R, Brundage M, Galica J. Older survivors of cancer in the COVID-19 pandemic: Reflections and recommendations for future care. J Geriatr Oncol. 2021 Apr;12(3):461-466. doi: 10.1016/j.jgo.2020.11.009. Epub 2020 Dec 3. PMID: 33303410; PMCID: PMC7713572. | Wrong population |
| Hajjaji N, Lakhdar S, Kaczmarek E, Bellier C, Bécourt S, Broyelle A, Girard EI, Giscard S, Lartigau E. 1708P Online survey on SARS-CoV-2 infections in cancer patients during a nationwide lockdown in France. Ann Oncol. 2020 Sep;31:S1005. doi: 10.1016/j.annonc.2020.08.1772. Epub 2020 Sep 22. PMCID: PMC7506442. | Wrong population |
| Heinze A, Umari P, Basulto-Martínez M, Suárez-Ibarrola R, Liatsikos E, Rassweiler J, Guven S, Gözen AS. Impact of COVID-19 on Clinical and Academic Urological Practice: A Survey from European Association of Urology Section of Uro-technology. Eur Urol Open Sci. 2020 Oct;21:22-28. doi: 10.1016/j.euros.2020.08.001. Epub 2020 Aug 17. PMID: 33123688; PMCID: PMC7430276. | Wrong outcome |
| Helm, Erin E. ; Kempski, Katelyn A. ; Galantino, Mary Lou A. Effect of Disrupted Rehabilitation Services on Distress and Quality of Life in Breast Cancer Survivors During the COVID-19 Pandemic, Rehabilitation Oncology: October 2020 - Volume 38 - Issue 4 - p 153-158 doi: 10.1097/01.REO.0000000000000233 | Wrong population |
| Helton G, Wolfe J, Snaman JM. Definitely Mixed Feelings: The Effect of COVID-19 on Bereavement in Parents of Children Who Died of Cancer. J Pain Symptom Manage. 2020 Nov;60(5):e15-e20. doi: 10.1016/j.jpainsymman.2020.08.035. Epub 2020 Sep 2. PMID: 32889042; PMCID: PMC7467087. | Wrong population |
| Hilmi M, Boilève A, Ducousso A, Michalet M, Turpin A, Neuzillet C, Naoun N. Professional and Psychological Impacts of the COVID-19 Pandemic on Oncology Residents: A National Survey. JCO Glob Oncol. 2020 Oct;6:1674-1683. doi: 10.1200/GO.20.00376. PMID: 33151771; PMCID: PMC7713519. | Wrong outcome |
| Hoffman KE, Garner D, Koong AC, Woodward WA. Understanding the Intersection of Working from Home and Burnout to Optimize Post-COVID19 Work Arrangements in Radiation Oncology. Int J Radiat Oncol Biol Phys. 2020 Oct 1;108(2):370-373. doi: 10.1016/j.ijrobp.2020.06.062. PMID: 32890515; PMCID: PMC7462773. | Wrong outcome |
| Islam JY, Camacho-Rivera M, Vidot DC. Examining COVID-19 Preventive Behaviors among Cancer Survivors in the United States: An Analysis of the COVID-19 Impact Survey. Cancer Epidemiol Biomarkers Prev. 2020 Dec;29(12):2583-2590. doi: 10.1158/1055-9965.EPI-20-0801. Epub 2020 Sep 25. PMID: 32978173; PMCID: PMC7871461. | Wrong population |
| Jeppesen SS, Bentsen KK, Jørgensen TL, Holm HS, Holst-Christensen L, Tarpgaard LS, Dahlrot RH, Eckhoff L. Quality of life in patients with cancer during the COVID-19 pandemic - a Danish cross-sectional study (COPICADS). Acta Oncol. 2021 Jan;60(1):4-12. doi: 10.1080/0284186X.2020.1830169. Epub 2020 Oct 8. PMID: 33031010. | Wrong population |
| Joly F, Leconte A, Grellard JM, Lequesne J, Binarelli G, Lange M, Tron L, Gernier F, Rieux C, Fernette M, Bastien E, Morel A, Legrand B, Richard D, Faveyrial A, Travers R, Pépin LF, Rigal O, Jardin F, Clarisse B. LBA69 Impact of the COVID-19 pandemic on management of medical cancer treatments and psychological consequence for the patients. Ann Oncol. 2020 Sep;31:S1200. doi: 10.1016/j.annonc.2020.08.2310. Epub 2020 Sep 22. PMCID: PMC7506429. | Wrong population |
| Juanjuan L, Santa-Maria CA, Hongfang F, Lingcheng W, Pengcheng Z, Yuanbing X, Yuyan T, Zhongchun L, Bo D, Meng L, Qingfeng Y, Feng Y, Yi T, Shengrong S, Xingrui L, Chuang C. Patient-reported Outcomes of Patients With Breast Cancer During the COVID-19 Outbreak in the Epicenter of China: A Cross-sectional Survey Study. Clin Breast Cancer. 2020 Oct;20(5):e651-e662. doi: 10.1016/j.clbc.2020.06.003. Epub 2020 Jun 7. PMID: 32709505; PMCID: PMC7275993. | Wrong population |
| Juanjuan L, Santa-Maria CA, Hongfang F, Lingcheng W, Pengcheng Z, Yuanbing X, Yuyan T, Zhongchun L, Bo D, Meng L, Qingfeng Y, Feng Y, Yi T, Shengrong S, Xingrui L, Chuang C. Patient-reported Outcomes of Patients With Breast Cancer During the COVID-19 Outbreak in the Epicenter of China: A Cross-sectional Survey Study. Clin Breast Cancer. 2020 Oct;20(5):e651-e662. doi: 10.1016/j.clbc.2020.06.003. Epub 2020 Jun 7. PMID: 32709505; PMCID: PMC7275993. | Duplicate |
| K Moran H, Brooks JV, Spoozak L. Undergoing active treatment for gynecologic cancer during COVID-19: A qualitative study of the impact on healthcare and social support. Gynecol Oncol Rep. 2020 Nov;34:100659. doi: 10.1016/j.gore.2020.100659. Epub 2020 Oct 21. PMID: 33106774; PMCID: PMC7577250. | Wrong population |
| Kent DG, Knapp DJHF, Kannan N. Survey Says: "COVID-19 Lockdown Hits Young Faculty and Clinical Trials". Stem Cell Reports. 2020 Jul 14;15(1):1-5. doi: 10.1016/j.stemcr.2020.06.010. Epub 2020 Jun 22. PMID: 32574555; PMCID: PMC7307517. | Wrong outcome |
| Košir U, Loades M, Wild J, Wiedemann M, Krajnc A, Roškar S, Bowes L. The impact of COVID-19 on the cancer care of adolescents and young adults and their well-being: Results from an online survey conducted in the early stages of the pandemic. Cancer. 2020 Oct 1;126(19):4414-4422. doi: 10.1002/cncr.33098. Epub 2020 Jul 22. PMID: 32697342; PMCID: PMC7405129. | Wrong population |
| Kowalski LP, Imamura R, Castro Junior G, Marta GN, Chaves ALF, Matos LL, Bento RF. Effect of the COVID-19 Pandemic on the Activity of Physicians Working in the Areas of Head and Neck Surgery and Otorhinolaryngology. Int Arch Otorhinolaryngol. 2020 Jul;24(3):e258-e266. doi: 10.1055/s-0040-1712169. Epub 2020 May 22. PMID: 32754234; PMCID: PMC7394652. | Wrong population |
| Krok-Schoen JL, Pisegna JL, BrintzenhofeSzoc K, MacKenzie AR, Canin B, Plotkin E, Boehmer LM, Shahrokni A. Experiences of healthcare providers of older adults with cancer during the COVID-19 pandemic. J Geriatr Oncol. 2021 Mar;12(2):190-195. doi: 10.1016/j.jgo.2020.09.021. Epub 2020 Sep 18. PMID: 32978104; PMCID: PMC7500913. | Wrong population |
| Krug S, Garbe J, König S, Ungewiss H, Michl P, Rinke A, Schrader J. Professional Assessment of the Impact of COVID-19 on Handling NET Patients. J Clin Med. 2020 Nov 11;9(11):3633. doi: 10.3390/jcm9113633. PMID: 33187393; PMCID: PMC7696769. | Wrong study design |
| Layfield E, Triantafillou V, Prasad A, Deng J, Shanti RM, Newman JG, Rajasekaran K. Telemedicine for head and neck ambulatory visits during COVID-19: Evaluating usability and patient satisfaction. Head Neck. 2020 Jul;42(7):1681-1689. doi: 10.1002/hed.26285. Epub 2020 Jun 1. PMID: 32476228; PMCID: PMC7300847. | Wrong population |
| Lou E, Teoh D, Brown K, Blaes A, Holtan SG, Jewett P, Parsons H, Mburu EW, Thomaier L, Hui JYC, Nelson HH, Vogel RI. Perspectives of Cancer Patients and Their Health during the COVID-19 Pandemic. medRxiv [Preprint]. 2020 May 5:2020.04.30.20086652. doi: 10.1101/2020.04.30.20086652. Update in: PLoS One. 2020 Oct 30;15(10):e0241741. PMID: 32511661; PMCID: PMC7277017. | Wrong population |
| Luis Pendola G, Elizalde R, Vargas PS, Mallarino JC, González E, Parada J, Camus M, Schwartz R, Bargalló E, Freitas R, Costa MM, de Oliveira VM, Escobar P, Oller M, Viaña LF, Bambino AJ, Sarria G, Terrier F, Corrales R, Sanabria V, Agostini JCR, Chacón GV, Pérez VM, Avilés V, Galarreta J, Laviña G, Fuentes JP, de Castellanos LB, Osorio BA, Castillo H, Figueroa C. Management of non-invasive tumours, benign tumours and breast cancer during the COVID-19 pandemic: recommendations based on a Latin American survey. Ecancermedicalscience. 2020 Oct 6;14:1115. doi: 10.3332/ecancer.2020.1115. PMID: 33209106; PMCID: PMC7652542. | Wrong study design |
| Magno S, Linardos M, Carnevale S, Dilucca M, Di Leone A, Terribile DA, Franceschini G, Masetti R. The impact of the COVID-19 pandemic on breast cancer patients awaiting surgery: Observational survey in an Italian University hospital. Breast J. 2020 Aug;26(8):1597-1602. doi: 10.1111/tbj.13889. Epub 2020 Jul 17. PMID: 32677117; PMCID: PMC7404646. | Wrong population |
| Mahadev S, Aroniadis OC, Barraza LH, Agarunov E, Smith MS, Goodman AJ, Benias PC, Buscaglia JM, Gross SA, Kasmin F, Cohen J, Carr-Locke DL, Greenwald D, Mendelsohn R, Sethi A, Gonda TA; (on behalf of NYSGE research committee). Gastrointestinal endoscopy during the coronavirus pandemic in the New York area: results from a multi-institutional survey. Endosc Int Open. 2020 Dec;8(12):E1865-E1871. doi: 10.1055/a-1264-7599. Epub 2020 Nov 27. PMID: 33269322; PMCID: PMC7695511. | Wrong study design |
| Maida M, Sferrazza S, Savarino E, Ricciardiello L, Repici A, Morisco F, Furnari M, Fuccio L, Morreale GC, Vitello A, Burra P, Marchi S, Annibale B, Benedetti A, Alvaro D, Ianiro G; Italian Society of Gastroenterology (SIGE). Impact of the COVID-19 pandemic on Gastroenterology Divisions in Italy: A national survey. Dig Liver Dis. 2020 Aug;52(8):808-815. doi: 10.1016/j.dld.2020.05.017. Epub 2020 May 16. PMID: 32425733; PMCID: PMC7229963. | Wrong population |
| Marcasciano M, Kaciulyte J, Mori FLR, Lo Torto F, Barellini L, Loreti A, Fanelli B, De Vita R, Redi U, Marcasciano F, Di Cesare F, Dal Prà G, Conversi A, Elia L, Montemari G, Vaia N, Bernini M, Sordi S, Luridiana G, D'Ermo G, Monti M, De Luca A, Ricci F, Mazzocchi M, Gentilucci M, Greco M, Losco L, Valdatta LA, Raposio E, Giudice G, Maruccia M, Di Benedetto G, Cigna E, Casella D, Ribuffo D. Breast surgeons updating on the thresholds of COVID-19 era: results of a multicenter collaborative study evaluating the role of online videos and multimedia sources on breast surgeons education and training. Eur Rev Med Pharmacol Sci. 2020 Jul;24(14):7845-7854. doi: 10.26355/eurrev_202007_22289. PMID: 32744712. | Wrong outcome |
| Mason SE, Scott AJ, Markar SR, Clarke JM, Martin G, Winter Beatty J, Sounderajah V, Yalamanchili S, Denning M, Arulampalam T, Kinross JM; PanSurg Collaborative. Insights from a global snapshot of the change in elective colorectal practice due to the COVID-19 pandemic. PLoS One. 2020 Oct 8;15(10):e0240397. doi: 10.1371/journal.pone.0240397. PMID: 33031464; PMCID: PMC7544024. | Wrong study design |
| Matuschek C, Fischer JC, Combs SE, Fietkau R, Corradini S, Zänker K, Bölke E, Djiepmo-Njanang FJ, Tamaskovics B, Fischer JE, Stuschke M, Pöttgen C, Förster R, Zwahlen DR, Papachristofilou A, Ganswindt U, Pelka R, Schneider EM, Feldt T, Jensen BEO, Häussinger D, Knoefel WT, Kindgen-Milles D, Pedoto A, Grebe O, van Griensven M, Budach W, Haussmann J. Measures of infection prevention and incidence of SARS-CoV-2 infections in cancer patients undergoing radiotherapy in Germany, Austria and Switzerland. Strahlenther Onkol. 2020 Dec;196(12):1068-1079. doi: 10.1007/s00066-020-01681-1. Epub 2020 Sep 10. PMID: 32914236; PMCID: PMC7483062. | Wrong study design |
| Mian BM, Siddiqui S, Ahmad AE. Management of urologic cancers during the pandemic and potential impact of treatment deferrals on outcomes. Urol Oncol. 2021 May;39(5):258-267. doi: 10.1016/j.urolonc.2020.10.013. Epub 2020 Oct 28. PMID: 33129674; PMCID: PMC7598541. | Wrong study design |
| Miaskowski C, Paul SM, Snowberg K, Abbott M, Borno H, Chang S, Chen LM, Cohen B, Cooper BA, Hammer MJ, Kenfield SA, Laffan A, Levine JD, Pozzar R, Tsai KK, Van Blarigan EL, Van Loon K. Oncology patients' perceptions of and experiences with COVID-19. Support Care Cancer. 2021 Apr;29(4):1941-1950. doi: 10.1007/s00520-020-05684-7. Epub 2020 Aug 18. PMID: 32809060; PMCID: PMC7431899. | Wrong population |
| Miaskowski C, Paul SM, Snowberg K, Abbott M, Borno H, Chang S, Chen LM, Cohen B, Hammer MJ, Kenfield SA, Kober KM, Levine JD, Pozzar R, Rhoads KF, Van Blarigan EL, Van Loon K. Stress and Symptom Burden in Oncology Patients During the COVID-19 Pandemic. J Pain Symptom Manage. 2020 Nov;60(5):e25-e34. doi: 10.1016/j.jpainsymman.2020.08.037. Epub 2020 Sep 2. PMID: 32889039; PMCID: PMC7462969. | Wrong population |
| Milone M, Carrano FM, Letić E, Shamiyeh A, Forgione A, Eom BW, Müller-Stich BP, Ponz CB, Kontovounisios C, Preda D, Ignjatovic D, Cassinotti E, Yiannakopoulou E, Theodoropoulos G, Faria G, Morelli L, Gorter-Stam M, Markar S, Arulampalam T, Velthoven T, Antoniou SA, Francis NK. Surgical challenges and research priorities in the era of the COVID-19 pandemic: EAES membership survey. Surg Endosc. 2020 Oct;34(10):4225-4232. doi: 10.1007/s00464-020-07835-7. Epub 2020 Aug 4. PMID: 32749615; PMCID: PMC7402075. | Wrong population |
| Mitra M, Basu M. A Study on Challenges to Health Care Delivery Faced by Cancer Patients in India During the COVID-19 Pandemic. J Prim Care Community Health. 2020 Jan-Dec;11:2150132720942705. doi: 10.1177/2150132720942705. PMID: 32830619; PMCID: PMC7448263. | Wrong population |
| Mitsuboshi S, Yoshino M, Hosokawa H, Isobe H, Kobayashi K. Use of personal protective equipment while admixing antineoplastic drugs during the COVID-19 pandemic era: Questionnaire survey in Niigata, Japan. J Oncol Pharm Pract. 2020 Sep;26(6):1553-1554. doi: 10.1177/1078155220932342. Epub 2020 May 29. PMID: 32469683. | Wrong study design |
| Musche V, Bäuerle A, Steinbach J, Schweda A, Hetkamp M, Weismüller B, Kohler H, Beckmann M, Herrmann K, Tewes M, Schadendorf D, Skoda EM, Teufel M. COVID-19-Related Fear and Health-Related Safety Behavior in Oncological Patients. Front Psychol. 2020 Aug 5;11:1984. doi: 10.3389/fpsyg.2020.01984. PMID: 32903780; PMCID: PMC7438892. | Wrong population |
| Nappi L, Ottaviano M, Rescigno P, Tortora M, Banna GL, Baciarello G, Basso U, Canil C, Cavo A, Cossu Rocca M, Czaykowski P, De Giorgi U, Garcia Del Muro X, Di Napoli M, Fornarini G, Gietema JA, Heng DYC, Hotte SJ, Kollmannsberger C, Maruzzo M, Messina C, Morelli F, Mulder S, Nichols C, Nolè F, Oing C, Sava T, Secondino S, Simone G, Soulieres D, Vincenzi B, Zucali PA, De Placido S, Palmieri G; Italian Germ Cell Cancer Group (IGG); ERN-EURACAN Domain G3; Genitourinary Medical Oncologists of Canada (GUMOC). Management of Germ Cell Tumors During the Outbreak of the Novel Coronavirus Disease-19 Pandemic: A Survey of International Expertise Centers. Oncologist. 2020 Oct;25(10):e1509-e1515. doi: 10.1634/theoncologist.2020-0420. Epub 2020 Sep 18. PMID: 32735386; PMCID: PMC7543332. | Wrong study design |
| Neeman E, Kolevska T, Reed M et al, Abstract S06-03: Cancer care telehealth utilization rates and provider attitudes in the wake of the novel coronavirus pandemic: The Kaiser Permanente Northern California experience, Clinical cancer research, September 2020 Volume 26, Issue 18 Supplement | Wrong outcome |
| Ng KYY, Zhou S, Tan SH, Ishak NDB, Goh ZZS, Chua ZY, Chia JMX, Chew EL, Shwe T, Mok JKY, Leong SS, Lo JSY, Ang ZLT, Leow JL, Lam CWJ, Kwek JW, Dent R, Tuan J, Lim ST, Hwang WYK, Griva K, Ngeow J. Understanding the Psychological Impact of COVID-19 Pandemic on Patients With Cancer, Their Caregivers, and Health Care Workers in Singapore. JCO Glob Oncol. 2020 Oct;6:1494-1509. doi: 10.1200/GO.20.00374. PMID: 33017179; PMCID: PMC7640379. | Wrong outcome |
| Nicholson P, Ali FR, Patalay R, Craythorne E, Mallipeddi R. Patient perceptions of Mohs micrographic surgery during the COVID-19 pandemic and lessons for the next outbreak. Clin Exp Dermatol. 2021 Jan;46(1):179-180. doi: 10.1111/ced.14423. Epub 2020 Sep 12. PMID: 32803789; PMCID: PMC7460918. | Wrong study design |
| Nindrea RD, Sari NP, Harahap WA, Haryono SJ, Kusnanto H, Dwiprahasto I, Lazuardi L, Aryandono T. Survey data of COVID-19 awareness, knowledge, preparedness and related behaviors among breast cancer patients in Indonesia. Data Brief. 2020 Oct;32:106145. doi: 10.1016/j.dib.2020.106145. Epub 2020 Aug 8. PMID: 32835041; PMCID: PMC7413840. | Wrong population |
| Oba A, Stoop TF, Löhr M, Hackert T, Zyromski N, Nealon WH, Unno M, Schulick RD, Al-Musawi MH, Wu W, Zhao Y, Satoi S, Wolfgang CL, Abu Hilal M, Besselink MG, Del Chiaro M; Pancreas Club, European Pancreatic Club, Chinese Pancreatic Surgery Association, European Consortium on Minimally Invasive Pancreatic Surgery, Study Group of Preoperative Therapy for Pancreatic Cancer, Study Group of Pancreatic Ductal Adenocarcinoma with Peritoneal Metastasis and International Study Group on Cystic Tumors of the Pancreas. Global Survey on Pancreatic Surgery During the COVID-19 Pandemic. Ann Surg. 2020 Aug;272(2):e87-e93. doi: 10.1097/SLA.0000000000004006. PMID: 32675507; PMCID: PMC7268883. | Wrong study design |
| Oertel M, Elsayad K, Engenhart-Cabillic R, Reinartz G, Baues C, Schmidberger H, Vordermark D, Marnitz S, Lukas P, Ruebe C, Engert A, Lenz G, Eich HT. Radiation treatment of hemato-oncological patients in times of the COVID-19 pandemic : Expert recommendations from the radiation oncology panels of the German Hodgkin Study Group and the German Lymphoma Alliance. Strahlenther Onkol. 2020 Dec;196(12):1096-1102. doi: 10.1007/s00066-020-01705-w. Epub 2020 Oct 30. PMID: 33125504; PMCID: PMC7596809. | Wrong study design |
| Orazem M, Oblak I, Spanic T, Ratosa I. Telemedicine in Radiation Oncology Post-COVID-19 Pandemic: There Is No Turning Back. Int J Radiat Oncol Biol Phys. 2020 Oct 1;108(2):411-415. doi: 10.1016/j.ijrobp.2020.06.052. PMID: 32890523; PMCID: PMC7462837. | Wrong outcome |
| Ota I, Asada Y. The impact of preoperative screening system on head and neck cancer surgery during the COVID-19 pandemic: Recommendations from the nationwide survey in Japan. Auris Nasus Larynx. 2020 Aug;47(4):687-691. doi: 10.1016/j.anl.2020.05.006. Epub 2020 May 16. PMID: 32425317; PMCID: PMC7229909. | Wrong study design |
| Palandri F, Piciocchi A, De Stefano V, Breccia M, Finazzi G, Iurlo A, Fazi P, Soddu S, Martino B, Siragusa S, Albano F, Passamonti F, Vignetti M, Vannucchi AM. How the coronavirus pandemic has affected the clinical management of Philadelphia-negative chronic myeloproliferative neoplasms in Italy-a GIMEMA MPN WP survey. Leukemia. 2020 Oct;34(10):2805-2808. doi: 10.1038/s41375-020-0953-3. Epub 2020 Jul 3. PMID: 32620840; PMCID: PMC7333222. | Wrong study design |
| Papautsky EL, Hamlish T. Patient-reported treatment delays in breast cancer care during the COVID-19 pandemic. Breast Cancer Res Treat. 2020 Nov;184(1):249-254. doi: 10.1007/s10549-020-05828-7. Epub 2020 Aug 9. PMID: 32772225; PMCID: PMC7415197. | Wrong population |
| Pilar A, Gravel SB, Croke J, Soliman H, Chung P, Wong RKS. Coronavirus Disease 2019's (COVID-19's) Silver Lining-Through the Eyes of Radiation Oncology Fellows. Adv Radiat Oncol. 2021 Jan-Feb;6(1):100527. doi: 10.1016/j.adro.2020.07.004. Epub 2020 Jul 23. PMID: 32838070; PMCID: PMC7377812. | Wrong outcome |
| Polak WG, Fondevila C, Karam V, Adam R, Baumann U, Germani G, Nadalin S, Taimr P, Toso C, Troisi RI, Zieniewicz K, Belli LS, Duvoux C. Impact of COVID-19 on liver transplantation in Europe: alert from an early survey of European Liver and Intestine Transplantation Association and European Liver Transplant Registry. Transpl Int. 2020 Oct;33(10):1244-1252. doi: 10.1111/tri.13680. Epub 2020 Aug 13. PMID: 32609908; PMCID: PMC7361228. | Wrong population |
| Prakash G, Shetty P, Thiagarajan S, Gulia A, Pandrowala S, Singh L, Thorat V, Patil V, Divatia JV, Puri A, Pramesh CS. Compliance and perception about personal protective equipment among health care workers involved in the surgery of COVID-19 negative cancer patients during the pandemic. J Surg Oncol. 2020 Nov;122(6):1013-1019. doi: 10.1002/jso.26151. Epub 2020 Aug 3. PMID: 32748476; PMCID: PMC7436474. | Wrong outcome |
| Price SJ, Joannides A, Plaha P, Afshari FT, Albanese E, Barua NU, Chan HW, Critchley G, Flannery T, Fountain DM, Mathew RK, Piper RJ, Poon MT, Rajaraman C, Rominiyi O, Smith S, Solomou G, Solth A, Surash S, Wykes V, Watts C, Bulbeck H, Hutchinson P, Jenkinson MD; COVID-CNSMDT study group. Impact of COVID-19 pandemic on surgical neuro-oncology multi-disciplinary team decision making: a national survey (COVID-CNSMDT Study). BMJ Open. 2020 Aug 16;10(8):e040898. doi: 10.1136/bmjopen-2020-040898. PMID: 32801210; PMCID: PMC7430412. | Wrong outcome |
| Reuter-Oppermann M, Müller-Polyzou R, Wirtz H, Georgiadis A. Influence of the pandemic dissemination of COVID-19 on radiotherapy practice: A flash survey in Germany, Austria and Switzerland. PLoS One. 2020 May 21;15(5):e0233330. doi: 10.1371/journal.pone.0233330. PMID: 32437381; PMCID: PMC7241763. | Wrong study design |
| Rocco N, Montagna G, Di Micco R, Benson J, Criscitiello C, Chen L, Di Pace B, Esgueva Colmenarejo AJ, Harder Y, Karakatsanis A, Maglia A, Mele M, Nafissi N, Ferreira PS, Taher W, Tejerina A, Vinci A, Nava M, Catanuto G. The Impact of the COVID-19 Pandemic on Surgical Management of Breast Cancer: Global Trends and Future Perspectives. Oncologist. 2021 Jan;26(1):e66-e77. doi: 10.1002/onco.13560. Epub 2020 Nov 25. PMID: 33044007; PMCID: PMC7675306. | Wrong study design |
| Rodler S, Apfelbeck M, Schulz GB, Ivanova T, Buchner A, Staehler M, Heinemann V, Stief C, Casuscelli J. Telehealth in Uro-oncology Beyond the Pandemic: Toll or Lifesaver? Eur Urol Focus. 2020 Sep 15;6(5):1097-1103. doi: 10.1016/j.euf.2020.05.010. Epub 2020 Jun 10. PMID: 32534969; PMCID: PMC7286644. | Wrong population |
| Rodríguez-Carrasco M, Albéniz E, Bhandari P, Beyna T, Bourke MJ, Min A, Chiu PWY, Chu S, Yip HC, Deprez PH, Emura F, Repici A, Suzuki N, Yahagi N, Kubosawa Y, Hassan C, Dinis-Ribeiro M. COVID-19 and endoscopic management of superficial gastrointestinal neoplastic lesions: a multinational cross-sectional survey. Endoscopy. 2021 Feb;53(2):173-177. doi: 10.1055/a-1237-5221. Epub 2020 Aug 11. PMID: 32781471; PMCID: PMC7869031. | Wrong outcome |
| Rosenberg AR, Weaver MS, Fry A, Wiener L. Exploring the Impact of the Coronavirus Pandemic on Pediatric Palliative Care Clinician Personal and Professional Well-Being: A Qualitative Analysis of U.S. Survey Data. J Pain Symptom Manage. 2021 Apr;61(4):805-811. doi: 10.1016/j.jpainsymman.2020.09.037. Epub 2020 Sep 30. PMID: 33010337; PMCID: PMC7525352. | Wrong outcome |
| Rosenzweig B, Bex A, Dotan ZA, Frydenberg M, Klotz L, Lotan Y, Schulman CC, Tsaur I, Ramon J. Trends in urologic oncology clinical practice and medical education under COVID-19 pandemic: An international survey of senior clinical and academic urologists. Urol Oncol. 2020 Dec;38(12):929.e1-929.e10. doi: 10.1016/j.urolonc.2020.09.015. Epub 2020 Oct 7. PMID: 33036903; PMCID: PMC7539173. | Wrong outcome |
| Rubio-San-Simón A, André N, Cefalo MG, Aerts I, Castañeda A, Benezech S, Makin G, van Eijkelenburg N, Nysom K, Marshall L, Gambart M, Hladun R, Rossig C, Bergamaschi L, Fagioli F, Carpenter B, Ducassou S, Owens C, Øra I, Ribelles AJ, De Wilde B, Guerra-García P, Strullu M, Rizzari C, Ek T, Hettmer S, Gerber NU, Rawlings C, Diezi M, Palmu S, Ruggiero A, Verdú J, de Rojas T, Vassal G, Geoerger B, Moreno L, Bautista F. Impact of COVID-19 in paediatric early-phase cancer clinical trials in Europe: A report from the Innovative Therapies for Children with Cancer (ITCC) consortium. Eur J Cancer. 2020 Dec;141:82-91. doi: 10.1016/j.ejca.2020.09.024. Epub 2020 Oct 9. PMID: 33129040; PMCID: PMC7546235. | Wrong outcome |
| Rubio-San-Simón A, Verdú-Amorós J, Hladun R, Juan-Ribelles A, Molero M, Guerra-García P, Pérez-Martínez A, Castañeda A, Cañete A, de Rojas T, Moreno L, Bautista F. Challenges in early phase clinical trials for childhood cancer during the COVID-19 pandemic: a report from the new agents group of the Spanish Society of Paediatric Haematology and Oncology (SEHOP). Clin Transl Oncol. 2021 Jan;23(1):183-189. doi: 10.1007/s12094-020-02399-3. Epub 2020 May 29. PMID: 32472454; PMCID: PMC7258607. | Wrong outcome |
| Rymarowicz J, Stefura T, Major P, Szeliga J, Wallner G, Nowakowski M, Pędziwiatr M. General surgeons' attitudes towards COVID-19: A national survey during the SARS-CoV-2 virus outbreak. Eur Surg. 2020 Jul 16:1-6. doi: 10.1007/s10353-020-00649-w. Epub ahead of print. PMID: 32837516; PMCID: PMC7365027. | Wrong outcome |
| Salama M, Ataman-Millhouse L, Braham M, Berjeb K, Khrouf M, Rodrigues JK, Reis FM, Silva TC, Sánchez F, Romero S, Smitz J, Vásquez L, Vega M, Sobral F, Terrado G, Lombardi MG, Scarella A, Bourlon MT, Verduzco-Aguirre H, Sánchez AM, Adiga SK, Tholeti P, Udupa KS, Mahajan N, Patil M, Dalvi R, Venter C, Demetriou G, Geel J, Quintana R, Rodriguez G, Quintana T, Viale L, Fraguglia M, Coirini M, Remolina-Bonilla YA, Noguera JAR, Velásquez JC, Suarez A, Arango GD, Pineda JID, Aldecoa MDC, Javed M, Al Sufyan H, Daniels N, Oranye BC, Ogunmokun AA, Onwuzurigbo KI, Okereke CJ, Whesu TC, Woodruff TK. Installing oncofertility programs for common cancers in limited resource settings (Repro-Can-OPEN Study): An extrapolation during the global crisis of Coronavirus (COVID-19) pandemic. J Assist Reprod Genet. 2020 Jul;37(7):1567-1577. doi: 10.1007/s10815-020-01821-7. Epub 2020 Jun 27. PMID: 32594284; PMCID: PMC7320246. | Wrong outcome |
| Sarkhel S, Bakhla AK, Praharaj SK, Ghosal MK. Information overload regarding COVID-19: Adaptation and validation of the cancer information overload scale. Indian J Psychiatry. 2020 Sep-Oct;62(5):481-487. doi: 10.4103/psychiatry.IndianJPsychiatry_974_20. Epub 2020 Oct 10. PMID: 33678827; PMCID: PMC7909014. | Wrong study design |
| Sayari AJ, Harada GK, Louie PK, McCarthy MH, Nolte MT, Mallow GM, Siyaji Z, Germscheid N, Cheung JPY, Neva MH, El-Sharkawi M, Valacco M, Sciubba DM, Chutkan NB, An HS, Samartzis D. Personal Health of Spine Surgeons Can Impact Perceptions, Decision-Making and Healthcare Delivery During the COVID-19 Pandemic - A Worldwide Study. Neurospine. 2020 Jun;17(2):313-330. doi: 10.14245/ns.2040336.168. Epub 2020 Jun 30. PMID: 32615695; PMCID: PMC7338966. | Wrong outcome |
| Shanbhag NM, Duncan A, Santos EGD, Yazigi H, Grant-Tate M, Girgis NFF, MoyaSantos NB, Vinh-Hung V, Duran YL. Results of the Survey Conducted Among Caribbean Physicians on a Zoom Meeting Discussing the Article "A Practical Approach to the Management of Cancer Patients During the Novel Coronavirus Disease 2019 (COVID-19) Pandemic: An International Collaborative Group". Oncologist. 2020 Dec;25(12):e2024-e2028. doi: 10.1002/onco.13556. Epub 2020 Oct 29. PMID: 33030774; PMCID: PMC7675247. | Wrong study design |
| Shinan-Altman S, Levkovich I, Tavori G. Healthcare utilization among breast cancer patients during the COVID-19 outbreak. Palliat Support Care. 2020 Aug;18(4):385-391. doi: 10.1017/S1478951520000516. PMID: 32594966; PMCID: PMC7360944. | Wrong population |
| Sigorski D, Sobczuk P, Osmola M, Kuć K, Walerzak A, Wilk M, Ciszewski T, Kopeć S, Hryń K, Rutkowski P, Stec R, Szczylik C, Bodnar L. Impact of COVID-19 on anxiety levels among patients with cancer actively treated with systemic therapy. ESMO Open. 2020 Oct;5(5):e000970. doi: 10.1136/esmoopen-2020-000970. PMID: 33097653; PMCID: PMC7590347. | Wrong population |
| Singh N, Kumar S, Rathore P, Vig S, Vallath N, Mohan A, Bhatnagar S. Concerns and Coping Strategies of Persons Under Institutional Quarantine During SARS-CoV-2 Pandemic. Indian J Palliat Care. 2020 Jun;26(Suppl 1):S99-S105. doi: 10.4103/IJPC.IJPC_176_20. Epub 2020 Jun 30. PMID: 33088098; PMCID: PMC7534984. | Wrong population |
| Singhi EK, Dupuis MM, Ross JA, Rieber AG, Bhadkamkar NA. Medical Hematology/Oncology Fellows' Perceptions of Online Medical Education During the COVID-19 Pandemic. J Cancer Educ. 2020 Oct;35(5):1034-1040. doi: 10.1007/s13187-020-01863-6. PMID: 32888144; PMCID: PMC7473824. | Wrong outcome |
| Smrke A, Younger E, Wilson R, Husson O, Farag S, Merry E, Macklin-Doherty A, Cojocaru E, Arthur A, Benson C, Miah AB, Zaidi S, Gennatas S, Jones RL. Telemedicine During the COVID-19 Pandemic: Impact on Care for Rare Cancers. JCO Glob Oncol. 2020 Jul;6:1046-1051. doi: 10.1200/GO.20.00220. PMID: 32639877; PMCID: PMC7392777. | Wrong outcome |
| Spencer-Bowdage S, Russell B, Rigby J, O'Kelly J, Kelly P, Page M, Raw C, Allchorne P, Harper P, Crew J, Kockelbergh R, Knight A, Van Hemelrijck M, Bryan RT. The experience of UK patients with bladder cancer during the COVID-19 pandemic: a survey-based snapshot. BJU Int. 2021 Feb;127(2):179-181. doi: 10.1111/bju.15287. Epub 2020 Nov 16. PMID: 33124729; PMCID: PMC7894556. | Wrong population |
| Sprik P, Keenan AJ, Boselli D, Cheeseboro S, Meadors P, Grossoehme D. Feasibility and acceptability of a telephone-based chaplaincy intervention in a large, outpatient oncology center. Support Care Cancer. 2021 Mar;29(3):1275-1285. doi: 10.1007/s00520-020-05598-4. Epub 2020 Jul 4. PMID: 32623520; PMCID: PMC7334628. | Wrong population |
| Staehler M, Battle D, Pal SK, Bergerot CD. Counterbalancing COVID-19 with Cancer Surveillance and Therapy: A Survey of Patients with Renal Cell Carcinoma. Eur Urol Focus. 2021 Nov;7(6):1355-1362. doi: 10.1016/j.euf.2020.09.002. Epub 2020 Sep 11. PMID: 32943372; PMCID: PMC7486070. | Wrong population |
| Staehler MD, Battle DJ, Bergerot CD, Pal SK, Penson DF. COVID-19 and financial toxicity in patients with renal cell carcinoma. World J Urol. 2021 Jul;39(7):2559-2565. doi: 10.1007/s00345-020-03476-6. Epub 2020 Oct 22. PMID: 33090258; PMCID: PMC7578440. | Wrong population |
| Swainston J, Chapman B, Grunfeld EA, Derakshan N. COVID-19 Lockdown and Its Adverse Impact on Psychological Health in Breast Cancer. Front Psychol. 2020 Aug 24;11:2033. doi: 10.3389/fpsyg.2020.02033. PMID: 32982846; PMCID: PMC7476556. | Wrong population |
| Sylvia V. Alarcon, Omid Salehi, Eduardo A. Vega, Vera Kazakova and Claudius Conrad, Abstract PO-027: Multi-institutional survey of COVID-19 impact in cancer care in patients and health care providers, Clin Cancer Res September 15 2020 (26) (18 Supplement) PO-027; DOI: 10.1158/1557-3265.COVID-19-PO-027 | Wrong outcome |
| Tagliamento M, Spagnolo F, Poggio F, Soldato D, Conte B, Ruelle T, Barisione E, De Maria A, Del Mastro L, Di Maio M, Lambertini M. Italian survey on managing immune checkpoint inhibitors in oncology during COVID-19 outbreak. Eur J Clin Invest. 2020 Sep;50(9):e13315. doi: 10.1111/eci.13315. Epub 2020 Jul 5. PMID: 32535890; PMCID: PMC7323025. | Wrong study design |
| Tashkandi E, BaAbdullah M, Zeeneldin A, AlAbdulwahab A, Elemam O, Elsamany S, Alfayez M, Dabash Y, Khayat E, Hassanin F, Abdulhameed R, Jazieh AR. Optimizing the Communication with Cancer Patients During the COVID-19 Pandemic: Patient Perspectives. Patient Prefer Adherence. 2020 Jul 20;14:1205-1212. doi: 10.2147/PPA.S263022. PMID: 32764893; PMCID: PMC7381793. | Wrong population |
| Thomaier L, Teoh D, Jewett P, Beckwith H, Parsons H, Yuan J, Blaes AH, Lou E, Hui JYC, Vogel RI. Emotional health concerns of oncology physicians in the United States: Fallout during the COVID-19 pandemic. PLoS One. 2020 Nov 24;15(11):e0242767. doi: 10.1371/journal.pone.0242767. PMID: 33232377; PMCID: PMC7685431. | Wrong outcome |
| Thomson DJ, Palma D, Guckenberger M, Balermpas P, Beitler JJ, Blanchard P, Brizel D, Budach W, Caudell J, Corry J, Corvo R, Evans M, Garden AS, Giralt J, Gregoire V, Harari PM, Harrington K, Hitchcock YJ, Johansen J, Kaanders J, Koyfman S, Langendijk JA, Le QT, Lee N, Margalit D, Mierzwa M, Porceddu S, Soong YL, Sun Y, Thariat J, Waldron J, Yom SS. Practice Recommendations for Risk-Adapted Head and Neck Cancer Radiation Therapy During the COVID-19 Pandemic: An ASTRO-ESTRO Consensus Statement. Int J Radiat Oncol Biol Phys. 2020 Jul 15;107(4):618-627. doi: 10.1016/j.ijrobp.2020.04.016. Epub 2020 Apr 14. PMID: 32302681; PMCID: PMC7194855. | Wrong study design |
| Tran VT, Ravaud P. COVID-19-related perceptions, context and attitudes of adults with chronic conditions: Results from a cross-sectional survey nested in the ComPaRe e-cohort. PLoS One. 2020 Aug 6;15(8):e0237296. doi: 10.1371/journal.pone.0237296. PMID: 32760127; PMCID: PMC7410193. | Wrong population |
| Triantafillou V, Layfield E, Prasad A, Deng J, Shanti RM, Newman JG, Rajasekaran K. Patient Perceptions of Head and Neck Ambulatory Telemedicine Visits: A Qualitative Study. Otolaryngol Head Neck Surg. 2021 May;164(5):923-931. doi: 10.1177/0194599820943523. Epub 2020 Jul 14. PMID: 32662749. | Wrong population |
| Turner S, Pham T, Robledo K, Turner S, Brown C, Sundaresan P. Rapid Adaptation of Cancer Education in Response to the COVID-19 Pandemic: Evaluation of a Live Virtual Statistics and Research Skills Workshop for Oncology Trainees. J Cancer Educ. 2020 Oct 21:1–6. doi: 10.1007/s13187-020-01898-9. Epub ahead of print. PMID: 33089455; PMCID: PMC7577357. | Wrong outcome |
| Ürün Y, Hussain SA, Bakouny Z, Castellano D, Kılıçkap S, Morgan G, Mckay RR, Pels K, Schmidt A, Doroshow DB, Schütz F, Albiges L, Lopes G, Catto JWF, Peters S, Choueiri TK. Survey of the Impact of COVID-19 on Oncologists' Decision Making in Cancer. JCO Glob Oncol. 2020 Aug;6:1248-1257. doi: 10.1200/GO.20.00300. PMID: 32755479; PMCID: PMC7456315. | Wrong study design |
| Van De Poll-franse LV, de Rooij B, Horevoorts N, May AM, Vink G, Koopman M, van Laarhoven HWM, Besselink M, Oerlemans S, Husson O, Beijer S, Ezendam N, Raijmakers N, Wollersheim B, Hoedjes M, Siesling S, van Eenbergen M, Mols F. 1686P The impact of the COVID-19 crisis on perceived changes in care and wellbeing of cancer patients and norm participants: Results of the PROFILES registry. Ann Oncol. 2020 Sep;31:S997–8. doi: 10.1016/j.annonc.2020.08.1750. Epub 2020 Sep 22. PMCID: PMC7506350. | Wrong population |
| van de Poll-Franse LV, de Rooij BH, Horevoorts NJE, et al. Perceived Care and Well-being of Patients With Cancer and Matched Norm Participants in the COVID-19 Crisis: Results of a Survey of Participants in the Dutch PROFILES Registry. *JAMA Oncol.* 2021;7(2):279–284. doi:10.1001/jamaoncol.2020.6093 | Wrong population |
| Vanni G, Materazzo M, Santori F, Pellicciaro M, Costesta M, Orsaria P, Cattadori F, Pistolese CA, Perretta T, Chiocchi M, Meucci R, Lamacchia F, Assogna M, Caspi J, Granai AV, DE Majo A, Chiaravalloti A, D'Angelillo MR, Barbarino R, Ingallinella S, Morando L, Dalli S, Portarena I, Altomare V, Tazzioli G, Buonomo OC. The Effect of Coronavirus (COVID-19) on Breast Cancer Teamwork: A Multicentric Survey. In Vivo. 2020 Jun;34(3 Suppl):1685-1694. doi: 10.21873/invivo.11962. PMID: 32503830; PMCID: PMC8378028. | Wrong study design |
| Varani S, Ostan R, Franchini L, Ercolani G, Pannuti R, Biasco G, Bruera E. Caring Advanced Cancer Patients at Home During COVID-19 Outbreak: Burnout and Psychological Morbidity Among Palliative Care Professionals in Italy. J Pain Symptom Manage. 2021 Feb;61(2):e4-e12. doi: 10.1016/j.jpainsymman.2020.11.026. Epub 2020 Nov 27. PMID: 33249082; PMCID: PMC7691143. | Wrong outcome |
| Vigliar E, Cepurnaite R, Alcaraz-Mateos E, Ali SZ, Baloch ZW, Bellevicine C, Bongiovanni M, Botsun P, Bruzzese D, Bubendorf L, Büttner R, Canberk S, Capitanio A, Casadio C, Cazacu E, Cochand-Priollet B, D'Amuri A, Eloy C, Engels M, Fadda G, Fontanini G, Fulciniti F, Hofman P, Iaccarino A, Ieni A, Jiang XS, Kakudo K, Kern I, Kholova I, Liu C, Lobo A, Lozano MD, Malapelle U, Maleki Z, Michelow P, Musayev J, Özgün G, Oznur M, Peiró Marqués FM, Pisapia P, Poller D, Pyzlak M, Robinson B, Rossi ED, Roy-Chowdhuri S, Saieg M, Savic Prince S, Schmitt FC, Javier Seguí Iváñez F, Štoos-Veić T, Sulaieva O, Sweeney BJ, Tuccari G, van Velthuysen ML, VanderLaan PA, Vielh P, Viola P, Voorham R, Weynand B, Zeppa P, Faquin WC, Pitman MB, Troncone G. Global impact of the COVID-19 pandemic on cytopathology practice: Results from an international survey of laboratories in 23 countries. Cancer Cytopathol. 2020 Dec;128(12):885-894. doi: 10.1002/cncy.22373. Epub 2020 Oct 27. PMID: 33108683. | Wrong population |
| Voisin MR, Oliver K, Farrimond S, Chee T, Arzbaecher J, Kruchko C, Maher ME, Tse C, Cashman R, Daniels M, Mungoshi C, Lamb S, Granero A, Lovely M, Baker J, Payne S, Zadeh G. Brain tumors and COVID-19: the patient and caregiver experience. Neurooncol Adv. 2020 Aug 23;2(1):vdaa104. doi: 10.1093/noajnl/vdaa104. PMID: 32989433; PMCID: PMC7499687. | Wrong population |
| Wang Y, Duan Z, Ma Z, Mao Y, Li X, Wilson A, Qin H, Ou J, Peng K, Zhou F, Li C, Liu Z, Chen R. Epidemiology of mental health problems among patients with cancer during COVID-19 pandemic. Transl Psychiatry. 2020 Jul 31;10(1):263. doi: 10.1038/s41398-020-00950-y. PMID: 32737292; PMCID: PMC7393344. | Wrong population |
| Waterhouse DM, Harvey RD, Hurley P, Levit LA, Kim ES, Klepin HD, Mileham KF, Nowakowski G, Schenkel C, Davis C, Bruinooge SS, Schilsky RL. Early Impact of COVID-19 on the Conduct of Oncology Clinical Trials and Long-Term Opportunities for Transformation: Findings From an American Society of Clinical Oncology Survey. JCO Oncol Pract. 2020 Jul;16(7):417-421. doi: 10.1200/OP.20.00275. Epub 2020 May 12. PMID: 32396491. | Wrong outcome |
| Wu B, Zhao Y, Xu D, Wang Y, Niu N, Zhang M, Zhi X, Zhu P, Meng A. Factors associated with nurses' willingness to participate in care of patients with COVID-19: A survey in China. J Nurs Manag. 2020 Oct;28(7):1704-1712. doi: 10.1111/jonm.13126. Epub 2020 Sep 12. PMID: 32767852; PMCID: PMC7436655. | Wrong population |
| Wu QL, Street RL Jr. Factors affecting cancer patients' electronic communication with providers: Implications for COVID-19 induced transitions to telehealth. Patient Educ Couns. 2020 Sep 28;103(12):2583–7. doi: 10.1016/j.pec.2020.09.036. Epub ahead of print. PMID: 33036815; PMCID: PMC7521379. | Wrong population |
| Yang S, Dong D, Gu H, Gale RP, Ma J, Huang X. Impact of stopping therapy during the SARS-CoV-2 pandemic in persons with lymphoma. J Cancer Res Clin Oncol. 2021 May;147(5):1469-1479. doi: 10.1007/s00432-020-03426-0. Epub 2020 Oct 19. PMID: 33078214; PMCID: PMC7571863. | Wrong population |
| Younger E, Smrke A, Lidington E, Farag S, Ingley K, Chopra N, Maleddu A, Augustin Y, Merry E, Wilson R, Benson C, Miah A, Zaidi S, McTiernan A, Strauss SJ, Dileo P, Gennatas S, Husson O, Jones RL. Health-Related Quality of Life and Experiences of Sarcoma Patients during the COVID-19 Pandemic. Cancers (Basel). 2020 Aug 14;12(8):2288. doi: 10.3390/cancers12082288. PMID: 32823999; PMCID: PMC7547383. | Wrong population |
| Zaid W, Schlieve T. The Early Effects of Coronavirus Disease-2019 on Head and Neck Oncology and Microvascular Reconstruction Practice: A National Survey of Oral and Maxillofacial Surgeons Enrolled in the Head and Neck Special Interest Group. J Oral Maxillofac Surg. 2020 Oct;78(10):1859-1868. doi: 10.1016/j.joms.2020.07.012. Epub 2020 Jul 17. PMID: 32745533; PMCID: PMC7366104. | Wrong study design |
| Zhang H, Cha EE, Lynch K, Cahlon O, Gomez DR, Shaverdian N, Gillespie EF. Radiation Oncologist Perceptions of Telemedicine from Consultation to Treatment Planning: A Mixed-Methods Study. Int J Radiat Oncol Biol Phys. 2020 Oct 1;108(2):421-429. doi: 10.1016/j.ijrobp.2020.07.007. PMID: 32890525; PMCID: PMC7462757. | Wrong study design |
| Zimmerman BS, Seidman D, Berger N, Cascetta KP, Nezolosky M, Trlica K, Ryncarz A, Keeton C, Moshier E, Tiersten A. Patient Perception of Telehealth Services for Breast and Gynecologic Oncology Care during the COVID-19 Pandemic: A Single Center Survey-based Study. J Breast Cancer. 2020 Oct 19;23(5):542-552. doi: 10.4048/jbc.2020.23.e56. PMID: 33154829; PMCID: PMC7604367. | Wrong population |
| Zoia C, Raffa G, Somma T, Della Pepa GM, La Rocca G, Zoli M, Bongetta D, De Divitiis O, Fontanella MM. COVID-19 and neurosurgical training and education: an Italian perspective. Acta Neurochir (Wien). 2020 Aug;162(8):1789-1794. doi: 10.1007/s00701-020-04460-0. Epub 2020 Jun 18. PMID: 32556815; PMCID: PMC7302726. | Wrong outcome |
| Zuliani S, Zampiva I, Tregnago D, Casali M, Cavaliere A, Fumagalli A, Merler S, Riva ST, Rossi A, Zacchi F, Zaninotto E, Auriemma A, Pavarana M, Soldà C, Benini L, Borghesani M, Caldart A, Casalino S, Gaule M, Kadrija D, Mongillo M, Pesoni C, Biondani P, Cingarlini S, Fiorio E, Melisi D, Parolin V, Tondulli L, Belluomini L, Zecchetto C, Avesani B, Biasi A, Bovo C, Dazzani E, Dodi A, Gelmini S, Leta LC, Lo Cascio G, Lombardo F, Lucin E, Martinelli IA, Messineo L, Moscarda V, Pafumi S, Reni A, Sartori G, Scaglione IM, Shoval Y, Sposito M, Tacconelli E, Trestini I, Zambonin V, Zanelli S, Pilotto S, Milella M. Organisational challenges, volumes of oncological activity and patients' perception during the severe acute respiratory syndrome coronavirus 2 epidemic. Eur J Cancer. 2020 Aug;135:159-169. doi: 10.1016/j.ejca.2020.05.029. Epub 2020 Jun 11. PMID: 32580131; PMCID: PMC7287451. | Wrong study design |

1. **RISK OF BIAS OF INCLUDED STUDIES**

|  | 1. Is the source population representative of the population of interest? | 2. Is the response rate adequate? | 3. Is there little missing data? | 4. Is the survey clinically sensible? |
| --- | --- | --- | --- | --- |
| A national survey on radiation oncology patterns of practice in Switzerland during the COVID-19 pandemic- Present changes and future perspectives | Definitely yes | Probably yes | Definitely yes | Probably yes |
| A Snapshot of Elective Oncological Surgery in Italy During COVID-19 Emergency- Pearls, Pitfalls, and Perspectives | Definitely yes | Definitely yes | Definitely yes | Definitely yes |
| A snapshot of European neurosurgery December 2019 vs. March 2020- just before and during the Covid-19 pandemic | Probably yes | Probably yes | Definitely yes | Probably yes |
| A snapshot on radiotherapy for head and neck cancer patients during the COVID-19 pandemic- a survey of the Italian Association of Radiotherapy and Clinical Oncology (AIRO) head and neck working group | Definitely yes | Probably no | Probably no | Probably yes |
| Adjusting to the new reality- Evaluation of early practice pattern adaptations to the COVID-19 pandemic | Definitely yes | Probably no | Probably yes | Definitely yes |
| Alternative management for gynecological cancer care during the COVID-2019 pandemic- A Latin American survey | Definitely yes | Probably no | Probably no | Probably yes |
| Assessing the impact of COVID-19 on the management of patients with liver diseases- A national survey by the Italian association for the study of the Liver | Definitely yes | Definitely no | Probably no | Probably yes |
| Assessing the impact of the COVID-19 outbreak on the attitudes and practice of Italian oncologists toward breast cancer care and related research activities | Definitely yes | Definitely no | Definitely yes | Probably yes |
| Back to (new) normality ”A CODRAL/AIRO-L survey on cancer radiotherapy in Lombardy during Italian COVID-19 phase 2 | Definitely yes | Definitely yes | Definitely yes | Definitely yes |
| Cancer care in a Western Indian tertiary center during the pandemic- Surgeon's perspective | Definitely yes | Definitely yes | Definitely yes | Definitely yes |
| Cancer Management during the COVID-19 Pandemic in the United States- Results from a National Physician Cross-sectional Survey | Definitely yes | Probably yes | Probably yes | Definitely yes |
| Change in practice in gynecologic oncology during the COVID-19 pandemic- A social media survey | Probably yes | Definitely no | Definitely yes | Definitely yes |
| Changes in breast cancer management during the Corona Virus Disease 19 pandemic- An international survey of the European Breast Cancer Research Association of Surgical Trialists (EUBREAST) | Probably yes | Probably no | Probably yes | Definitely yes |
| Chronic myeloid leukemia management at the time of the COVID-19 pandemic in Italy. A campus CML survey. | Probably yes | Definitely yes | Probably yes | Probably no |
| Colorectal surgery in Italy during the Covid19 outbreak- a survey from the iCral study group. | Probably yes | Definitely yes | Definitely yes | Definitely yes |
| COVID-19 and its impact on gynaecologic oncology practice in India-results of a nationwide survey | Probably yes | Definitely no | Probably yes | Definitely yes |
| COVID-19 and skin cancer management- French nation-wide questionnaire survey from real-life practice | Definitely yes | Definitely no | Definitely yes | Probably yes |
| COVID-19 and the Global Impact on Colorectal Practice and Surgery | Probably no | Definitely no | Probably yes | Probably yes |
| COVID-19 outbreak and cancer radiotherapy disruption in Italy- Survey endorsed by the Italian Association of Radiotherapy and Clinical Oncology (AIRO) | Definitely yes | Probably yes | Definitely yes | Definitely yes |
| COVID's Impact on Radiation Oncology- A Latin American Survey Study | Probably yes | Probably no | Definitely yes | Definitely yes |
| Delivery of hepato-pancreato-biliary surgery during the COVID-19 pandemic- an European-African Hepato-Pancreato-Biliary Association (E-AHPBA) cross-sectional survey | Definitely yes | Definitely no | Definitely yes | Definitely yes |
| Early impact of the COVID-19 pandemic on paediatric cancer care in Latin America | Definitely yes | Probably yes | Definitely yes | Definitely yes |
| Early practices in endonasal skull base surgery during the COVID-19 pandemic/ a global survey | Probably yes | Definitely no | Probably yes | Definitely yes |
| Effect of coronavirus disease 2019 on urological surgery services and training up to the peak of the pandemic in South East England | Probably yes | Definitely no | Probably yes | Probably yes |
| Evaluating the impact of COVID-19 on medical oncology workforce and cancer care in Canada | Definitely yes | Definitely no | Probably yes | Probably yes |
| Exploring Urological Experience in the COVID-19 Outbreak- American Confederation of Urology (CAU) Survey | Definitely yes | Probably no | Definitely yes | Definitely yes |
| Fighting cancer in coronavirus disease era- organization of work in medical oncology departments in Emilia Romagna region of Italy. | Definitely yes | Definitely yes | Definitely yes | Definitely yes |
| Global change of surgical and oncological clinical practice in urology during early COVID-19 pandemic | Probably no | Definitely no | Probably yes | Probably yes |
| Global Impact of COVID-19 on Nuclear Medicine Departments- An International Survey in April 2020 | Probably yes | Probably no | Probably yes | Definitely yes |
| Gynaecologic cancer care duringCOVID-19 pandemic in India- a social media survey | Probably yes | Definitely no | Probably yes | Definitely no |
| Impact of covid-19 in gynecologic oncology- A nationwide italian survey of the sigo and mito groups | Definitely yes | Probably yes | Probably yes | Definitely yes |
| Impact of COVID-19 on canadian medical oncologists and cancer care- Canadian association of medical oncologists survey report | Definitely yes | Definitely no | Probably yes | Probably yes |
| Impact of COVID-19 on cancer care- A survey from the French Society of Pediatric Oncology (SFCE) | Definitely yes | Definitely yes | Probably yes | Probably yes |
| Impact of COVID-19 on cancer service delivery- Results from an international survey of oncology clinicians | Probably yes | Probably no | Probably yes | Definitely yes |
| Impact of COVID-19 outbreak on cancer immunotherapy in Italy- A survey of young oncologists | Probably yes | Probably no | Definitely yes | Probably yes |
| Impact of COVID-19 outbreak on esophageal cancer surgery in Northern Italy- lessons learned from a multicentric snapshot. | Definitely yes | Definitely yes | Probably yes | Definitely yes |
| Impact of COVID-19 Pandemic on Gynecological Oncology Care- Glimpse into Association of Gynecological Oncologists of India (AGOI) Perspective | Definitely yes | Definitely no | Probably no | Probably yes |
| Impact of COVID-19 pandemic on Italian Otolaryngology Units/ a nationwide study. | Definitely yes | Probably no | Probably yes | Definitely yes |
| Impact of the coronavirus disease 2019 (COVID-19) pandemic on pediatric oncology care in the Middle East, North Africa, and West Asia region- A report from the Pediatric Oncology East and Mediterranean (POEM) group | Definitely yes | Definitely no | Probably yes | Probably yes |
| Impact of the COVID-19 pandemic in nuclear medicine departments- preliminary report of the first international survey. | Probably yes | Definitely yes | Probably yes | Probably yes |
| Impact of the COVID-19 Pandemic on Cancer Care- A Global Collaborative Study | Definitely yes | Probably no | Definitely yes | Definitely yes |
| Impact of the COVID-19 pandemic on cytology practice- An international survey in the Asia-Pacific region | Probably yes | Probably no | Probably yes | Probably yes |
| Impact of the COVID-19 pandemic on patients suffering from musculoskeletal tumours | Definitely yes | Definitely no | Probably yes | Probably yes |
| Impact of the SARS-CoV2 pandemic dissemination on the management of neuroendocrine neoplasia in Italy- a report from the Italian Association for Neuroendocrine Tumors (Itanet) | Definitely yes | Probably no | Probably yes | Probably yes |
| Management of CLL patients early in the COVID-19 pandemic- An international survey of CLL experts. | Probably yes | Definitely yes | Probably yes | Probably yes |
| Nationwide survey of COVID-19 prevention measures in Japanese radiotherapy departments via online questionnaire for radiation oncologists | Definitely yes | Definitely no | Definitely yes | Probably yes |
| Oncological care organisation during COVID-19 outbreak | Probably yes | Probably yes | Definitely yes | Probably yes |
| Oncological colorectal surgery during the COVID-19pandemic a national survey | Definitely yes | Probably no | Probably yes | Definitely yes |
| Perspectives on the COVID-19 pandemic impact on cardio-oncology- results from the COVID-19 International Collaborative Network survey. | Definitely yes | Probably no | Definitely yes | Definitely yes |
| Preparedness of the cancer hospitals and changes in oncosurgical practices during COVID-19 pandemic in India- A cross-sectional study | Probably yes | Probably no | Probably yes | Definitely yes |
| Provision of obstetrics and gynaecology services during the COVID-19 pandemic- a survey of junior doctors in the UK National Health Service | Probably yes | Definitely yes | Definitely yes | Probably yes |
| Reorganisation of medical oncology departments during the novel coronavirus disease-19 pandemic- a nationwide Italian survey | Definitely yes | Definitely yes | Probably yes | Probably yes |
| Safety measures in selected radiotherapy centres within Africa in the face of Covid-19 | Probably no | Probably no | Definitely yes | Probably yes |
| Sarcoma Care Practice in India During COVID Pandemic- A Nationwide Survey | Probably no | Probably no | Probably no | Probably yes |
| The impact of coronavirus disease 2019 on the practice of thoracic oncology surgery- a survey of members of the European Society of Thoracic Surgeons (ESTS). | Definitely yes | Definitely no | Definitely yes | Probably yes |
| The influence of the SARS-CoV-2 pandemic on esophagogastric cancer services- An international survey of esophagogastric surgeons | Definitely yes | Probably no | Probably yes | Definitely yes |
| The Shifting Landscape of Genitourinary Oncology During the COVID-19 Pandemic and how Italian Oncologists Reacted- Results from a National Survey | Definitely yes | Definitely no | Definitely yes | Probably yes |
| To defer or not to defer? A German longitudinal multicentric assessment of clinical practice in urology during the COVID-19 pandemic | Definitely yes | Definitely no | Probably yes | Definitely yes |
| Virtual management of patients with cancer during the COVID-19 pandemic- Web-based questionnaire study | Definitely yes | Definitely no | Definitely yes | Probably yes |
| What is the preparedness and capacity of palliative care services in Middle-Eastern and North African countries to respond to COVID-19? A rapid survey. | Probably yes | Definitely no | Definitely yes | Probably yes |

Modified from “Risk of Bias Instrument for Cross-sectional Surveys of Attitudes and Practices”, contributed by the CLARITY Group at McMaster University.

Item 5 (“Is there any evidence for the reliability and validity of the survey instrument?”) has been removed because no one of the surveys underwent rigorous and formal clinical validation.

1. **METAREGRESSION MODELS**

| Outcome | Model | Meta-regression estimate (SE) | P-value | Accounted heterogeneity | Residual heterogeneity | P-value for test of residual heterogeneity | P-value for test of moderators | Number of studies |
| --- | --- | --- | --- | --- | --- | --- | --- | --- |
| **Treatment delay/cancellation** | - | - | - | 15.93% | 95.69% | <0.0001 | 0.43 | 17 |
|  | Intercept | 0.94 (0.35) | 0.008 |  |  |  |  |  |
|  | Asia | 0.2 (0.19) | 0.3 |  |  |  |  |  |
|  | Europe | 0.33 (0.18) | 0.07 |  |  |  |  |  |
|  | Japan | 0.007 (0.26) | 0.98 |  |  |  |  |  |
|  | Middle East | 0.07 (0.2) | 0.73 |  |  |  |  |  |
|  | North America | 0.3 (0.27) | 0.27 |  |  |  |  |  |
|  | South America | Reference |  |  |  |  |  |  |
|  | Week of survey beginning | "-0.02 (0.02) | 0.4 |  |  |  |  |  |
| **Visits delay** | - | - | - | 87.32% | 58.03% | 0.049 | <0.0001 | 9 |
|  | Intercept | 0.54 (0.12) | <0.0001 |  |  |  |  |  |
|  | Asia | "-0.21 (0.22) | 0.34 |  |  |  |  |  |
|  | Europe | 0.7 (0.14) | <0.0001 |  |  |  |  |  |
|  | Japan | 0.37 (0.18) | 0.04 |  |  |  |  |  |
|  | North America | 0.54 (0.18) | 0.002 |  |  |  |  |  |
|  | South America | Reference |  |  |  |  |  |  |
|  | - | - | - | 100% | 0% | 0.4 | <0.0001 | 9 |
|  | Intercept | "-7.8 (3.3) | 0.02 |  |  |  |  |  |
|  | Asia | 7.9 (3.2) | 0.01 |  |  |  |  |  |
|  | Europe | 8.6 (3.1) | 0.005 |  |  |  |  |  |
|  | Japan | 5.3 (1.9) | 0.006 |  |  |  |  |  |
|  | North America | 5.98 (2.12) | 0.005 |  |  |  |  |  |
|  | South America | Reference |  |  |  |  |  |  |
|  | Sample size | 0.002 (0.007) | 0.01 |  |  |  |  |  |
| **Treatment modification** | - | - | - | 31.03% | 96.88% | <0.0001 | 0.02 | 9 |
|  | Intercept | 0.95 (0.21) | <0.0001 |  |  |  |  |  |
|  | Asia | 0.27 (0.25) | 0.28 |  |  |  |  |  |
|  | Europe | "-0.49 (0.3) | 0.1 |  |  |  |  |  |
|  | North America | "-0.07 (0.24) | 0.76 |  |  |  |  |  |
|  | South America | Reference |  |  |  |  |  |  |
|  | - | - | - | 85.2% | 86.99% | <0.0001 | <0.0001 | 9 |
|  | Intercept | 2.41 (0.37) | <0.0001 |  |  |  |  |  |
|  | Asia | 0.82 (0.18) | <0.0001 |  |  |  |  |  |
|  | Europe | "-0.4 (0.14) | 0.005 |  |  |  |  |  |
|  | North America | "-0.07 (0.11) | 0.52 |  |  |  |  |  |
|  | South America | Reference |  |  |  |  |  |  |
|  | Week of survey end | "-0.09 (0.02) | <0.0001 |  |  |  |  |  |
|  | - | - | - | 100% | 0% | 0.59 | <0.0001 | 9 |
|  | Intercept | 2.81 (0.16) | <0.0001 |  |  |  |  |  |
|  | Asia | 0.61 (0.08) | <0.0001 |  |  |  |  |  |
|  | Europe | "-0.87 (0.1) | <0.0001 |  |  |  |  |  |
|  | North America | "-0.09 (0.03) | 0.002 |  |  |  |  |  |
|  | South America | Reference |  |  |  |  |  |  |
|  | Week of survey end | "-0.09 (0.008) | <0.0001 |  |  |  |  |  |
|  | Oncology | "-0.47 (0.09) | <0.0001 |  |  |  |  |  |
|  | Radiotherapy | "-0.38 (0.09) | <0.0001 |  |  |  |  |  |
|  | Surgery | Reference |  |  |  |  |  |  |
| **Reduction of activity** | - | - | - | 40.46% | 88.25% | <0.0001 | 0.2 | 12 |
|  | Intercept | 1.3 (0.23) | <0.0001 |  |  |  |  |  |
|  | Europe | "-0.35 (0.21) | 0.1 |  |  |  |  |  |
|  | South America | Reference |  |  |  |  |  |  |
|  | Oncology | "-0.22 (0.17) | 0.18 |  |  |  |  |  |
|  | Radiotherapy | "-0.19 (0.13) | 0.16 |  |  |  |  |  |
|  | Surgery | Reference |  |  |  |  |  |  |
|  | - | - | - | 43.19% | 89.4% | <0.0001 | 0.1 | 10 |
|  | Intercept | 1.4 (0.42) | 0.0009 |  |  |  |  |  |
|  | Europe | "-0.35 (0.21) | 0.1 |  |  |  |  |  |
|  | South America | Reference |  |  |  |  |  |  |
|  | Oncology | "-0.48 (0.22) | 0.03 |  |  |  |  |  |
|  | Radiotherapy | "-0.19 (0.19) | 0.3 |  |  |  |  |  |
|  | Surgery | Reference |  |  |  |  |  |  |
|  | Week of survey beginning | "-0.005 (0.02) | 0.85 |  |  |  |  |  |
| **Routine PPE use (patients)** | - | - | - | 15.75% | 90.21% | <0.0001 | 0.5 | 6 |
|  | Intercept | 1.14 (0.28) | <0.0001 |  |  |  |  |  |
|  | Europe | 0.01 (0.32) | 0.96 |  |  |  |  |  |
|  | Japan | "-0.35 (0.4) | 0.37 |  |  |  |  |  |
|  | South America | Reference |  |  |  |  |  |  |
|  | - | - | - | 77.35% | 60.62% | 0.08 | 0.03 | 6 |
|  | Intercept | 0.73 (0.25) | 0.004 |  |  |  |  |  |
|  | Europe | 0.25 (0.2) | 0.23 |  |  |  |  |  |
|  | Japan | "-0.6 (0.25) | 0.01 |  |  |  |  |  |
|  | South America | Reference |  |  |  |  |  |  |
|  | Sample size | 0.004 (0.002) | 0.04 |  |  |  |  |  |
| **Routine PPE use (workers)** | - | - | - | 78.93% | 80.71% | 0.006 | <0.0001 | 10 |
|  | Intercept | "-2.53 (0.72) | 0.0004 |  |  |  |  |  |
|  | Asia | "-1.23 (0.36) | 0.0007 |  |  |  |  |  |
|  | Europe | 0.97 (0.29) | 0.0007 |  |  |  |  |  |
|  | Japan | 0.36 (0.3) | 0.23 |  |  |  |  |  |
|  | Middle East | "-0.19 (0.3) | 0.54 |  |  |  |  |  |
|  | North America | 0.31 (0.26) | 0.23 |  |  |  |  |  |
|  | South America | Reference |  |  |  |  |  |  |
|  | Week of survey end | 0.15 (0.03) | <0.0001 |  |  |  |  |  |
|  | Sample size | 0.006 (0.002) | 0.008 |  |  |  |  |  |
| **Remote consultations** | - | - | - | 29.74% | 92.31% | <0.0001 | 0.39 | 20 |
|  | Intercept | 1.7 (0.57) | 0.003 |  |  |  |  |  |
|  | Africa | "-0.13 (0.35) | 0.71 |  |  |  |  |  |
|  | Asia | "-0.81 (0.48) | 0.09 |  |  |  |  |  |
|  | Europe | 0.11 (0.26) | 0.67 |  |  |  |  |  |
|  | Middle East | 0.26 (0.33) | 0.43 |  |  |  |  |  |
|  | North America | 0.41 (0.33) | 0.21 |  |  |  |  |  |
|  | South America | Reference |  |  |  |  |  |  |
|  | Oncology | "-0.87 (0.53) | 0.1 |  |  |  |  |  |
|  | Radiotherapy | "-0.96 (0.51) | 0.06 |  |  |  |  |  |
|  | Surgery | Reference |  |  |  |  |  |  |
|  | Sample size | "-0.0006 (0.0009) | 0.48 |  |  |  |  |  |
|  | Operators | 0.25 (0.15) | 0.1 |  |  |  |  |  |
|  | Centers | Reference |  |  |  |  |  |  |
| **Routine swab screening** | - | - | - | 24.64% | 95.58% | <0.0001 | 0.13 | 17 |
|  | Intercept | 0.86 (0.11) | <0.0001 |  |  |  |  |  |
|  | Oncology | "-0.37 (0.16) | 0.02 |  |  |  |  |  |
|  | Radiotherapy | "-0.46 (0.31) | 0.13 |  |  |  |  |  |
|  | Surgery | Reference |  |  |  |  |  |  |
|  | Sample size | "-0.002 (0.0008) | 0.04 |  |  |  |  |  |
|  | Operators | 0.24 (0.16) | 0.14 |  |  |  |  |  |
|  | Centers | Reference |  |  |  |  |  |  |

1. **FUNNEL PLOTS**

**A: Delay of treatments**


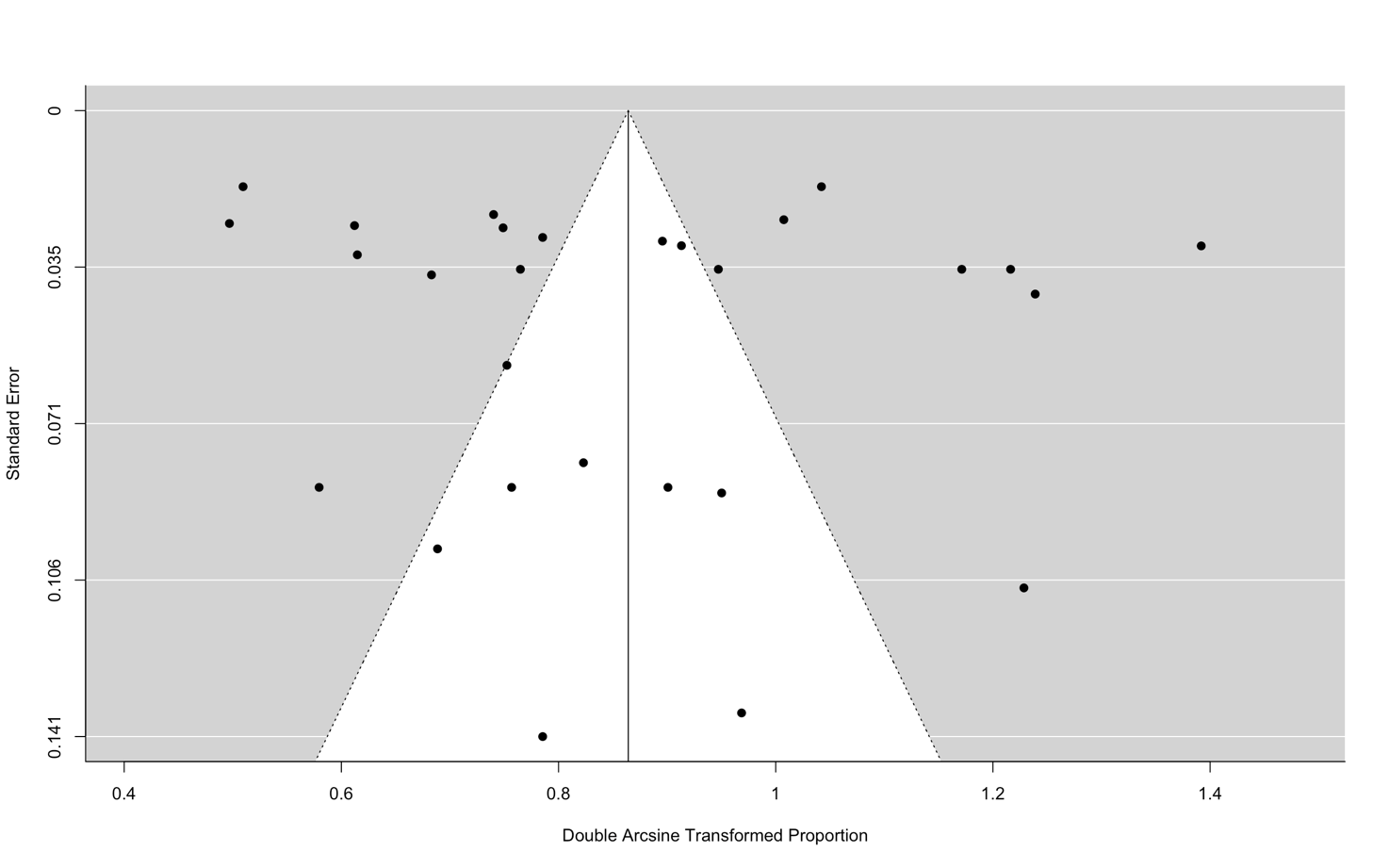


**B: Delay of visits**


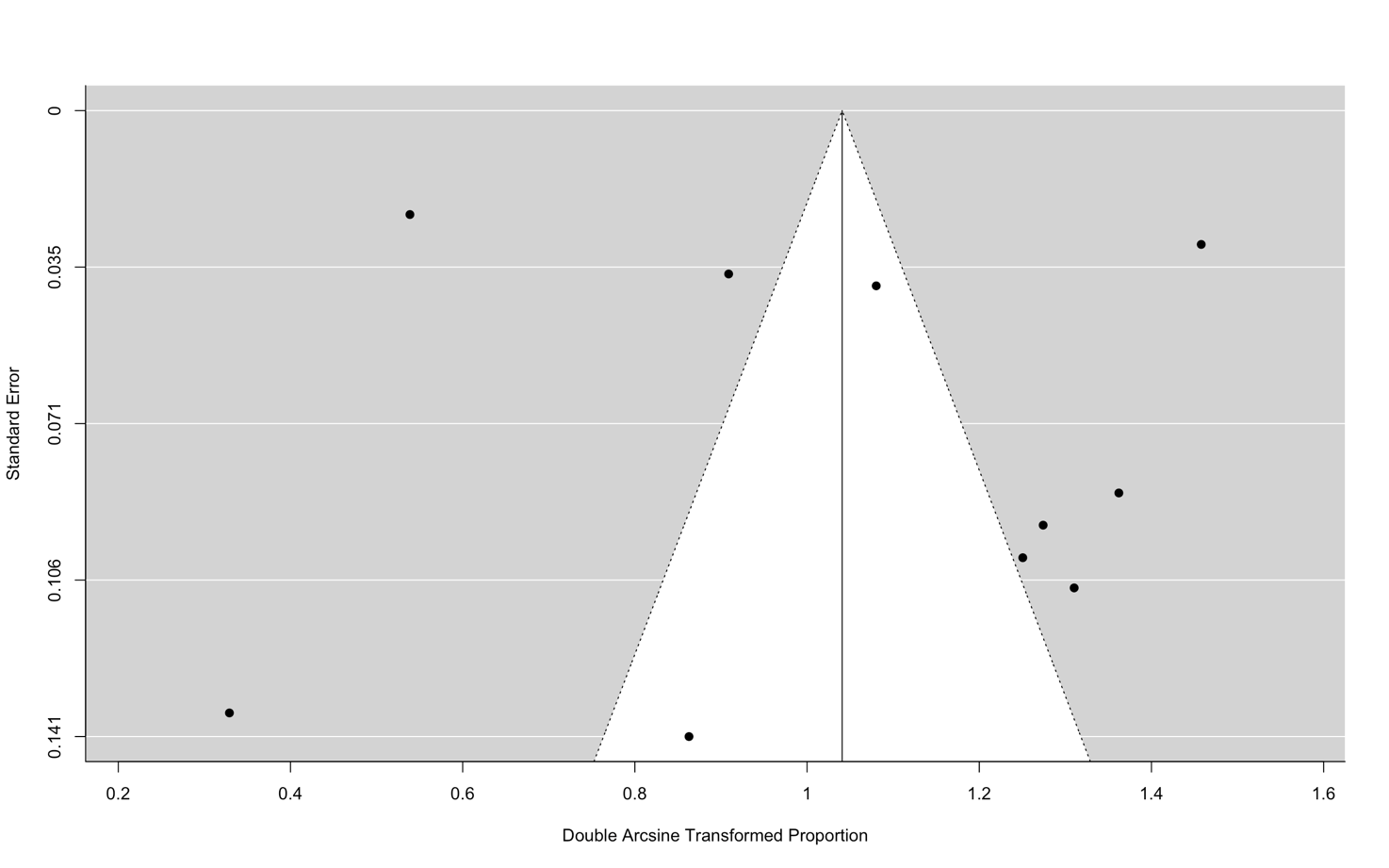


**C: Modification of treatments**


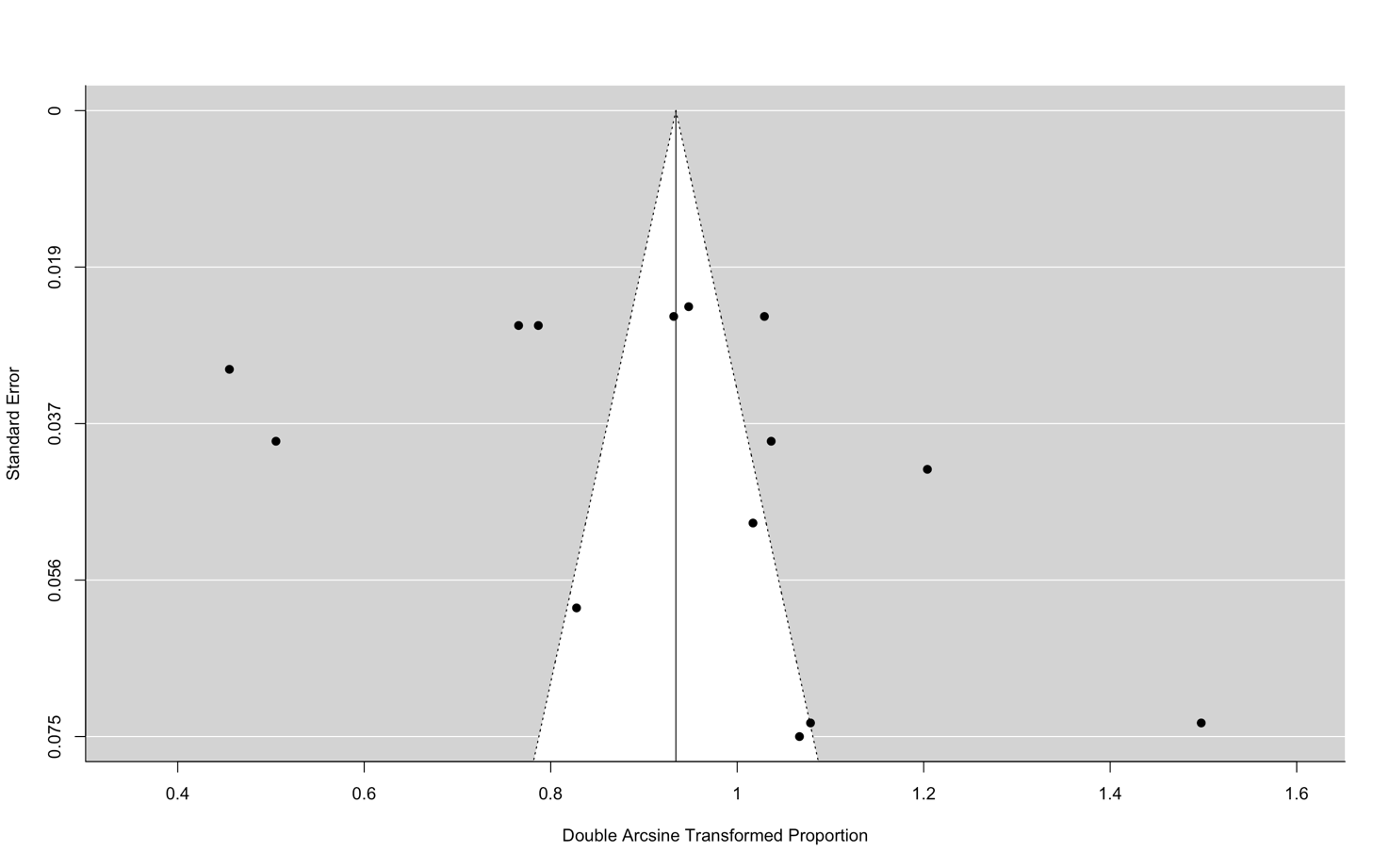


**D: PPE USE – PATIENTS**


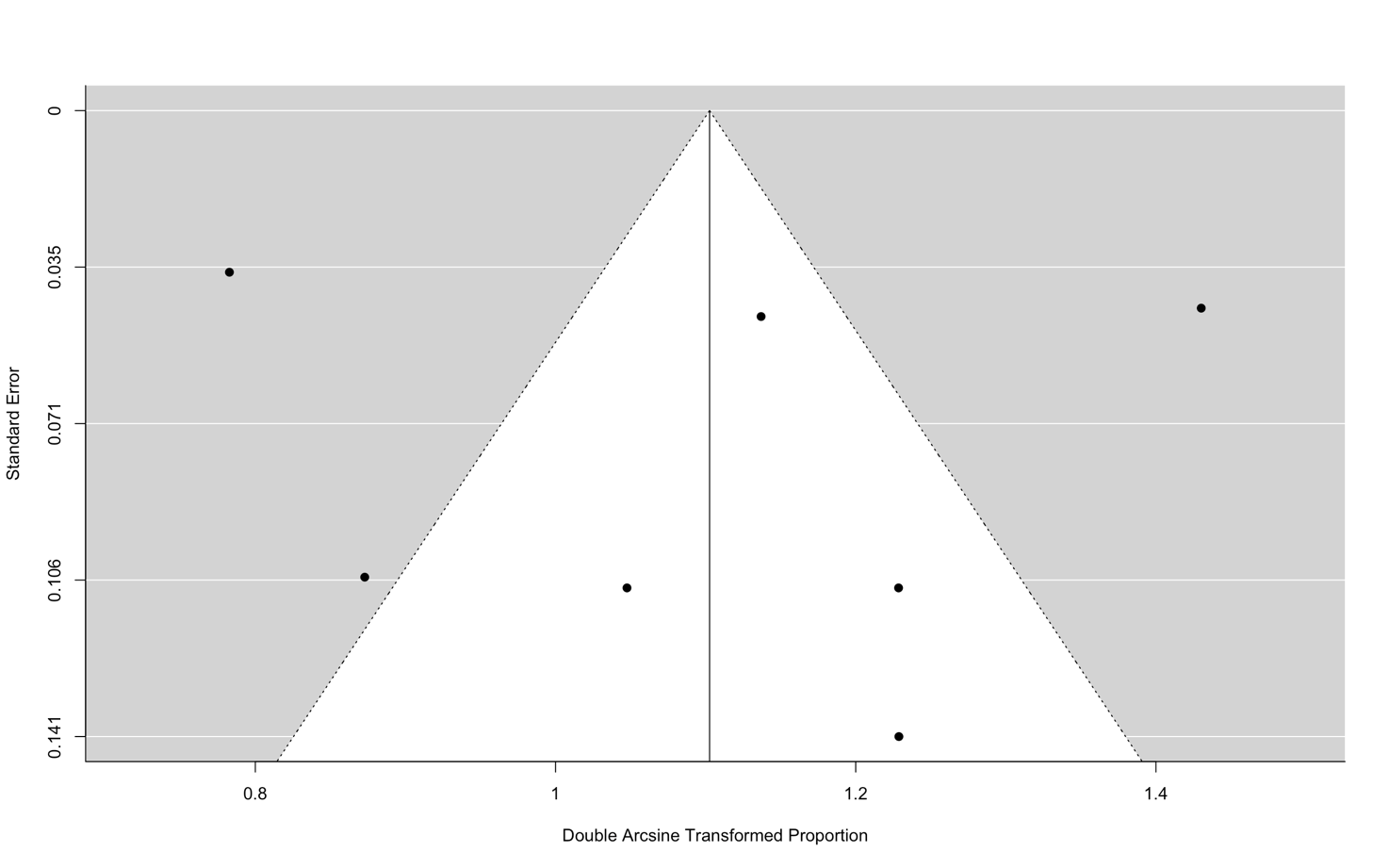


**E: PPE USE – WORKERS**


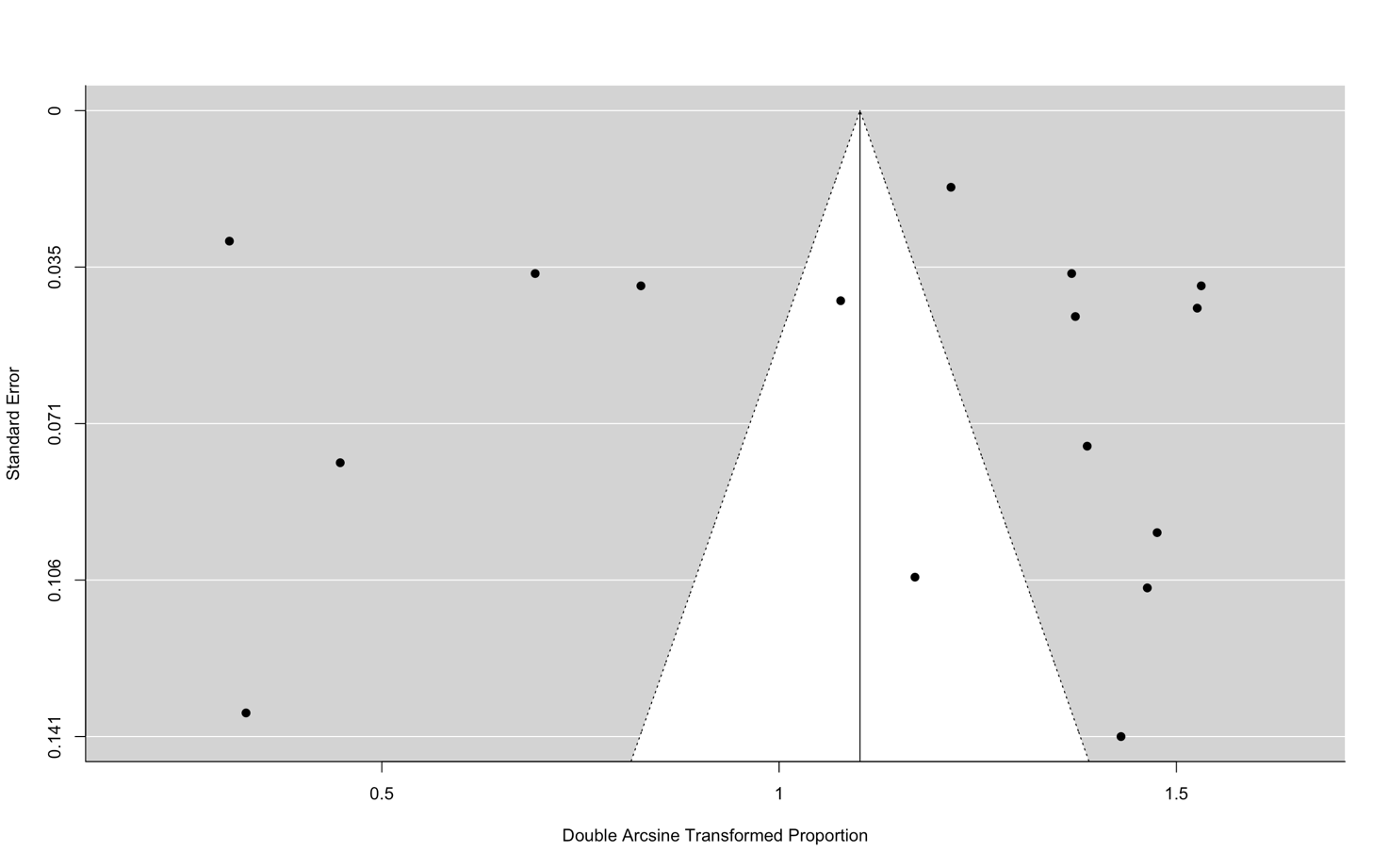


**F: REDUCTION OF ACTIVITY**


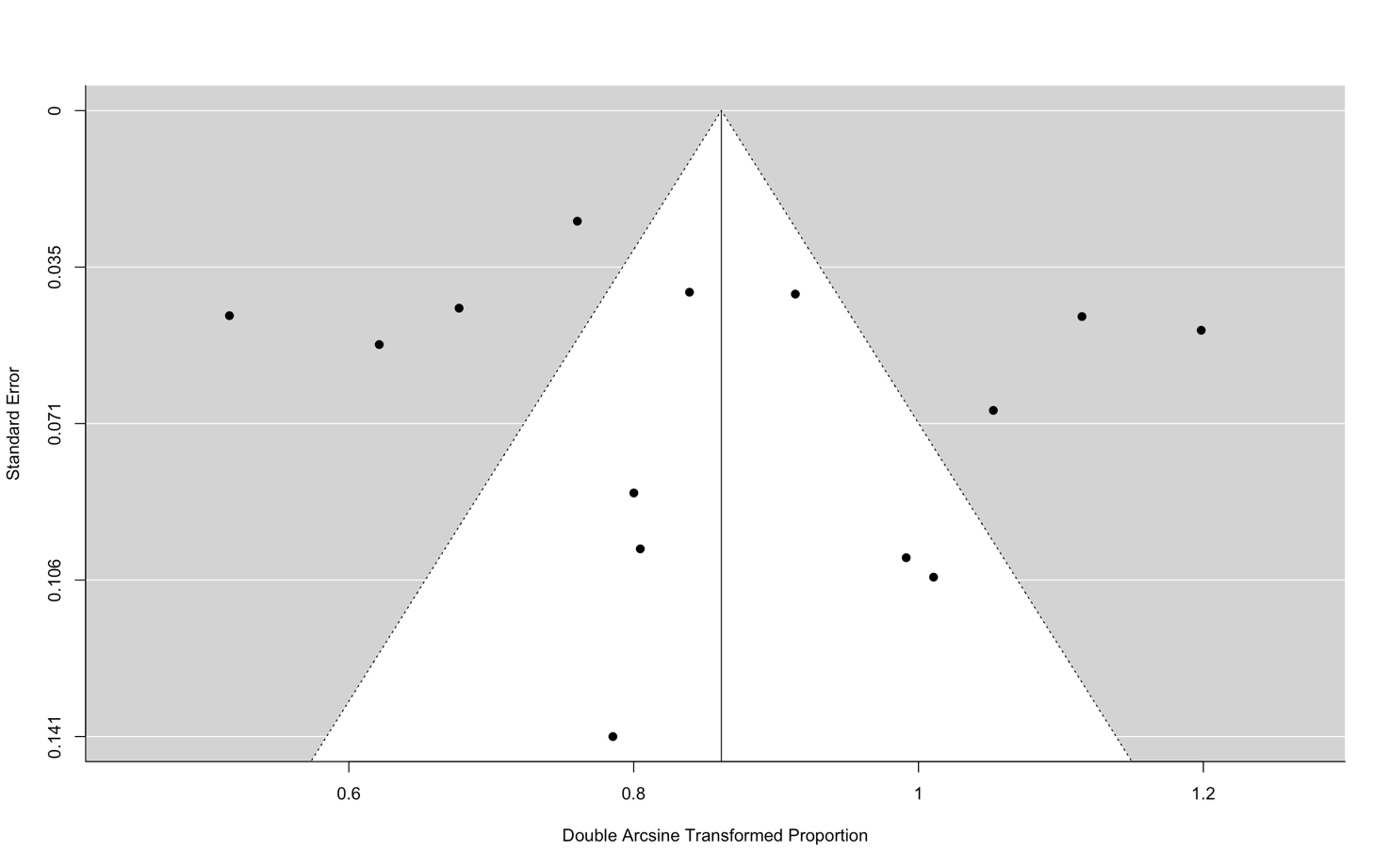


**G: REMOTE CONSULTATIONS**


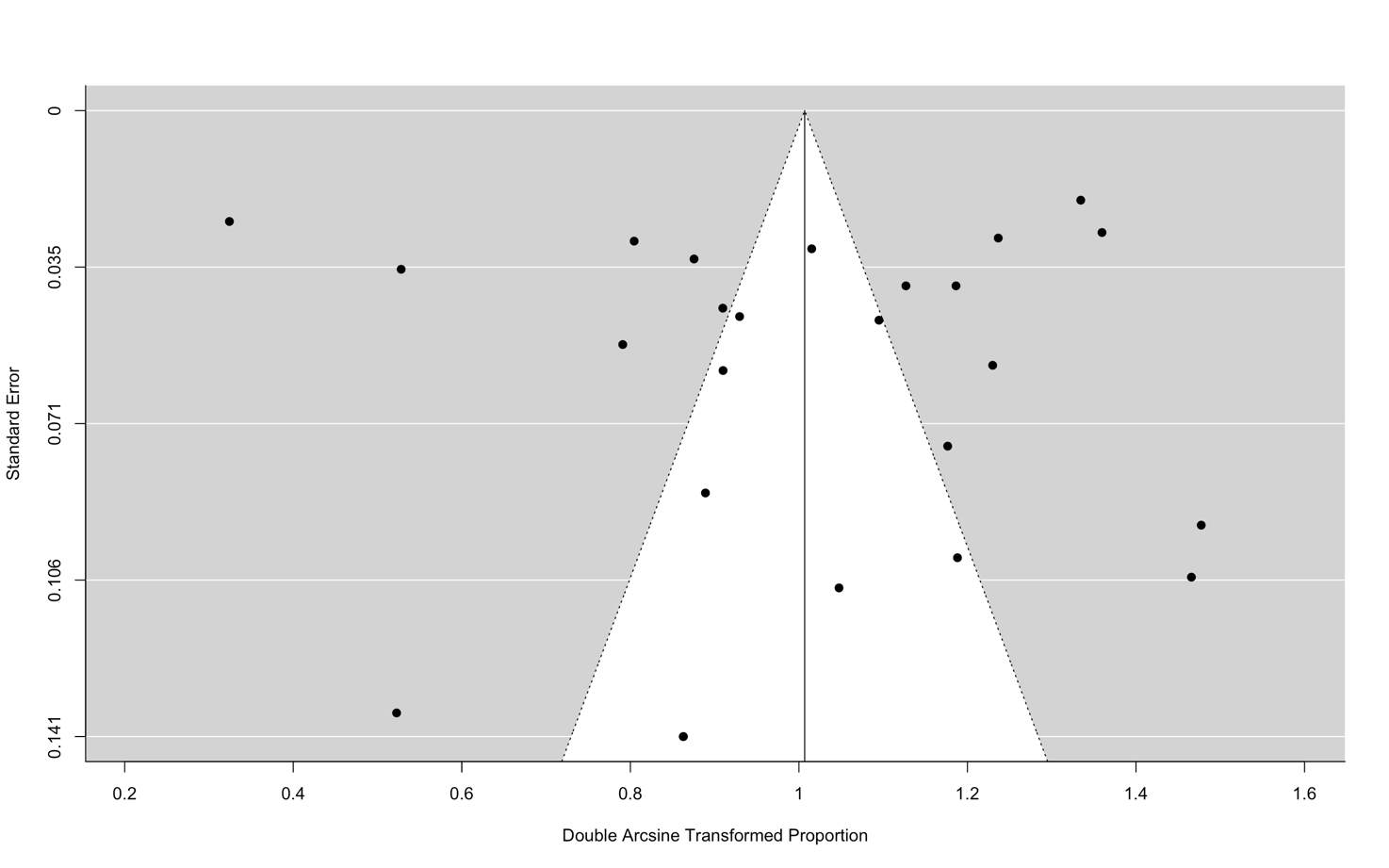


**H: ROUTINE SCREENING SWAB**


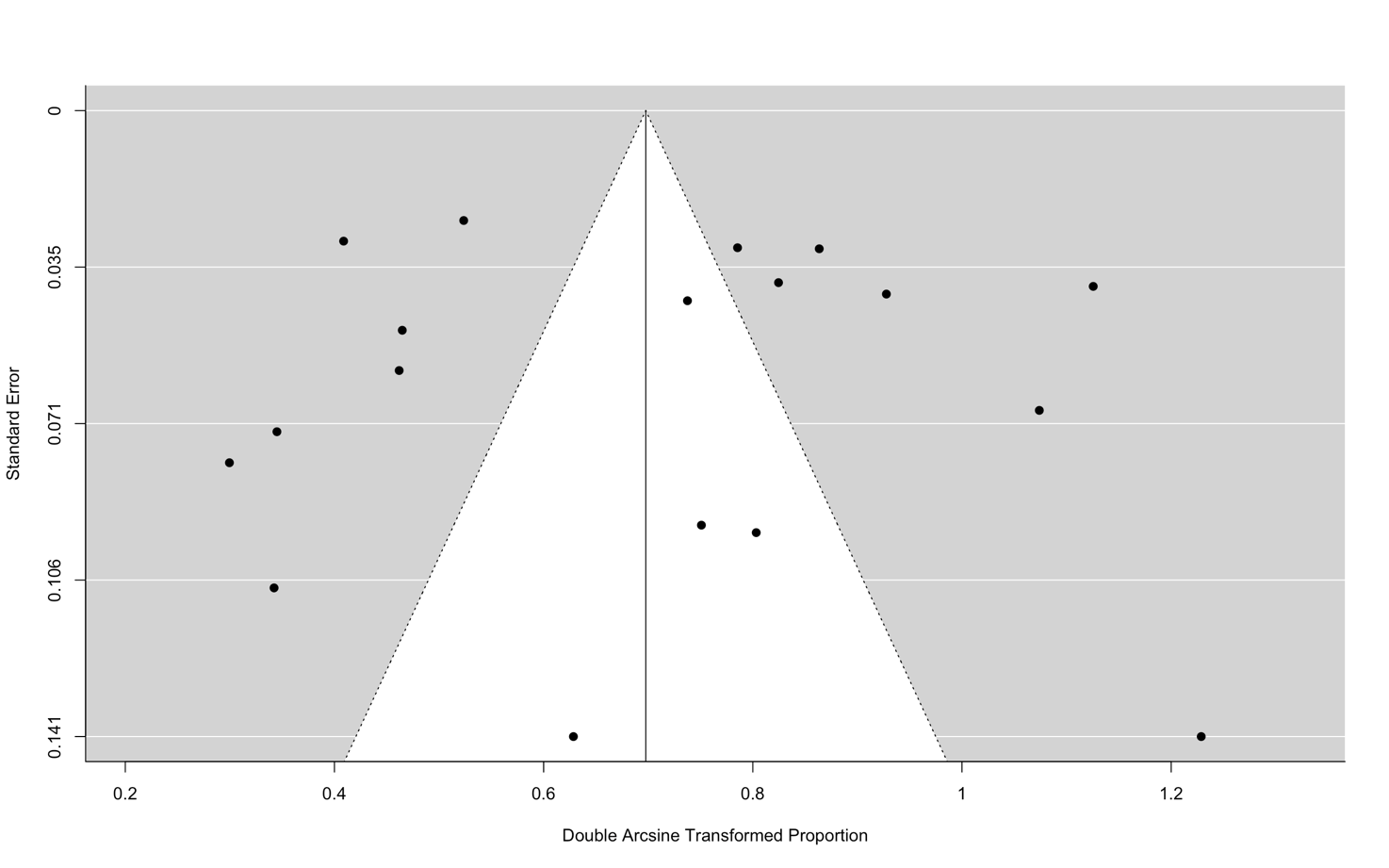


1. **OUTCOMES STRATIFIED BY WORLD REGION**

**A: DELAY OF TREATMENT**


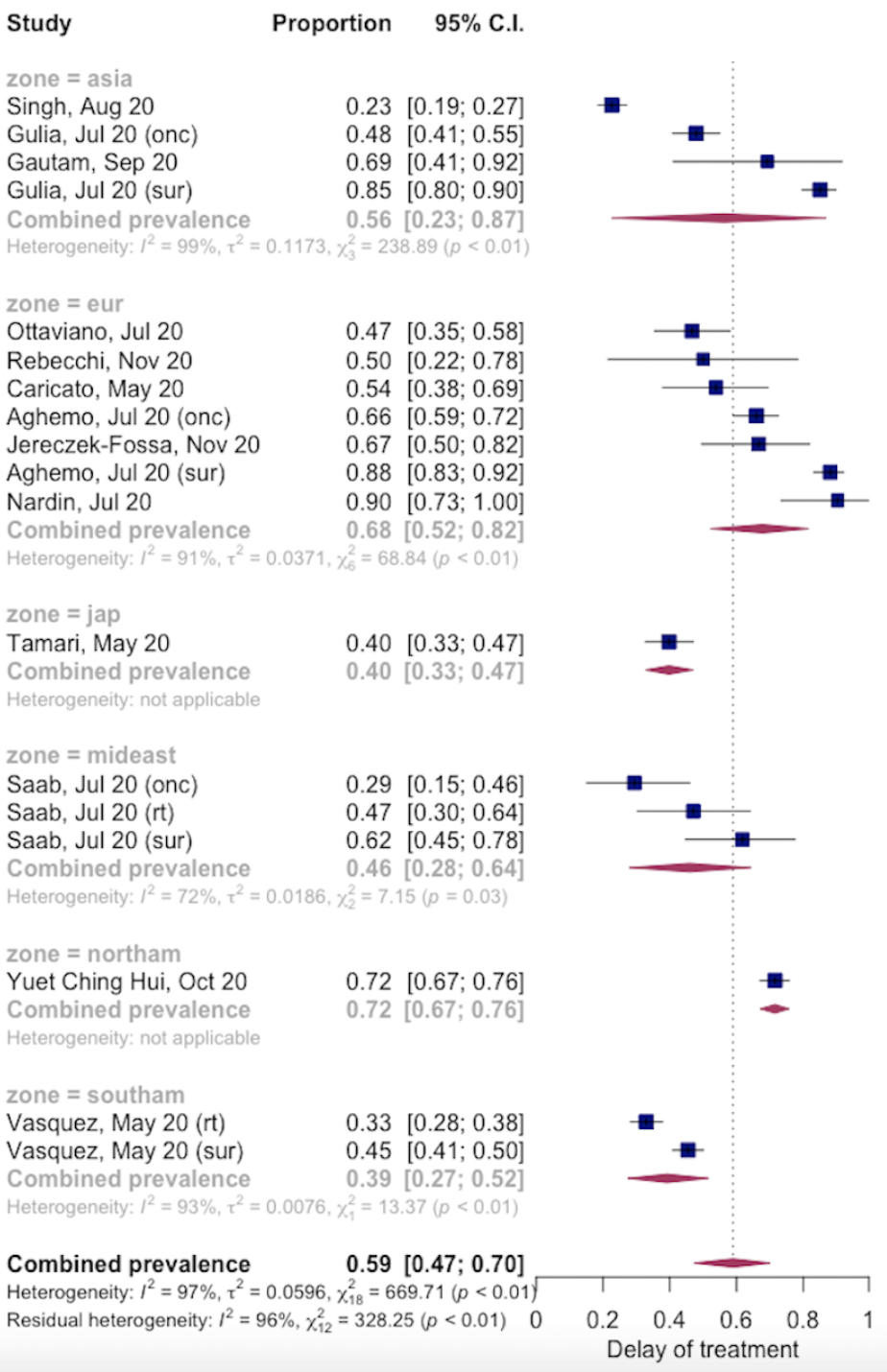


**B: DELAY OF VISITS**


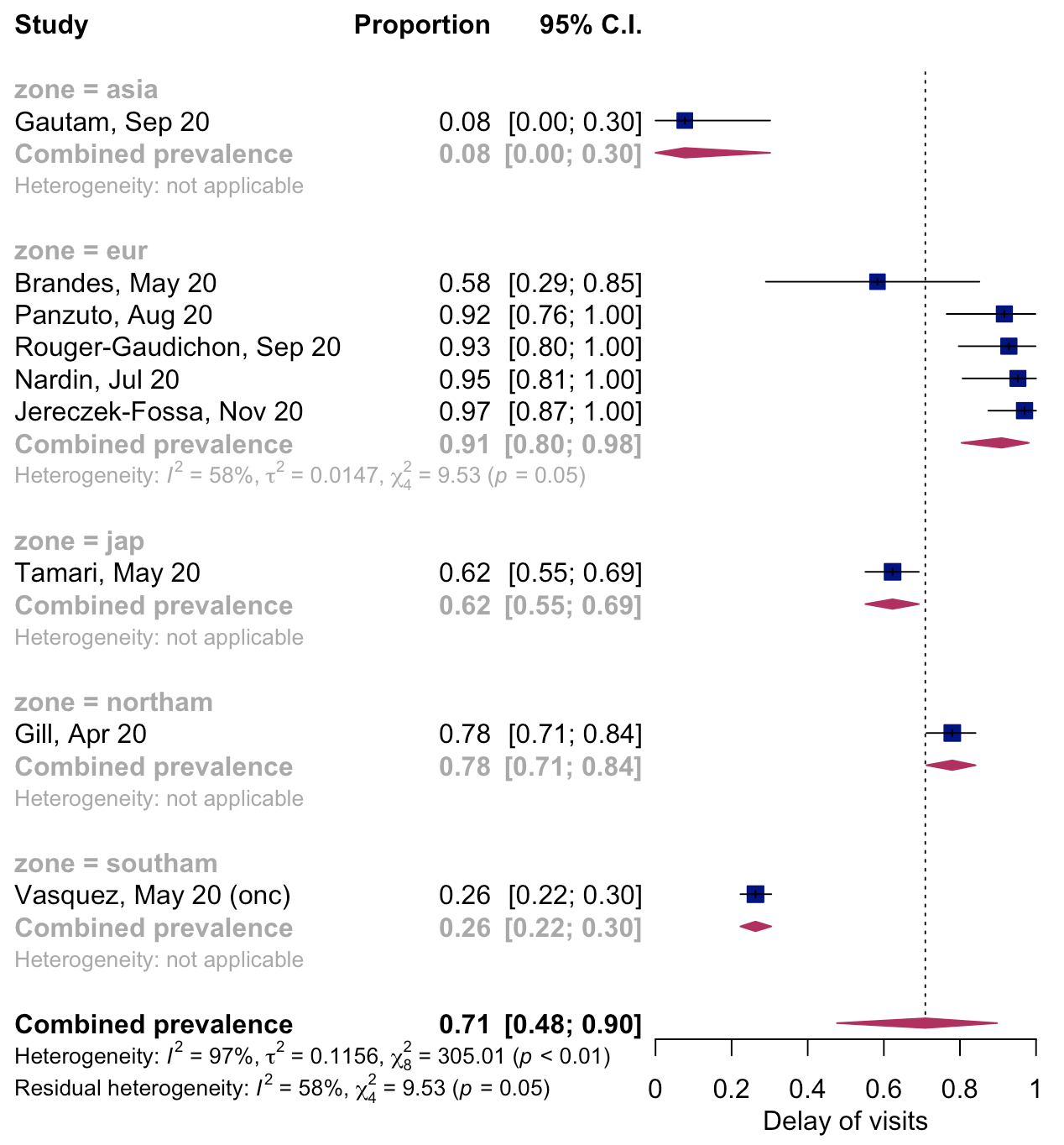


**C: MODIFICATION OF TREATMENT**


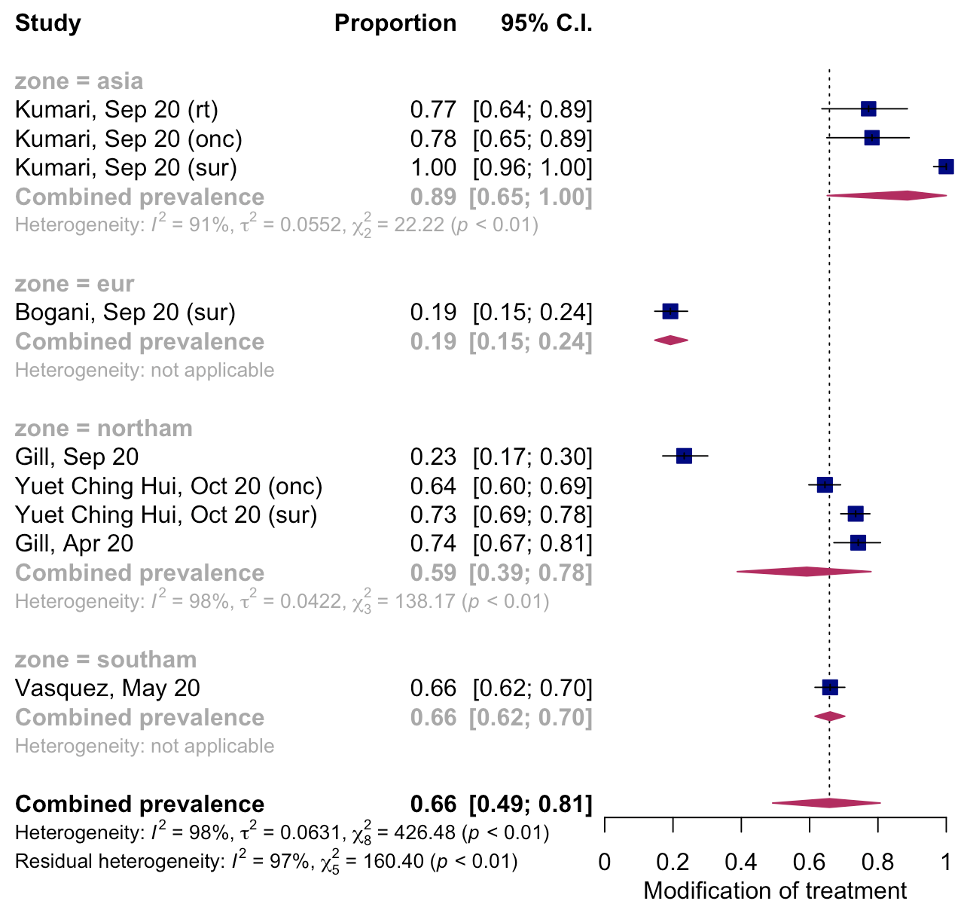


**D: PPE USE – PATIENTS**


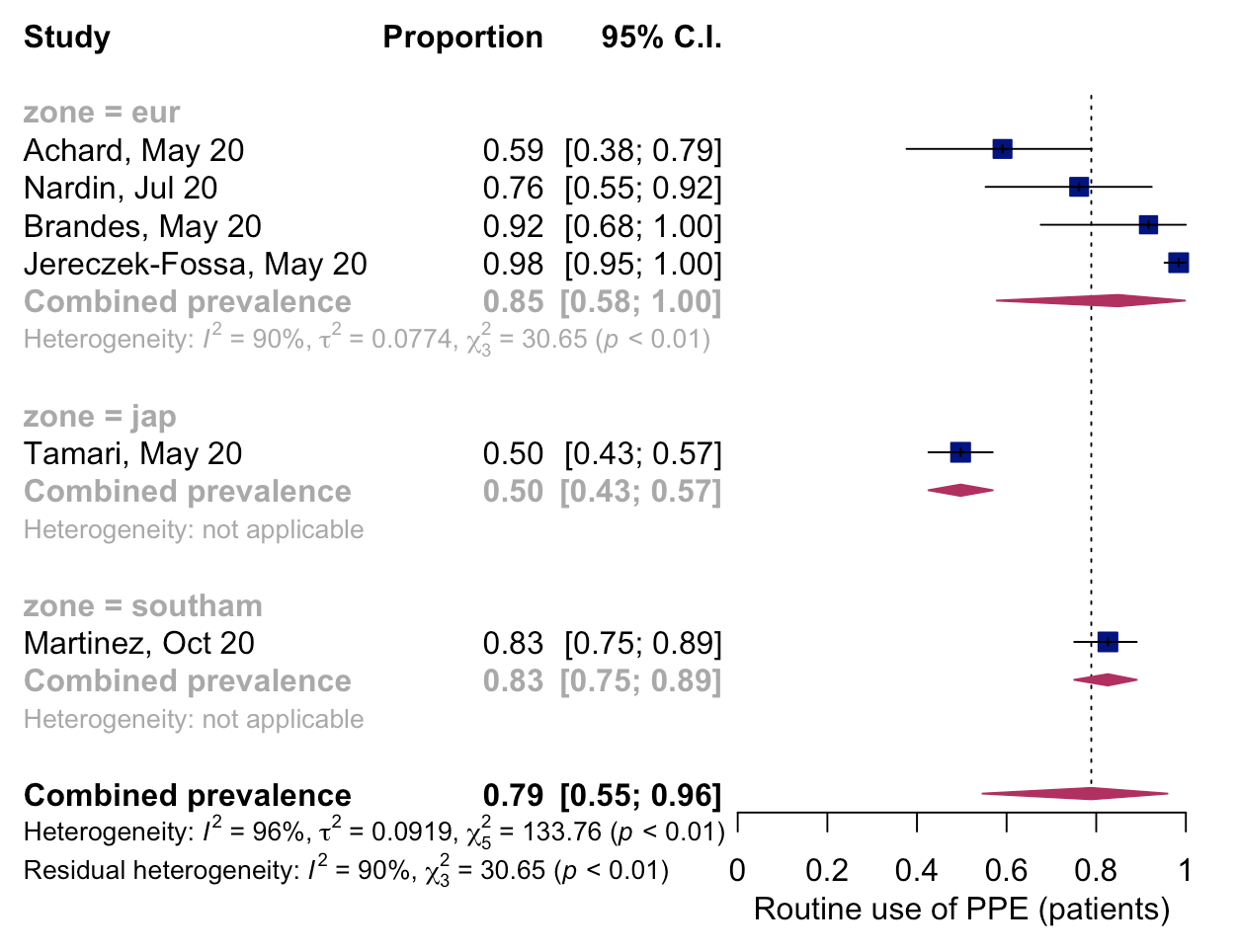


**E: PPE USE – WORKERS**


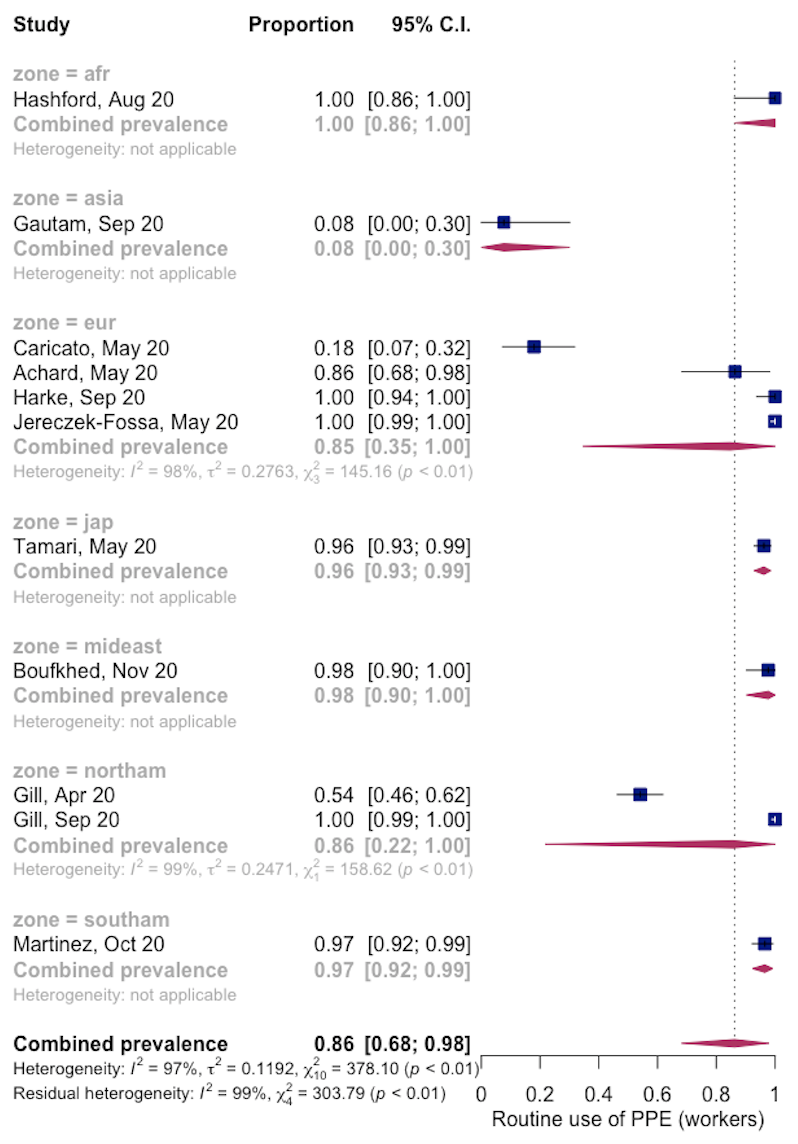


**F: REDUCTION OF ACTIVITY**


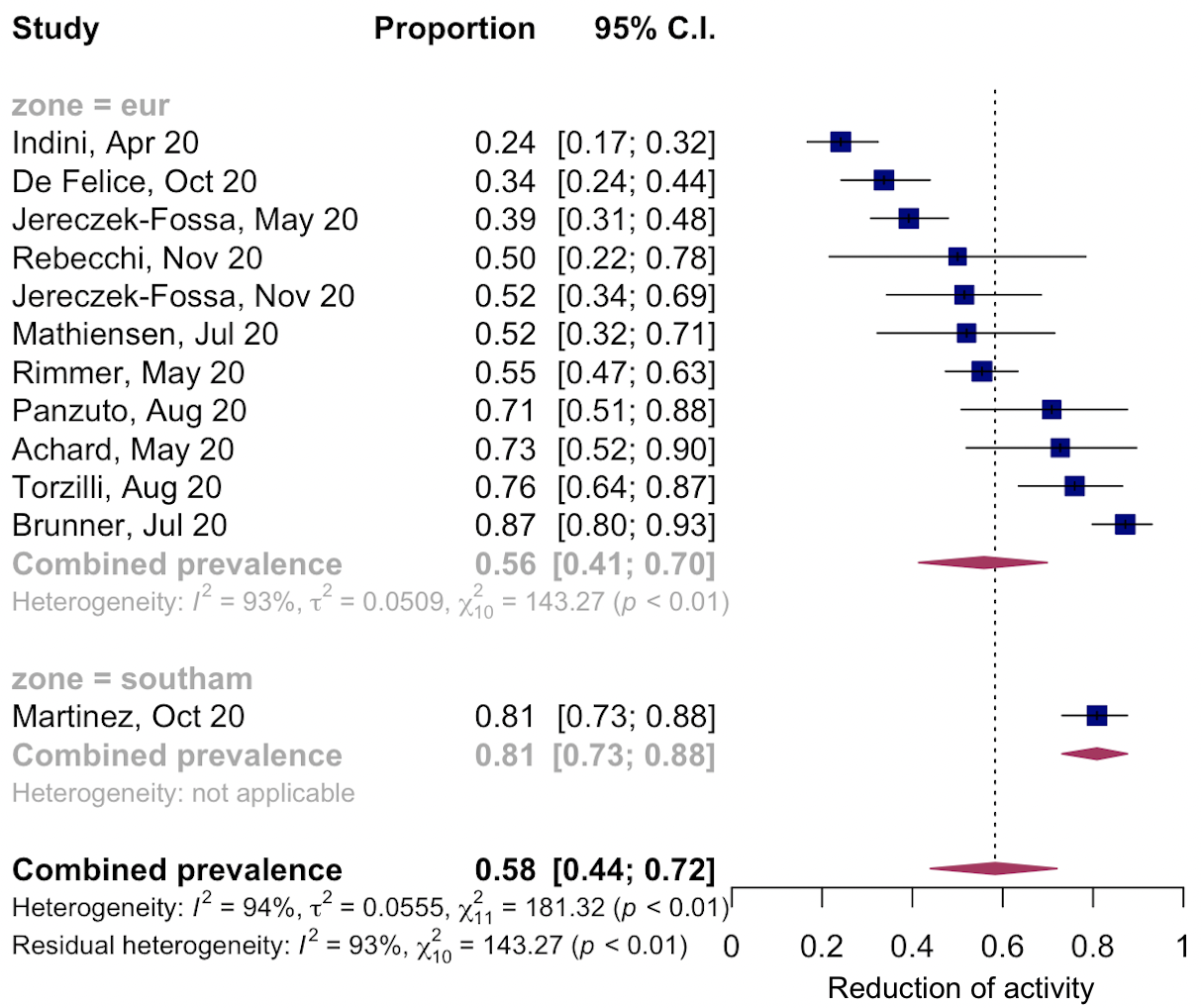


**G: REMOTE CONSULTATIONS**


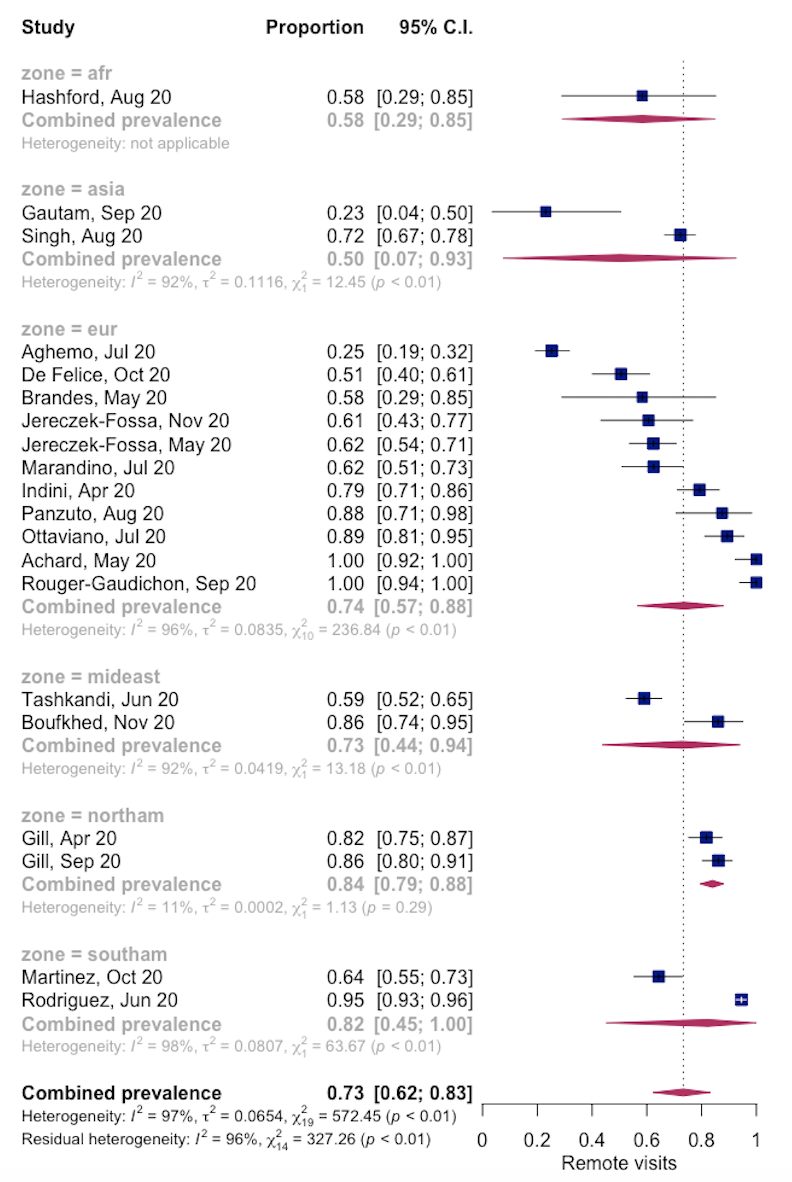


**H: ROUTINE SCREENING SWAB**


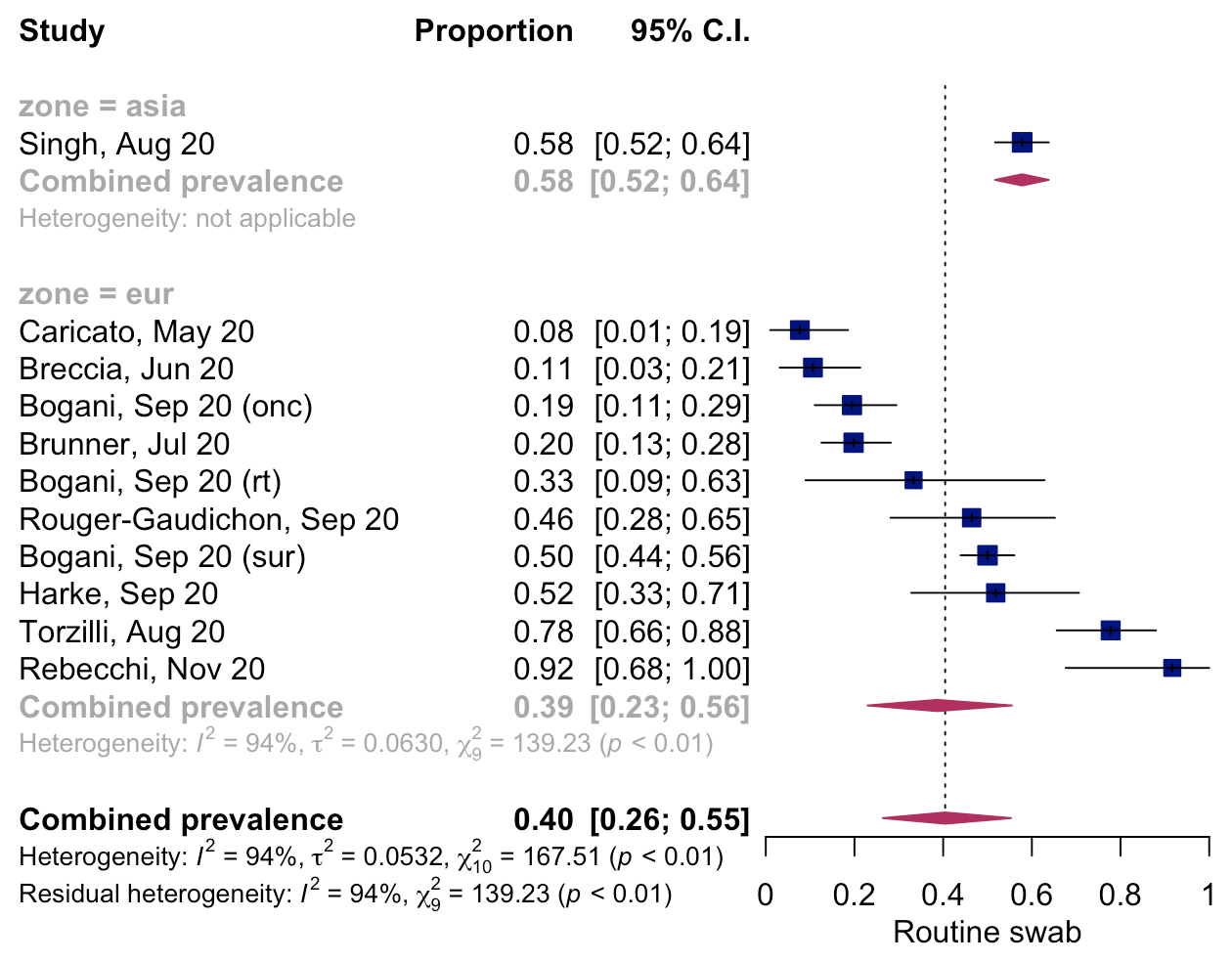


1. **METAREGRESSION FOR WORLD REGIONS**

**A: DELAY OF TREATMENTS**

Mixed-Effects Model (k = 18; tau^2 estimator: DL)

tau^2 (estimated amount of residual heterogeneity): 0.0564 (SE = 0.0340)

tau (square root of estimated tau^2 value): 0.2374

I^2 (residual heterogeneity / unaccounted variability): 96.34%

H^2 (unaccounted variability / sampling variability): 27.35

R^2 (amount of heterogeneity accounted for): 0.00%

Test for Residual Heterogeneity:

QE(df = 12) = 328.2454, p-val < .0001

Test of Moderators (coefficients 2:6):

QM(df = 5) = 3.8512, p-val = 0.5710

Model Results:

estimate se zval pval ci.lb ci.ub

intrcpt 0.6762 0.1688 4.0068 <.0001 0.3454 1.0070 ***

Asia 0.1660 0.2091 0.7936 0.4274 -0.2439 0.5758

Europe 0.2844 0.1937 1.4686 0.1419 -0.0952 0.6639

Japan 0.0068 0.2936 0.0232 0.9815 -0.5687 0.5823

Middle East 0.0695 0.2229 0.3116 0.7553 -0.3674 0.5063

North America 0.3312 0.2923 1.1331 0.2572 -0.2417 0.9041

South America Reference

**B: DELAY OF VISITS**

Mixed-Effects Model (k = 9; tau^2 estimator: DL)

tau^2 (estimated amount of residual heterogeneity): 0.0147 (SE = 0.0181)

tau (square root of estimated tau^2 value): 0.1211

I^2 (residual heterogeneity / unaccounted variability): 58.03%

H^2 (unaccounted variability / sampling variability): 2.38

R^2 (amount of heterogeneity accounted for): 87.32%

Test for Residual Heterogeneity:

QE(df = 4) = 9.5295, p-val = 0.0491

Test of Moderators (coefficients 2:5):

QM(df = 4) = 39.2619, p-val < .0001

Model Results:

estimate se zval pval ci.lb ci.ub

intrcpt 0.5387 0.1233 4.3688 <.0001 0.2970 0.7804 ***

Asia -0.2097 0.2200 -0.9532 0.3405 -0.6408 0.2214

Europe 0.6971 0.1426 4.8890 <.0001 0.4177 0.9766 ***

Japan 0.3702 0.1767 2.0951 0.0362 0.0239 0.7165 *

North America 0.5416 0.1773 3.0552 0.0022 0.1942 0.8891 **

South America Reference

**C: MODIFICATION OF TREATMENT**

Mixed-Effects Model (k = 9; tau^2 estimator: DL)

tau^2 (estimated amount of residual heterogeneity): 0.0435 (SE = 0.0353)

tau (square root of estimated tau^2 value): 0.2086

I^2 (residual heterogeneity / unaccounted variability): 96.88%

H^2 (unaccounted variability / sampling variability): 32.08

R^2 (amount of heterogeneity accounted for): 31.03%

Test for Residual Heterogeneity:

QE(df = 5) = 160.3997, p-val < .0001

Test of Moderators (coefficients 2:4):

QM(df = 3) = 10.2529, p-val = 0.0165

Model Results:

estimate se zval pval ci.lb ci.ub

intrcpt 0.9479 0.2099 4.5151 <.0001 0.5364 1.3594 ***

Asia 0.2666 0.2458 1.0848 0.2780 -0.2151 0.7483

Europe -0.4925 0.2976 -1.6549 0.0980 -1.0758 0.0908 .

North America -0.0711 0.2350 -0.3025 0.7623 -0.5317 0.3895

South America Reference

**D: PPE USE – PATIENTS**

Mixed-Effects Model (k = 6; tau^2 estimator: DL)

tau^2 (estimated amount of residual heterogeneity): 0.0774 (SE = 0.0786)

tau (square root of estimated tau^2 value): 0.2783

I^2 (residual heterogeneity / unaccounted variability): 90.21%

H^2 (unaccounted variability / sampling variability): 10.22

R^2 (amount of heterogeneity accounted for): 15.75%

Test for Residual Heterogeneity:

QE(df = 3) = 30.6538, p-val < .0001

Test of Moderators (coefficients 2:3):

QM(df = 2) = 1.3924, p-val = 0.4985

Model Results:

estimate se zval pval ci.lb ci.ub

intrcpt 1.1370 0.2821 4.0298 <.0001 0.5840 1.6899 ***

Europe 0.0143 0.3188 0.0448 0.9643 -0.6106 0.6391

Japan -0.3542 0.3980 -0.8901 0.3734 -1.1342 0.4258

South America Reference

**E: PPE USE – WORKERS**

Mixed-Effects Model (k = 11; tau^2 estimator: DL)

tau^2 (estimated amount of residual heterogeneity): 0.2601 (SE = 0.2312)

tau (square root of estimated tau^2 value): 0.5100

I^2 (residual heterogeneity / unaccounted variability): 98.68%

H^2 (unaccounted variability / sampling variability): 75.95

R^2 (amount of heterogeneity accounted for): 0.00%

Test for Residual Heterogeneity:

QE(df = 4) = 303.7861, p-val < .0001

Test of Moderators (coefficients 2:7):

QM(df = 6) = 3.2683, p-val = 0.7745

Model Results:

estimate se zval pval ci.lb ci.ub

intrcpt 1.3728 0.5121 2.6806 0.0073 0.3691 2.3766 **

Africa 0.0574 0.7365 0.0780 0.9378 -1.3861 1.5009

Asia -1.0438 0.7355 -1.4192 0.1559 -2.4853 0.3978

Europe -0.2171 0.5737 -0.3784 0.7051 -1.3414 0.9073

Japan -0.0047 0.7237 -0.0064 0.9949 -1.4231 1.4138

Middle East 0.0149 0.7268 0.0205 0.9837 -1.4095 1.4393

North America -0.1942 0.6270 -0.3097 0.7568 -1.4231 1.0347

South America Reference

**F: REDUCTION OF ACTIVITY**

Mixed-Effects Model (k = 12; tau^2 estimator: DL)

tau^2 (estimated amount of residual heterogeneity): 0.0509 (SE = 0.0289)

tau (square root of estimated tau^2 value): 0.2257

I^2 (residual heterogeneity / unaccounted variability): 93.02%

H^2 (unaccounted variability / sampling variability): 14.33

R^2 (amount of heterogeneity accounted for): 8.18%

Test for Residual Heterogeneity:

QE(df = 10) = 143.2681, p-val < .0001

Test of Moderators (coefficient 2):

QM(df = 1) = 1.2719, p-val = 0.2594

Model Results:

estimate se zval pval ci.lb ci.ub

intrcpt 1.1148 0.2304 4.8379 <.0001 0.6631 1.5664 ***

Europe -0.2723 0.2414 -1.1278 0.2594 -0.7455 0.2009

South America Reference

**G: REMOTE CONSULTATIONS**

Mixed-Effects Model (k = 20; tau^2 estimator: DL)

tau^2 (estimated amount of residual heterogeneity): 0.0692 (SE = 0.0332)

tau (square root of estimated tau^2 value): 0.2631

I^2 (residual heterogeneity / unaccounted variability): 95.72%

H^2 (unaccounted variability / sampling variability): 23.38

R^2 (amount of heterogeneity accounted for): 0.00%

Test for Residual Heterogeneity:

QE(df = 14) = 327.2605, p-val < .0001

Test of Moderators (coefficients 2:6):

QM(df = 5) = 2.4639, p-val = 0.7819

Model Results:

estimate se zval pval ci.lb ci.ub

intrcpt 1.1346 0.1878 6.0424 <.0001 0.7665 1.5026 ***

Africa -0.2717 0.3528 -0.7700 0.4413 -0.9632 0.4198

Asia -0.3383 0.2725 -1.2415 0.2144 -0.8724 0.1958

Europe -0.1092 0.2052 -0.5320 0.5948 -0.5114 0.2931

Middle East -0.1133 0.2675 -0.4235 0.6719 -0.6376 0.4110

North America 0.0221 0.2658 0.0833 0.9336 -0.4988 0.5431

South America Reference

**H: ROUTINE SCREENING SWAB**

Mixed-Effects Model (k = 11; tau^2 estimator: DL)

tau^2 (estimated amount of residual heterogeneity): 0.0630 (SE = 0.0410)

tau (square root of estimated tau^2 value): 0.2510

I^2 (residual heterogeneity / unaccounted variability): 93.54%

H^2 (unaccounted variability / sampling variability): 15.47

R^2 (amount of heterogeneity accounted for): 0.00%

Test for Residual Heterogeneity:

QE(df = 9) = 139.2268, p-val < .0001

Test of Moderators (coefficient 2):

QM(df = 1) = 0.5039, p-val = 0.4778

Model Results:

estimate se zval pval ci.lb ci.ub

intrcpt 0.6744 0.0840 8.0287 <.0001 0.5097 0.8390 ***

Asia 0.1892 0.2665 0.7099 0.4778 -0.3331 0.7115

Europe Reference

1. **METAREGRESSION FOR SPECIALTY**

**A: DELAY OF TREATMENT**

Mixed-Effects Model (k = 28; tau^2 estimator: DL)

tau^2 (estimated amount of residual heterogeneity): 0.0746 (SE = 0.0284)

tau (square root of estimated tau^2 value): 0.2732

I^2 (residual heterogeneity / unaccounted variability): 98.41%

H^2 (unaccounted variability / sampling variability): 63.04

R^2 (amount of heterogeneity accounted for): 0.00%

Test for Residual Heterogeneity:

QE(df = 25) = 1575.9655, p-val < .0001

Test of Moderators (coefficients 2:3):

QM(df = 2) = 1.0069, p-val = 0.6044

Model Results:

estimate se zval pval ci.lb ci.ub

intrcpt 0.9082 0.0726 12.5093 <.0001 0.7659 1.0505 ***

Oncology -0.0595 0.1280 -0.4647 0.6422 -0.3104 0.1914

Radiotherapy -0.1334 0.1349 -0.9888 0.3228 -0.3977 0.1310

Surgery Reference

**B: DELAY OF VISITS**

Mixed-Effects Model (k = 10; tau^2 estimator: DL)

tau^2 (estimated amount of residual heterogeneity): 0.2271 (SE = 0.1859)

tau (square root of estimated tau^2 value): 0.4765

I^2 (residual heterogeneity / unaccounted variability): 98.93%

H^2 (unaccounted variability / sampling variability): 93.63

R^2 (amount of heterogeneity accounted for): 0.00%

Test for Residual Heterogeneity:

QE(df = 7) = 655.3852, p-val < .0001

Test of Moderators (coefficients 2:3):

QM(df = 2) = 2.2800, p-val = 0.3198

Model Results:

estimate se zval pval ci.lb ci.ub

intrcpt 0.3291 0.4956 0.6640 0.5067 -0.6422 1.3004

Oncology 0.7814 0.5283 1.4790 0.1391 -0.2541 1.8168

Radiotherapy 0.8035 0.6011 1.3367 0.1813 -0.3747 1.9816

Surgery Reference

**C: MODIFICATION OF TREATMENT**

Mixed-Effects Model (k = 14; tau^2 estimator: DL)

tau^2 (estimated amount of residual heterogeneity): 0.0576 (SE = 0.0305)

tau (square root of estimated tau^2 value): 0.2400

I^2 (residual heterogeneity / unaccounted variability): 97.84%

H^2 (unaccounted variability / sampling variability): 46.34

R^2 (amount of heterogeneity accounted for): 0.00%

Test for Residual Heterogeneity:

QE(df = 11) = 509.7377, p-val < .0001

Test of Moderators (coefficients 2:3):

QM(df = 2) = 0.7269, p-val = 0.6953

Model Results:

estimate se zval pval ci.lb ci.ub

intrcpt 1.0408 0.1417 7.3439 <.0001 0.7631 1.3186 ***

Oncology -0.1421 0.1690 -0.8409 0.4004 -0.4734 0.1891

Radiotherapy -0.1203 0.1874 -0.6419 0.5210 -0.4876 0.2470

Surgery Reference

**D: PPE USE – PATIENTS**

Mixed-Effects Model (k = 7; tau^2 estimator: DL)

tau^2 (estimated amount of residual heterogeneity): 0.0939 (SE = 0.0802)

tau (square root of estimated tau^2 value): 0.3065

I^2 (residual heterogeneity / unaccounted variability): 96.27%

H^2 (unaccounted variability / sampling variability): 26.81

R^2 (amount of heterogeneity accounted for): 0.00%

Test for Residual Heterogeneity:

QE(df = 5) = 134.0505, p-val < .0001

Test of Moderators (coefficient 2):

QM(df = 1) = 0.1911, p-val = 0.6620

Model Results:

estimate se zval pval ci.lb ci.ub

intrcpt 1.1668 0.1899 6.1433 <.0001 0.7945 1.5390 ***

Radiotherapy -0.0538 0.1230 -0.4371 0.6620 -0.2949 0.1874

Oncology Reference

**E: PPE USE – WORKERS**

Mixed-Effects Model (k = 16; tau^2 estimator: DL)

tau^2 (estimated amount of residual heterogeneity): 0.1671 (SE = 0.0972)

tau (square root of estimated tau^2 value): 0.4087

I^2 (residual heterogeneity / unaccounted variability): 98.75%

H^2 (unaccounted variability / sampling variability): 80.25

R^2 (amount of heterogeneity accounted for): 3.55%

Test for Residual Heterogeneity:

QE(df = 13) = 1043.2693, p-val < .0001

Test of Moderators (coefficients 2:3):

QM(df = 2) = 6.8473, p-val = 0.0326

Model Results:

estimate se zval pval ci.lb ci.ub

intrcpt 0.7959 0.1569 5.0725 <.0001 0.4884 1.1034 ***

Oncology 0.5034 0.2601 1.9355 0.0529 -0.0064 1.0131 .

Radiotherapy 0.5788 0.2438 2.3743 0.0176 0.1010 1.0566 *

Surgery Reference

**F: REDUCTION OF ACTIVITY**

Mixed-Effects Model (k = 14; tau^2 estimator: DL)

tau^2 (estimated amount of residual heterogeneity): 0.0381 (SE = 0.0220)

tau (square root of estimated tau^2 value): 0.1951

I^2 (residual heterogeneity / unaccounted variability): 93.07%

H^2 (unaccounted variability / sampling variability): 14.44

R^2 (amount of heterogeneity accounted for): 0.00%

Test for Residual Heterogeneity:

QE(df = 11) = 158.7906, p-val < .0001

Test of Moderators (coefficients 2:3):

QM(df = 2) = 1.2536, p-val = 0.5343

Model Results:

estimate se zval pval ci.lb ci.ub

intrcpt 0.9134 0.0785 11.6372 <.0001 0.7596 1.0673 ***

Oncology -0.1813 0.1676 -1.0814 0.2795 -0.5099 0.1473

Radiotherapy -0.0733 0.1214 -0.6038 0.5460 -0.3113 0.1646

Surgery Reference

**G: REMOTE CONSULTATIONS**

Mixed-Effects Model (k = 25; tau^2 estimator: DL)

tau^2 (estimated amount of residual heterogeneity): 0.1302 (SE = 0.0551)

tau (square root of estimated tau^2 value): 0.3609

I^2 (residual heterogeneity / unaccounted variability): 98.62%

H^2 (unaccounted variability / sampling variability): 72.72

R^2 (amount of heterogeneity accounted for): 0.00%

Test for Residual Heterogeneity:

QE(df = 22) = 1599.9243, p-val < .0001

Test of Moderators (coefficients 2:3):

QM(df = 2) = 0.9622, p-val = 0.6181

Model Results:

estimate se zval pval ci.lb ci.ub

intrcpt 0.9011 0.1492 6.0382 <.0001 0.6086 1.1935 ***

Oncology 0.1710 0.1808 0.9456 0.3444 -0.1834 0.5254

Radiotherapy 0.0712 0.2126 0.3352 0.7375 -0.3454 0.4879

Surgery Reference

**H: ROUTINE SCREENING SWAB**

Mixed-Effects Model (k = 18; tau^2 estimator: DL)

tau^2 (estimated amount of residual heterogeneity): 0.0556 (SE = 0.0268)

tau (square root of estimated tau^2 value): 0.2359

I^2 (residual heterogeneity / unaccounted variability): 96.63%

H^2 (unaccounted variability / sampling variability): 29.64

R^2 (amount of heterogeneity accounted for): 4.21%

Test for Residual Heterogeneity:

QE(df = 15) = 444.5780, p-val < .0001

Test of Moderators (coefficients 2:3):

QM(df = 2) = 4.2913, p-val = 0.1170

Model Results:

estimate se zval pval ci.lb ci.ub

intrcpt 0.7667 0.0675 11.3539 <.0001 0.6344 0.8991 ***

Oncology -0.2925 0.1423 -2.0555 0.0398 -0.5714 -0.0136 *

Radiotherapy -0.1383 0.2832 -0.4883 0.6254 -0.6933 0.4168

Surgery Reference
